# Supplementary material for: Embedding electronic patient-reported outcome measures into routine care for patients with stage III MELanoma (ePROMs-MEL): protocol for a prospective, longitudinal, mixed-methods pilot study
Source: BMJ Open. 2022 Dec 20;12(12):e066852. doi: 10.1136/bmjopen-2022-066852 (PMC9772660; doi:10.1136/bmjopen-2022-066852)
Supplement: Supplementary data [file bmjopen-2022-066852supp001.pdf]

NCCN National Comprehensive Cancer Network®

APPENDIX 1

NCCN Distress Thermometer and Problem List for Patients

NCCN DISTRESS THERMOMETER

Instructions: Please circle the number (0–10) that best describes how much distress you have been experiencing in the past week including today.

Extreme distress

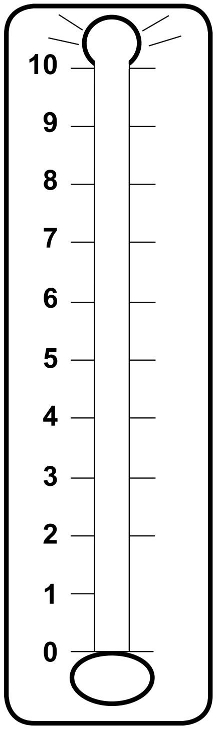

No distress

PROBLEM LIST

Please indicate if any of the following has been a problem for you in the past week including today.

Be sure to check YES or NO for each.

YES	NO	**Practical Problems**
❑	❑	Child care
❑	❑	Housing
❑	❑	Insurance/financial
❑	❑	Transportation
❑	❑	Work/school
❑	❑	Treatment decisions
		**Family Problems**
❑	❑	Dealing with children
❑	❑	Dealing with partner
❑	❑	Ability to have children
❑	❑	Family health issues
		**Emotional Problems**
❑	❑	Depression
❑	❑	Fears
❑	❑	Nervousness
❑	❑	Sadness
❑	❑	Worry
❑	❑	Loss of interest in usual activities
❑	❑	**Spiritual/religious concerns**

YES	NO	**Physical Problems**
❑	❑	Appearance
❑	❑	Bathing/dressing
❑	❑	Breathing
❑	❑	Changes in urination
❑	❑	Constipation
❑	❑	Diarrhea
❑	❑	Eating
❑	❑	Fatigue
❑	❑	Feeling swollen
❑	❑	Fevers
❑	❑	Getting around
❑	❑	Indigestion
❑	❑	Memory/concentration
❑	❑	Mouth sores
❑	❑	Nausea
❑	❑	Nose dry/congested
❑	❑	Pain
❑	❑	Sexual
❑	❑	Skin dry/itchy
❑	❑	Sleep
❑	❑	Substance use
❑	❑	Tingling in hands/feet

Other Problems: ______________________________

____

Version 2.2018, 02/23/18. The NCCN Clinical Practice Guidelines (NCCN Guidelines®) are a statement of evidence and consensus of the authors regarding their views of currently accepted approaches to treatment. Any clinician seeking to apply or consult the NCCN Guidelines is expected to use independent medical judgment in the context of individual clinical circumstances to determine any patient's care or treatment. The National Comprehensive Cancer Network® (NCCN®) makes no representations or warranties of any kind regarding their content, use or application and disclaims any responsibility for their application or use in any way. The NCCN Guidelines are copyrighted by National Comprehensive Cancer Network®. All rights reserved. The NCCN Guidelines and the illustrations herein may not be reproduced in any form without the express written permission of NCCN. ©2018.

EQ-5D-5L (UK English sample version)

Under each heading, please tick the **ONE** box that best describes your health **TODAY**

MOBILITY

I have no problems in walking about

I have slight problems in walking about

I have moderate problems in walking about

I have severe problems in walking about

I am unable to walk about

SELF-CARE

I have no problems washing or dressing myself

I have slight problems washing or dressing myself

I have moderate problems washing or dressing myself

I have severe problems washing or dressing myself

I am unable to wash or dress myself

USUAL ACTIVITIES *(e.g. work, study, housework, family or leisure activities)*

I have no problems doing my usual activities

I have slight problems doing my usual activities

I have moderate problems doing my usual activities

I have severe problems doing my usual activities

I am unable to do my usual activities

PAIN / DISCOMFORT

I have no pain or discomfort

I have slight pain or discomfort

I have moderate pain or discomfort

I have severe pain or discomfort

I have extreme pain or discomfort

ANXIETY / DEPRESSION

I am not anxious or depressed

I am slightly anxious or depressed

I am moderately anxious or depressed

I am severely anxious or depressed

I am extremely anxious or depressed

SAMPLE

- We would like to know how good or bad your health is **TODAY**.
- This scale is numbered from **0** to **100**.
- **100** means the <u>best</u> health you can imagine.
  0 means the <u>worst</u> health you can imagine.
- Mark an **X** on the scale to indicate how your health is **TODAY**.
- Now, please write the number you marked on the scale in the box below.

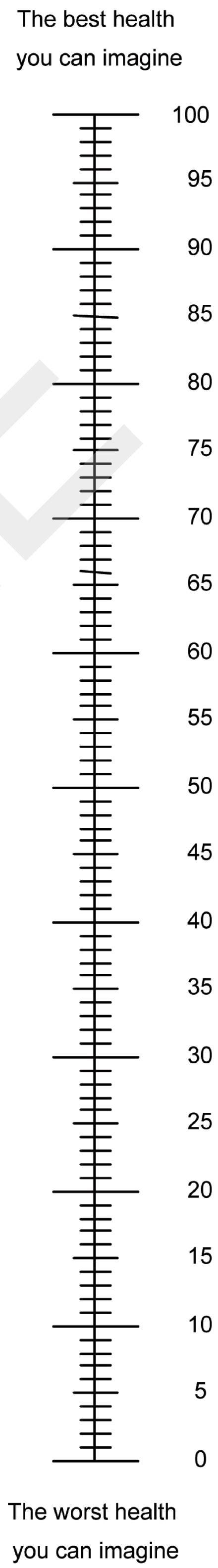

YOUR HEALTH TODAY =

SAMPLE

MELANOMA CONCERNS QUESTIONNAIRE [MCQ-28] Patient Version

Patients sometimes report that they have the following concerns after their diagnosis and treatment for melanoma. Please indicate the extent to which you have experienced these symptoms or concerns.

Please answer by ticking the option that best applies to you.

	**Have you had surgery for your melanoma in the last 12 months?**<br>**Yes** ☐ **No** ☐<br>**If yes, during the past 4 weeks**	**Not at all**	**A little**	**Quite a bit**	**Very much**
**1**	Have you had swelling near your melanoma site?	○	○	○	○
**2**	Have you had numbness at the site of your melanoma?	○	○	○	○
**3**	Have you had problems with pain at or near your melanoma site?	○	○	○	○
**4**	How much have you worried about complications due to your melanoma surgery?	○	○	○	○
**5**	How much have you worried about the length of time needed for your melanoma surgery to heal?	○	○	○	○

	**Since the diagnosis and treatment of your melanoma**	**Not at all**	**A little**	**Quite a bit**	**Very much**
**6**	Have you worried about the increased risk of melanoma for other members of your family?	○	○	○	○
**7**	Have you had any regrets about your exposure to sunshine in the past?	○	○	○	○
**8**	Have you felt concerned about conducting outdoor activities (e.g. working, swimming, walking, sport) which may result in exposure to the sun?	○	○	○	○
**9**	Have you considered making any significant changes to how you will live your life in the future?	○	○	○	○
**10**	Have you worried whilst waiting for results of medical tests?	○	○	○	○
**11**	Have you felt confused by information about your diagnosis or treatment from different sources (e.g. internet)?	○	○	○	○

	Since the diagnosis and treatment of your melanoma	Not at all	A little	Quite a bit	Very much
12	Have you felt hopeful for the future?	○	○	○	○
13	Have you felt able to face the challenges ahead?	○	○	○	○
14	Have you felt able to cope with your diagnosis of melanoma?	○	○	○	○
15	Have you felt able to deal with the shock of being diagnosed with melanoma?	○	○	○	○
16	Have you felt able to carry on with things as normal?	○	○	○	○
17	Have you felt able to feel positive?	○	○	○	○

	During the past 4 weeks		Not at all	A little	Quite a bit	Very much
18	If you work, have you had support from your employer to continue working during your treatment (e.g. flexible/reduced hours)	N/A	○	○	○	○
19	How much has your melanoma doctor supported you?		○	○	○	○
20	How much have your other healthcare professionals (e.g. nurse) supported you?		○	○	○	○
21	How much have your family members supported you?		○	○	○	○
22	How much has your primary care doctor supported you?		○	○	○	○
23	Have you felt able to contact the melanoma clinical staff if you needed to?		○	○	○	○
24	Have you felt confident that a psychological support service would be available if you needed it?		○	○	○	○
25	Have you been given enough time to think about the treatment options available to you?		○	○	○	○
26	Have you felt comfortable being intimate with your partner?	N/A	○	○	○	○
27	How much have those important to you been included in discussions about your treatment options?		○	○	○	○
28	Have you felt confident that your healthcare team communicate with you in a professional manner?		○	○	○	○

Depression, Anxiety and Stress Scale (DASS21)

For each statement below, please circle the number in the column that best represents how you have been feeling in the last week.

Statement	Did not apply to me at all	Applied to me to some degree or some of the time	Applied to me a considerable degree or a good part of the time	Applied to me very much or most of the time
1. I found it hard to wind down	0	1	2	3
2. I was aware of dryness of my mouth	0	1	2	3
3. I couldn't seem to experience any positive feeling at all	0	1	2	3
4. I experienced breathing difficulty (eg, excessively rapid breathing, breathlessness in the absence of physical exertion)	0	1	2	3
5. I found it difficult to work up the initiative to do things	0	1	2	3
6. I tended to over-react to situations	0	1	2	3
7. I experienced trembling (eg, in the hands)	0	1	2	3
8. I felt that I was using a lot of nervous energy	0	1	2	3
9. I was worried about situations in which I might panic and make a fool of myself	0	1	2	3
10. I felt that I had nothing to look forward to	0	1	2	3
11. I found myself getting agitated	0	1	2	3
12. I found it difficult to relax	0	1	2	3
13. I felt down-hearted and blue	0	1	2	3
14. I was intolerant of anything that kept me from getting on with what I was doing	0	1	2	3
15. I felt I was close to panic	0	1	2	3
16. I was unable to become enthusiastic about anything.	0	1	2	3
17. I felt I wasn't worth much as a person	0	1	2	3
18. I felt that I was rather touchy	0	1	2	3
19. I was aware of the action of my heart in the absence of physical exertion (eg, sense of heart rate increase, heart missing a beat)	0	1	2	3
20. I felt scared without any good reason.	0	1	2	3
21. I felt that life was meaningless	0	1	2	3

Lovibond, S.H. & Lovibond, P.F. (1995). Manual for the Depression Anxiety Stress Scales. (2nd. Ed.) Sydney: Psychology Foundation

ENGLISH

EORTC QLQ-C30 (version 3)

We are interested in some things about you and your health. Please answer all of the questions yourself by circling the number that best applies to you. There are no "right" or "wrong" answers. The information that you provide will remain strictly confidential.

Please fill in your initials:

Your birthdate (Day, Month, Year):

Today's date (Day, Month, Year): 31

	Not at All	A Little	Quite a Bit	Very Much
1. Do you have any trouble doing strenuous activities, like carrying a heavy shopping bag or a suitcase?	1	2	3	4
2. Do you have any trouble taking a long walk?	1	2	3	4
3. Do you have any trouble taking a short walk outside of the house?	1	2	3	4
4. Do you need to stay in bed or a chair during the day?	1	2	3	4
5. Do you need help with eating, dressing, washing yourself or using the toilet?	1	2	3	4

During the past week:

	Not at All	A Little	Quite a Bit	Very Much
6. Were you limited in doing either your work or other daily activities?	1	2	3	4
7. Were you limited in pursuing your hobbies or other leisure time activities?	1	2	3	4
8. Were you short of breath?	1	2	3	4
9. Have you had pain?	1	2	3	4
10. Did you need to rest?	1	2	3	4
11. Have you had trouble sleeping?	1	2	3	4
12. Have you felt weak?	1	2	3	4
13. Have you lacked appetite?	1	2	3	4
14. Have you felt nauseated?	1	2	3	4
15. Have you vomited?	1	2	3	4
16. Have you been constipated?	1	2	3	4

Please go on to the next page

ENGLISH

During the past week:	Not at All	A Little	Quite a Bit	Very Much
17. Have you had diarrhea?	1	2	3	4
18. Were you tired?	1	2	3	4
19. Did pain interfere with your daily activities?	1	2	3	4
20. Have you had difficulty in concentrating on things, like reading a newspaper or watching television?	1	2	3	4
21. Did you feel tense?	1	2	3	4
22. Did you worry?	1	2	3	4
23. Did you feel irritable?	1	2	3	4
24. Did you feel depressed?	1	2	3	4
25. Have you had difficulty remembering things?	1	2	3	4
26. Has your physical condition or medical treatment interfered with your family life?	1	2	3	4
27. Has your physical condition or medical treatment interfered with your social activities?	1	2	3	4
28. Has your physical condition or medical treatment caused you financial difficulties?	1	2	3	4

For the following questions please circle the number between 1 and 7 that best applies to you

29. How would you rate your overall health during the past week?

1 2 3 4 5 6 7

Very poor Excellent

30. How would you rate your overall quality of life during the past week?

1 2 3 4 5 6 7

Very poor Excellent

© Copyright 1995 EORTC Quality of Life Group. All rights reserved. Version 3.0
