# Supplementary material for: Embedding electronic patient-reported outcome measures into routine care for patients with stage III MELanoma (ePROMs-MEL): protocol for a prospective, longitudinal, mixed-methods pilot study
Source: BMJ Open. 2022 Dec 20;12(12):e066852. doi: 10.1136/bmjopen-2022-066852 (PMC9772660; doi:10.1136/bmjopen-2022-066852)
Supplement: Supplementary data [file bmjopen-2022-066852supp002.pdf]

APPENDIX 2.

Data from the questionnaires completed by the patients are uploaded into a custom-designed digital platform which allows instant access to the results presented in a user-friendly print-out.

This page shows how the results are displayed when a patient has identified anxiety:

Melanoma Institute Australia

Patient Reported Outcome Measures

Full Clinician Report- ePROMS-MEL Study

Patient Name: XXXXX
Date: 14-09-2022
Time Point: Baseline
Study ID: M130

Patient has reported moderate or high distress and/or poor quality of life.

Distress Thermometer score: (7/10)
(A value ≥ 4 is significant)

Patient has identified these problems from the Patient Problems List:
Work/school
Nervousness
Worry

EQ-5D-5L questionnaire results:

MOBILITY: 1 - I have no problems in walking about

SELF-CARE: 1 - I have no problems washing or dressing myself

USUAL ACTIVITIES: 1 - I have no problems doing my usual activities

PAIN / DISCOMFORT: 1 - I have no pain or discomfort

ANXIETY / DEPRESSION: 2 - I am slightly anxious or depressed

VISUAL ANALOGUE SCALE (VAS) score: 70/100
Global health rating Range = 0 - 100
0 = worst health you can imagine
100 = best health you can imagine

DASS21 questionnaire results:

DEPRESSSION: Normal

ANXIETY: Moderate

STRESS: Normal

This page shows the form for the clinician to complete.

Clinician Report – ePROMs-MEL study

Melanoma Institute Australia

Patient Name: Mrs Pilot Patient
Date: 23/02/2022
Time point: Follow-up 3
Study ID: ___________

Clinician name: ___________________________

Patient identified as distressed

Distress Thermometer Score: 7/10

(≥4/10 is clinically significant)

Patient identified these problems:

- Housing
- Treatment decisions
- Sadness

Clinician record of referrals (Y/N)

Services	Clinician wants to refer	Service is available	Patient would accept referral	Patient referred directly	Patient referred via GP
Psychology					
Social work					
Physio					
OT					
Other (please specify)					
1.					
2.					

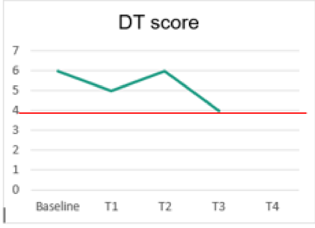

5

Full questionnaire data is uploaded from the iPad platform to a project REDCap database at the completion of recruitment and at the end of the study. This allows reports to be generated by the research team on aggregate data including trends over time, referrals and referral uptake. Reports on individual patients (e.g. to track changes in EORTC scores over time) can be generated at any time throughout the study.
