# Supplementary material for: Embedding electronic patient-reported outcome measures into routine care for patients with stage III MELanoma (ePROMs-MEL): protocol for a prospective, longitudinal, mixed-methods pilot study
Source: BMJ Open. 2022 Dec 20;12(12):e066852. doi: 10.1136/bmjopen-2022-066852 (PMC9772660; doi:10.1136/bmjopen-2022-066852)
Supplement: Supplementary data [file bmjopen-2022-066852supp003.pdf]

APPENDIX 3a **Completion survey - patient**

THE UNIVERSITY OF SYDNEY

NHMRC Clinical Trials Centre

Professor Rachael Morton
Director, Health Economics & Health Technology Assessment
Deputy Director, NHMRC Clinical Trials Centre

Camperdown, NSW 2050, Australia
Tel: 61-2-9562-5000
Fax: 61-2-9565-1863
Email: Rachael.morton@sydney.edu.au

Study ID number. ____________

Please circle/select the appropriate response.

1. Do you agree the questionnaires were easy to complete?

0	1	2	3	4
Strongly Disagree	Disagree	Neutral	Agree	Strongly Agree

Please provide reasons for your choice

____
____
____
____

2. Do you agree the questionnaires you completed measured the things that you consider important to your quality of life?

0	1	2	3	4
Strongly Disagree	Disagree	Neutral	Agree	Strongly Agree

Are there other questions you would have preferred to be asked in relation to your quality of life?

____
____
____
____

3. Do you agree your "whole of person" health and welfare has been improved because of the collection and consideration of the data on a real time basis with your clinician?

0	1	2	3	4
Strongly Disagree	Disagree	Neutral	Agree	Strongly Agree

Please provide reasons for your choice

____
____
____
____

4. Do you think your "whole of person" health and welfare has been improved because of the availability of support resources and groups? (0 = completely agree; 4 = completely disagree)

0	1	2	3	4
Strongly Disagree	Disagree	Neutral	Agree	Strongly Agree

Please provide reasons for your choice

____
____
____
____

5. Would you be willing to be contacted by the research team for a 10-20 minute confidential face to face or telephone interview to discuss your thoughts about the questions that you completed?

Yes No

If yes: Please provide your contact details below and a member of the research team will be in touch with you soon to discuss this further, and to provide you with a Participant Information Sheet and Consent Form for the interview.

Phone number: _________________________

Email address: ____

THANK YOU FOR COMPLETING THIS SURVEY.

Your time and cooperation is highly valued. We will keep you updated about the results.

The ePROMs-MEL team

.

APPENDIX 3b **Completion survey - clinician**

THE UNIVERSITY OF SYDNEY

NHMRC Clinical Trials Centre

Professor Rachael Morton
Director, Health Economics & Health Technology Assessment
Deputy Director, NHMRC Clinical Trials Centre

Camperdown, NSW 2050, Australia
Tel: 61-2-9562-5000
Fax: 61-2-9565-1863
Email: Rachael.morton@sydney.edu.au

Name: ____

Please circle/select the appropriate response

1. Age group: <30 31-40 41-50 51-60 >60

2. Gender: Male Female Other

3. How many years' experience do you have managing patients with stage III melanoma?

   < 1 year 1-5 years 6-10 years 11-15 years 16-20 years >20 years

4. What is your medical specialty?
   Surgeon
   Oncologist
   Nurse
   Specialist GP
   Allied Health (please specify) ____
   Other (please specify) ____

5. Currently, how many patients with stage III melanoma do you manage per year?

   <20 21-50 51-100 >100

6. Do you agree the results of the PROMs were useful?

0	1	2	3	4
Strongly Disagree	Disagree	Neutral	Agree	Strongly Agree

Please provide reasons for your choice
____
____
____
____

7. Do you agree the format of information provided to you was easy to read and understand?

0 Strongly Disagree	1 Disagree	2 Neutral	3 Agree	4 Strongly Agree

If not – how could this be improved?

____
____
____

8. Do you agree that the questionnaires were appropriate for your patients' Stage of disease?

0 Strongly Disagree	1 Disagree	2 Neutral	3 Agree	4 Strongly Agree

If not appropriate – why not?

____
____
____

9. Do you agree the questionnaires were adequately detailed to capture your patients' concerns related to quality of life or current health status?

0 Strongly Disagree	1 Disagree	2 Neutral	3 Agree	4 Strongly Agree

If not – what additional questions or topics would you like to be included in future sets of ePROMs / ePREMs?

____
____
____
____

10. Would you consider implementing ePROMs / ePREMs for other patients that you manage?

Yes No

If yes – for what other patient populations would you consider implementing ePROMs/ ePREMs? *For example, stage II, stage IV etc.*

____
____
____

If no – why would you not implement ePROMs/ ePREMs?

____
____
____

11. Overall, do you agree that incorporating ePROMs / ePREMs in routine care for patients with stage III melanoma is feasible?

0	1	2	3	4
Strongly Disagree	Disagree	Neutral	Agree	Strongly Agree

If agree or strongly agree: What factors make incorporating ePROMs / ePREMs in routine care for patients with stage III melanoma feasible?

____
____
____

If disagree or strongly disagree: Why do you believe that incorporating ePROMs / ePREMs in routine care for patients with stage III melanoma is not feasible?

____
____
____

12. Would you be willing to be contacted by the research team for a 10-20 minute confidential interview to discuss the feasibility and practicality of incorporating ePROMs/ ePREMs into routine care for patients with stage III melanoma?

Yes No

If yes: Please provide your contact details below and a member of the research team will be in touch with you soon to discuss this further, and to provide you with a Participant Information Sheet and Consent Form for the interview.

Phone number:______________________

Email address:____

THANK YOU FOR PARTICIPATING IN THIS STUDY – we know how busy you are!
Your time and cooperation is highly valued. We will keep you updated about the results.

The ePROMs-MEL team.

APPENDIX 3c **Completion survey - clinic staff**

THE UNIVERSITY OF SYDNEY

NHMRC Clinical Trials Centre

Professor Rachael Morton
Director, Health Economics & Health Technology Assessment
Deputy Director, NHMRC Clinical Trials Centre

Camperdown, NSW 2050, Australia
Tel: 61-2-9562-5000
Fax: 61-2-9565-1863
Email: Rachael.morton@sydney.edu.au

Please circle/select the appropriate response

1. Which clinic do you work in? (you may select more than one)

   SMSO Poche

   SMSO RPAH

2. Did you find the ePROMs project disruptive to clinic processes?

0	1	2	3	4
Strongly Disagree	Disagree	Neutral	Agree	Strongly Agree

If yes, in what ways?

____
____
____
____

3. Do you think the patients benefited from the project?

0	1	2	3	4
Strongly Disagree	Disagree	Neutral	Agree	Strongly Agree

If yes, in what ways?

____
____
____

4. Would you be pleased if ePROMs became part of routine care in your clinic?

0	1	2	3	4
Strongly Disagree	Disagree	Neutral	Agree	Strongly Agree

If no, why not?

____
____
____

5. Can you suggest changes that could be made to the project that would minimise clinic disruption?

Yes No

If yes, please share your suggestions below.

____
____
____
____

6. Would you consider implementing ePROMs for all patients in your clinic if additional resources were available?

Yes No

If yes, what additional resources would you want?

____
____
____

7. Would you be willing to be contacted by the research team for a 10-20 minute confidential interview to discuss the feasibility and practicality of incorporating ePROMs/ ePREMs into routine care for patients with stage III melanoma?

Yes No

If yes: Please provide your contact details below and a member of the research team will be in touch with you soon to discuss this further, and to provide you with a Participant Information Sheet and Consent Form for the interview.

Phone number:_____________________

Email address:____

THANK YOU FOR PARTICIPATING IN THIS STUDY – we know how busy you are!
Your time and cooperation is highly valued. We will keep you updated about the results.
The ePROMs-MEL team.
