# Supplementary material for: Embedding electronic patient-reported outcome measures into routine care for patients with stage III MELanoma (ePROMs-MEL): protocol for a prospective, longitudinal, mixed-methods pilot study
Source: BMJ Open. 2022 Dec 20;12(12):e066852. doi: 10.1136/bmjopen-2022-066852 (PMC9772660; doi:10.1136/bmjopen-2022-066852)
Supplement: Supplementary data [file bmjopen-2022-066852supp004.pdf]

APPENDIX 4. ePROMs-MEL Investigators

Professor Rachael Morton*<br>(Chief Investigator)<br>Rachael.Morton@ctc.usyd.edu.au	Director of Health Economics, NHMRC Clinical Trials Centre, The University of Sydney NSW Australia & Melanoma Institute Australia (MIA) Faculty
Associate Professor Robyn Saw*<br>Robyn.Saw@melanoma.org.au	Melanoma Surgeon, MIA Faculty, Royal Prince Alfred Hospital (RPAH), and Mater Hospitals & University of Sydney
Associate Professor Sergine Lo*<br>Serigne.Lo@melanoma.org.au	Senior Statistician, MIA Faculty and Senior Research Fellow in Biostatistics, The University of Sydney
Dr Iris Bartula*<br>Iris.Bartula@melanoma.org.au	Research and Clinical Psychologist, MIA
Dr Kathy Dempsey*<br>Kathy.Dempsey@sydney.edu.au	Senior Research Fellow, Cancer Health Policy. NHMRC Clinical Trials Centre, University of Sydney
Mr Craig Lawn*	Consumer Representative
Dr Thomas Pennington<br>Thomas.Pennington@melanoma.org.au	Melanoma Surgeon, MIA
Professor Andrew Spillane<br>Andrew.Spillane@sydney.edu.au	Melanoma and Breast Cancer Surgeon, MIA; Professor of Surgical Oncology, Royal North Shore Hospital & University of Sydney.
Professor Georgina Long<br>Georgina.Long@melanoma.org.au	Medical Oncologist, MIA; Co-Director, MIA
Assoc Professor Alex Menzies<br>Alex.Menzies@melanoma.org.au	Medical Oncologist, MIA
Professor Frances Boyle<br>Frances.Boyle@sydney.edu.au	Medical Oncologist, MIA; Director, Patricia Ritchie Centre for Cancer Care and Research, Mater Hospital, North Sydney; Professor of Medical Oncology, University of Sydney
Dr Mbathio Dieng<br>Mbathio.Dieng@sydney.edu.au	Postdoctoral Fellow, NHMRC Clinical Trials Centre, University of Sydney
Dr Skye Dong<br>Skye.Dong@melanoma.org.au	Clinical Psychologist, MIA
Mr Samuel Herzog<br>Samuel.Herzog@melanoma.org.au	Project Officer, ePROMs-MEL, MIA
Ms Shahn Coburn<br>Shahn.Coburn@melanoma.org.au	Clinical Nurse Consultant, MIA
Ms Kate Willis<br>Kate.Willis@melanoma.org.au	Clinical Nurse Consultant, MIA
Ms Rebecca Biviano<br>Rebecca.Biviano@melanoma.org.au	Clinical Nurse Consultant, MIA
Ms Natalie Osborne<br>Natalie.Osborne@melanoma.org.au	Clinical Nurse Consultant, RPAH
Ms Emel Fikri<br>Emel.Fikri@health.nsw.gov.au	Melanoma Nurse, RPAH
Dr Donna Milne<br>Donna.Milne@petermac.org	Melanoma Nurse, Peter MacCallum Cancer Centre
Mr Jake Thompson<br>Jake.Thompson@melanoma.org.au	Research Officer ePROMs-MEL, MIA
