# Supplementary material for: Embedding electronic patient-reported outcome measures into routine care for patients with stage III MELanoma (ePROMs-MEL): protocol for a prospective, longitudinal, mixed-methods pilot study
Source: BMJ Open. 2022 Dec 20;12(12):e066852. doi: 10.1136/bmjopen-2022-066852 (PMC9772660; doi:10.1136/bmjopen-2022-066852)
Supplement: Author's manuscript [file bmjopen-2022-066852.draft_revisions.pdf]

BMJ Open

BMJ Open is committed to open peer review. As part of this commitment we make the peer review history of every article we publish publicly available.

When an article is published we post the peer reviewers' comments and the authors' responses online. We also post the versions of the paper that were used during peer review. These are the versions that the peer review comments apply to.

The versions of the paper that follow are the versions that were submitted during the peer review process. They are not the versions of record or the final published versions. They should not be cited or distributed as the published version of this manuscript.

BMJ Open is an open access journal and the full, final, typeset and author-corrected version of record of the manuscript is available on our site with no access controls, subscription charges or pay-per-view fees (http://bmjopen.bmj.com).

If you have any questions on BMJ Open's open peer review process please email info.bmjopen@bmj.com

BMJ Open

Embedding electronic Patient Reported Outcome Measures into routine care for patients with Stage III MELanoma (ePROMs-MEL): Protocol of a prospective longitudinal Pilot study.

Journal:	*BMJ Open*
Manuscript ID	bmjopen-2022-066852
Article Type:	Protocol
Date Submitted by the Author:	26-Jul-2022
Complete List of Authors:	Dempsey, Kathy; The University of Sydney; Melanoma Institute Australia,<br>Saw, Robyn; Melanoma Institute Australia; The University of Sydney<br>Bartula, Iris; Melanoma Institute Australia<br>Lo, Serigne; Melanoma Institute Australia<br>Lawn, Craig; Melanoma Institute Australia<br>Pennington, Thomas; Melanoma Institute Australia,<br>Spillane, Andrew; Melanoma Institute Australia; The University of Sydney<br>Boyle, Frances ; Melanoma Institute Australia; The University of Sydney<br>Dieng, Mbathio; The University of Sydney<br>Dong, Skye; Melanoma Institute Australia,<br>Saks, Dina; Melanoma Institute Australia,<br>Lai-Kwon, Julia; Melanoma Institute Australia,<br>Thompson, Jake; Melanoma Institute Australia<br>Seaman, Linda; Melanoma Institute Australia<br>Milne, Donna; Peter MacCallum Cancer Centre<br>Morton, Rachael; The University of Sydney; Melanoma Institute Australia
Keywords:	Quality in health care < HEALTH SERVICES ADMINISTRATION & MANAGEMENT, MENTAL HEALTH, Dermatological tumours < ONCOLOGY, QUALITATIVE RESEARCH, Organisation of health services < HEALTH SERVICES ADMINISTRATION & MANAGEMENT

SCHOLARONE™
Manuscripts

Title

Embedding electronic Patient Reported Outcome Measures into routine care for patients with Stage III MELanoma (ePROMs-MEL): Protocol of a prospective longitudinal Pilot study.

Authors:

Dr Kathy Dempsey, BBSc, MA (Govt), PhD (Corresponding author)
Senior Research Fellow
NHMRC Clinical Trials Centre,
92-94 Parramatta Rd,
Camperdown NSW 2050.
The University of Sydney, NSW, Australia.
Phone: +61 2 9562 5014
Fax: +61 2 9565 1863
Email: kathy.dempsey@sydney.edu.au
ORCID ID: 0000-0003-4945-0857

Associate Professor Robyn PM Saw
Email: robyn.saw@melanoma.org.au
Melanoma Institute Australia
Wollstonecraft NSW 2065
Australia
ORCID ID: 0000-0002-3354-806X

Dr Iris Bartula
Email: iris.bartula@melanoma.org.au
Melanoma Institute Australia
Wollstonecraft NSW 2065
Australia
ORCID ID: 0000-0002-4084-4115

Associate Professor Serigne Lo
Email: serigne.lo@melanoma.org.au
Melanoma Institute Australia
Wollstonecraft NSW 2065
Australia
ORCID ID: 0000-0001-5092-5544

Mr Craig Lawn
Email: lawncorporate@bigpond.com
Melanoma Institute Australia
Wollstonecraft NSW 2065
Australia

Dr Thomas Pennington
Email: thomas.pennington@melanoma.org.au
Melanoma Institute Australia
Wollstonecraft NSW 2065
Australia

Professor Andrew Spillane

For peer review only

For peer review only - http://bmjopen.bmj.com/site/about/guidelines.xhtml

Email: andrew.spillane@sydney.edu.au
Melanoma Institute Australia
Wollstonecraft NSW 2065
Australia
ORCID ID: 0000-0002-8354-434X

Professor Frances Boyle
Email: frances.boyle@sydney.edu.au
Melanoma Institute Australia
Wollstonecraft NSW 2065
Australia
ORCID ID: 0000-0003-3798-1570

Dr Mbathio Dieng
Email: Mbathio.dieng@sydney.edu.au
NHMRC Clinical Trials Centre,
The University of Sydney, NSW, Australia
ORCID ID: 0000-0001-6714-9633

Dr Skye Dong
Email: skye.dong@melanoma.org.au
Melanoma Institute Australia
Wollstonecraft NSW 2065
Australia

Dr Dina Saks
Email: dinayaelsaks@gmail.com
Melanoma Institute Australia
Wollstonecraft NSW 2065
Australia

Dr Julia Lai-Kwon
Email: julia.lai-kwon@melanoma.org.au
Melanoma Institute Australia
Wollstonecraft NSW 2065
Australia
ORCID ID: 0000-0002-5388-1695

Mr Jake Thompson
Email: jake.thompson@melanoma.org.au
Melanoma Institute Australia
Wollstonecraft NSW 2065
Australia
ORCID ID: 0000-0002-0823-3218

Mrs Linda Seaman
Email: seaman.linda@gmail.com
Melanoma Institute Australia
Wollstonecraft NSW 2065
Australia

For peer review only

For peer review only - http://bmjopen.bmj.com/site/about/guidelines.xhtml

Mrs Donna Milne
Email: donna.milne@petermac.org.au
Peter MacCallum Cancer Centre
305 Grattan Street
Melbourne VIC 3000
Australia
ORCID ID: 0000-0002-9874-2023

Professor Rachael L. Morton
Email: Rachael.morton@sydney.edu.au
NHMRC Clinical Trials Centre,
The University of Sydney, NSW, Australia.
ORCID ID: 0000-0001-7834-0572

For peer review only

Key words

Quality in health care, mental health, dermatological tumours, qualitative health, organisation of health services

Word count

3,999 words

Abstract

Introduction. The benefits of patient-reported feedback, using questionnaires that allow patients to report how they feel and function without any interpretation from healthcare professionals, are well-established. However, Patient-Reported Outcomes Measures (PROMs) are not routinely collected as part of melanoma treatment in Australia. The primary aim is to evaluate the feasibility and acceptability of implementing electronic PROMs (ePROMs) into routine care from the perspectives of Stage III melanoma patients and their treating clinical team.

Methods and analysis. A minimum of 50 patients and 5 clinicians will be recruited to this prospective, longitudinal pilot study using a mixed methods approach (quantitative surveys and qualitative interviews) and conducted at surgical and medical melanoma clinics at two sites in metropolitan Sydney, Australia. A stakeholder reference panel, including experienced consumer representatives, ensured the views and preferences of patients were incorporated in planning and study design. All participants will complete the Distress Thermometer and EQ-5D-5L questionnaires using a tablet computer at baseline and a minimum of two subsequent appointments. Participants who report a high level of distress or poor quality of life will be triaged to complete an additional three questionnaires: the QLQ-C30, DASS and MCQ-28.

For peer review only - http://bmjopen.bmj.com/site/about/guidelines.xhtml

Results will be generated in real-time to enable discussion of patient-reported emotional well-being and quality of life. Patients with psychosocial distress or poor quality of life will recommended for referral to appropriate allied health services. Data analysis includes descriptive demographic data, questionnaire domain scores at each time point, completion, allied health referral and uptake rates. Thematic analysis of patient and clinician interviews will be conducted.

Ethics and dissemination. Original ethics approval was obtained from St Vincent's Hospital Human Research Ethics Committee (HREC) on 19/09/2019 (2019/ETH10558) with amendments approved on 8/06/2022. Dissemination will be wide-spread with a range of formats targeting different audiences.

Registration. Australian New Zealand Clinical Trials Registry. (anzctr.org.au/ACTRN12620001149954.aspx)

Strengths and limitations of this study

- This prospective, longitudinal cohort intervention pilot study, conducted at two sites, examines the perspectives of patients, clinicians and clinic staff.
- A stakeholder reference panel, with consumer representation, was formed in the planning stages of this study and was actively involved in study design.
- The study uses a triage system of distress screening to minimise survey burden for those who are not distressed and maximise information collection from those who are.
- Tablet computers (iPads) were chosen as the delivery platform because they are portable, have a user-friendly interface and provide a secure format for data management with their default encryption.
- Summary reports from the completed questionnaires, including the first use of the Melanoma Concerns Questionnaire-28 (MCQ-28), a bespoke melanoma PROMs questionnaire, are immediately forwarded to the treating clinician in real-time, so they can be discussed during the patient's consultation.

Introduction

Patient Reported Outcome Measures (PROMs) are questionnaires that allow patients to report how they feel and function without any interpretation from healthcare professionals.[1] PROMs can be used in the management of individual patients to screen for a particular condition (such as depression) and/or to monitor patient response to an appropriate intervention (such as psychological therapy).[1] Previous research has shown that the use of PROMs can improve clinician-patient communication. One study of women with gynaecological cancers found a PROMs

For peer review only - http://bmjopen.bmj.com/site/about/guidelines.xhtml

questionnaire helped with discussion of symptoms not typically addressed for nearly all participants (95%, n = 40) and improved patient care for 97% (n = 41) of participants.[2] Other studies have found PROMs can facilitate clinician awareness and improve patient management,[3,4] contribute to prognostic information,[3] potentially prolong survival outcomes in patients with advanced cancer,[5] as well as allowing patients to play a greater role in the clinical decision making process.[1] Currently, PROMs are not routinely collected as part of melanoma treatment in Australia.

It has long been recognised that many cancer specialists are not good at identifying cues for depression and psychological distress.[6-7] Routine screening for distress is now recommended by the United States National Comprehensive Cancer Network (NCCN)[8] and the International Psycho-Oncology Society and 68 affiliated organisations have set a standard of care involving monitoring distress as the '6th vital sign'.[9] Australian guidelines support the use of electronic PROMs (ePROMs) for the timely detection of psychosocial distress, but the authors note that results of trials evaluating the efficacy of distress screening have been mixed. Effective trials used strategies that minimised staff burden and ensured effective follow-up.[9]

Previous research has demonstrated increased anxiety, depression and lower quality of life in melanoma patients.[10-12] An Australian review of psychosocial support estimated 30% of all melanoma patients report levels of psychological distress indicative of the need for clinical intervention.[13] Stage III melanoma patients usually have more morbid surgery, including sentinel node biopsy or lymph node dissection, which impacts significantly on their quality of life. When patients with Stage III melanoma attend follow up appointments with their treating physician, the focus is primarily on detection and treatment of recurrence of disease. There is minimal focus on assessing the patients' quality of life or psychological health status, despite recommendations from The Cancer Institute NSW[14] and the Australian Commission on Safety and Quality in Health Care.[15] An audit of Australian cancer services reported that 'evidence from clinical settings suggests timely identification of distress is only effective in improving medical management and patient wellbeing when paired with structures supportive care referrals'.[16]

A 2022 report on melanoma in Australia noted the strong evidence that:

- *Access to emotional support services improves the well-being of people with cancer*
- *The opportunity to discuss feelings with a member of the treatment team or counsellor decreases psychosocial distress*
- *Psycho-educational programs decrease anxiety and depression.*

For peer review only - http://bmjopen.bmj.com/site/about/guidelines.xhtml

This landmark report concluded that increased screening for supportive care services should be developed as a quality indicator of melanoma clinical care.[17]

While there has been extensive research on estimating the extent of psychosocial distress in a vast range of clinical settings, subsequent translation of that knowledge into programs and services currently lags behind gains in the medical treatment of advanced melanoma.[18] This gap between obtaining results from PROMs and using this information as the first step in a process to actually improve patient outcomes is an overdue and urgent area of research. We believe there is an ethical imperative to fill this knowledge to implementation gap as soon as possible. The ePROMs-MEL project will address this urgent need.

By including the collection and evaluation of ePROMs data into the routine care for patients with Stage III melanoma, the treating clinician will be able to identify patients who are experiencing deterioration in quality of life or psychological distress. Once identified, these patients may then be referred to the appropriate allied health professionals (e.g. psychologist, specialist nurse, physiotherapist, occupational therapist, social worker) for appropriate support.

The primary aim of this pilot study is to evaluate the feasibility and acceptability of implementing ePROMs into routine care from the perspectives of both Stage III melanoma patients and their treating clinical team (i.e. surgeons, dermatologists, medical oncologists, psychologists and melanoma nurses). If the pilot results demonstrate technical, administrative and logistical feasibility, as well as patient, clinician and financial acceptability, this pilot will be expanded to all melanoma clinics within Melanoma Institute of Australia (MIA). This expansion will provide an opportunity to collect and analyse a greater volume of data and to potentially serve as a model for wider roll-out of ePROMs across the public and private healthcare sectors in NSW and other states and territories of Australia.

Methods

Patient and Public Involvement Statement

A stakeholder reference panel (SRP) was formed in the planning stages of this pilot study. Members of this panel included experts in the field of melanoma (various clinical and non-clinical specialties), experts in patient reported measures research, and a small group of representatives/advocates for patients with current and previously treated melanoma. The nominated consumer representative for this project is Mr Craig Lawn, who was assisted in the latter stages by Ms Linda Seaman.

For peer review only - http://bmjopen.bmj.com/site/about/guidelines.xhtml
For peer review only

Consumer involvement was considered vital to ensure that the needs and preferences of patients were incorporated alongside the knowledge and background of treating clinicians and researchers.

This SRP was responsible for drafting a detailed project proposal to be funded as part of the NHMRC Centre for Research Excellence in Melanoma. SRP tasks included: selection of the most appropriate PROMs to be included in this study, with consideration of minimising patient burden while maximising data usefulness; the content of semi-structured qualitative interview schedules (for patients and clinicians); development of all patient information documents, including the Participant Information Statement and Consent Forms, an Expression of Interest form and the "About you" demographic patient information collection form; clinician education resources including the development of short videos about the project and advice on discussion structure, in both electronic and written formats.

In addition to the documents, the manner of recruitment was widely discussed at these meetings, as was the creation of a comprehensive database that would receive results directly from the i-Pad custom-designed software while ensuring patient confidentiality. Dissemination of results to patients, clinicians and their general practitioners was strongly influenced by consumer views. A publication plan was prepared for dissemination of overall results to wider audiences.

Study design and setting

Prospective, longitudinal intervention study incorporating mixed methods (quantitative surveys and qualitative interviews). This pilot study is based on a single cohort of patients over an 18-month period (including periods of inactivity due to COVID-19). Although there is no control group, the design incorporates before-and-after study design elements allowing for comparisons of psychosocial health and quality of life in individuals with Stage III melanoma before and after the introduction of ePROMs. This study will be conducted at surgical and medical melanoma clinics at MIA and at Royal Prince Alfred Hospital (RPAH), Sydney, NSW, Australia.

Eligibility criteria and sample size

Patients are eligible to participate if they are aged 18 or over, have received a diagnosis of Stage III melanoma at least one month earlier, are under the care of melanoma clinicians at MIA or RPAH, and have sufficient English and cognitive ability to comprehend study materials, provide informed consent and participate in the study. Clinicians, including surgical oncologists, medical oncologists,

For peer review only

For peer review only - http://bmjopen.bmj.com/site/about/guidelines.xhtml

dermatologists and melanoma general practitioners, are eligible to participate if they are currently treating and managing patients with stage III melanoma at MIA or RPAH.

The sample size for this pilot feasibility study is 50 patients and approximately five clinicians. It is estimated that only 5% of all Stage III melanoma patients currently receive referrals for psychological care (MIA, data unpublished). Assuming 30% of Stage III melanoma patients have levels of distress that indicate the need for clinical intervention,[13] we would expect to identify approximately 15 patients within our sample who will be triaged for discussion and/or referrals to allied health support services.

Recruitment

Recruitment has been intermittent due to delays caused by COVID-19. Recruitment was extended to medical oncology clinics at MIA in March 2022 to meet the recruitment target.

Patient recruitment

A minimum of 50 patients will be recruited through surgical and medical melanoma clinics at MIA and RPAH. A study clinician will review a list of potentially eligible patients (with Stage III melanoma) obtained from the MIA Melanoma Research Database to check clinical eligibility. Updated lists will then be sent to each of the treating clinicians who will be able to remove patients they feel are not appropriate to invite to participate in the study for other reasons. The clinical nurse consultant will then send the approved list of eligible patients to the project officer.

These patients will be initially contacted by the project officer prior to their scheduled clinical review appointments. The project officer will send them three documents along with a covering letter (either by email or post; posted documents will contain a stamped self-addressed envelope). The email/letter will briefly introduce the study, ask patients to read the Invitation to Participate and the Participant Information Statement, and to complete and return an Expression of Interest (EoI) form. The EoI form provides patients with three options to select from. The first option denotes their willingness to participate in the study; the second denotes their potential willingness to participate in the study; and the third option states that they are not interested in participating. If patients do not respond to the request to return the EoI, the project officer will telephone them once to check whether they have received it.

The project officer will follow up those patients who have indicated their willingness or potential willingness to participate, answer any questions and concerns they may have about the study and

For peer review only - http://bmjopen.bmj.com/site/about/guidelines.xhtml

For peer review only

ask them to arrive 30 minutes earlier for their scheduled appointment to allow time to complete the questionnaires. The PIS contains contact numbers of the academic (RLM) and clinical leads (RPMS) for this project so patients may contact them prior to making their decision to participate. When patients arrive at the clinic for a regular appointment, they will again be given the opportunity to discuss any questions they may have regarding their participation with the clinical nurse consultant who will greet them, obtain their electronic consent on the iPad and explain to them how to complete the questionnaires on the iPad.

Clinician recruitment

Clinical staff will be informed about the project through education sessions at clinical and multi-disciplinary team (MDT) meetings and be given a written Invitation to Participate. Interested clinicians will be provided with an electronic Participant Information Sheet and Consent Form (PISCF) to read. Either hard copies of signed consent or scanned electronic versions will be acceptable and collated by the project officer prior to the clinician seeing study patients.

Interventions

The study questionnaires will be repeated at subsequent regularly scheduled follow-up appointments with their clinicians, with a minimum of two months between patient completion of questionnaires. iPads were chosen as the delivery platform because they are portable, have a user-friendly interface, are capable of hosting software that can automatically generate reports and associated documents and are a secure format for data management as they are encrypted by default. All questionnaires used in this study have been validated in previous studies. The MCQ-28 is a recently-developed questionnaire which includes some patient-reported experience measures (PREMs) in addition to PROMs.[19] The remaining four questionnaires cover PROMs only.

At each visit, all patients will be required to complete two short questionnaires that are used to assess their psychosocial distress and quality of life: 1. the National Comprehensive Cancer Network (NCCN)'s Distress Thermometer (DT) and Problem List for Patients;[20] and 2. EuroQoL's EQ-5D-5L and visual analogue scale.[21] Patients who score above the clinical cut off score on the DT (4 or above) will be triaged to complete an additional three questionnaires: the MCQ-28,[19] the Depression, Anxiety and Stress Scale (DASS-21)[22] and the European Organisation for Research and Treatment of Cancer (EORTC QLQ-C30).[23] These additional questionnaires will provide more detailed information about potential areas of support needs. Completion time for all five questionnaires is expected to be less than 30 minutes.

For peer review only - http://bmjopen.bmj.com/site/about/guidelines.xhtml
For peer review only

Patients who were recruited with Stage III melanoma but progress to Stage IV disease during the study can choose whether to continue in the study or not. Any continuing Stage IV patients will be asked to complete an interview at the end of the study to provide an assessment of how helpful they found this project to be considering their changing treatment and needs.

Results from the completed questionnaires will be automatically generated and sent to the treating clinician in real-time, so the results can be discussed during their consultation, along with medical concerns. Patients who request a copy of their results (by selecting the appropriate box in the 'About You' survey) will receive a summary of their scores by email, prior to their imminent appointment. Participants who score 3 or above on any of the five domains covered in the EQ-5D-5L questionnaire (mobility, self-care, usual activities, pain/discomfort and anxiety/depression) may require clinical intervention (3= moderate, 4= severe, 5 = extreme limitations). There are no melanoma-specific cut-off scores indicating the need for clinical intervention, for the QoL questionnaires MCQ-28[19] and EORTC QLQ-C30.[23] For the initial appointment, these scores will be recorded as baseline measures. At subsequent visits, changes in the scores for all questionnaires will be graphically represented by domain and included in the results for clinicians to examine trends. A deterioration in EORTC QLQ-C30 scores that is 10 points or greater between visits is commonly regarded as clinically significant[24,25] and will represent a further trigger for discussion and possible referral to an appropriate service/support. Recommendations and referrals for additional support services will be based on the questionnaire scores and clinical experience of the clinicians and be made in collaboration with the patient.

After all rounds of patient questionnaires have been completed, patients, clinicians and clinic staff will be asked to complete a survey providing quantitative and open-ended assessments of their experiences of using the PROMs questionnaires on iPads. In addition, approximately 10 patients, all study clinicians and some clinic staff will be asked whether they would be willing to be contacted by the research team to participate in a short (10-minute) confidential interview to expand on their survey responses. An additional Participant Information Sheet and Consent Form (PISCF) will be distributed to interested patients, clinicians and clinic staff and signed consent forms will be returned to the project officer prior to interviews being conducted. Patients will be purposively sampled to maximise response variation and those who complete an interview will be offered a $30 gift card to thank them for their time. Clinicians and clinic staff who participate in an interview will not be reimbursed for their time. Finally, patients will also be given the option of receiving feedback on the overall study results by selecting the appropriate box on the 'About You' survey. This

For peer review only - http://bmjopen.bmj.com/site/about/guidelines.xhtml

feedback will be in the form of a one-page lay summary, which participants will receive after the study has finished. Participating clinicians will receive feedback on study outcomes via seminars and MDT presentations. Figure 1 provides a study Flow Chart from the patient's perspective and Figure 2 provides the study overview from the clinician's perspective.

[FIGURE 1 HERE]

[FIGURE 2 HERE]

For peer review only

For peer review only - http://bmjopen.bmj.com/site/about/guidelines.xhtml

Outcomes

The primary intended outcome of this study is to determine whether completion of PROMs electronically are considered by both patients, clinicians and clinic staff as feasible and acceptable when incorporated into routine clinical care. Important secondary outcomes include an assessment of whether these measures can help identify people who need and would benefit from further support/referral; and the production of a framework for the data systems required to enable electronic data capture and feedback to clinicians, useful in many other applications and clinical specialties. The primary outcome of patient and clinician perceptions of the feasibility and acceptability of the intervention has three components.

Patient perceptions

Patient ratings of feasibility and acceptability will be assessed in several ways. The first is by the proportion of eligible patients approached who completed baseline and at least one set of follow-up PROMs data. If 60% of patients meet this requirement, the study will be deemed feasible and acceptable overall.

Data on individual patient measures of feasibility and acceptability will also be collected. In the first set of two triage questionnaires, all questions are mandatory, but with the second set of three questionnaires, patients are not required to answer all questions. Metadata from the patients who are triaged to complete the second group of questionnaires will be collected to provide an estimate of the mean time taken to complete the PROMs questionnaires and the amount of missing data (providing a second measure of patient acceptability for those who completed all five questionnaires). If patients take more than 30 minutes to complete the five questionnaires, they will be considered as not having met this acceptability criteria. Additional measures will include changes in baseline and follow-up scores and the proportion of patients who: ‘agree or strongly agree’ that the PROMs were easy to complete and that the questions they completed measured things they considered important to their QoL. Semi-structured interviews with patients will also assess feasibility and acceptability through the following main question themes: enjoyment of completing the PROMs; changes in patient-clinician communication; changes in their management; and improvements or challenges they found in the processes.

Clinician perceptions

It is also important to assess the feasibility and acceptability of this project from the clinicians’ perspective. These include whether they found the PROMs data provided to them to be useful, timely, easy to understand and appropriate for their patient population. Clinicians will be approached to expand on their survey responses in a semi-structured interview. The key themes to address are related specifically to the PROMs instruments and the information generated for the clinician; changes in patient management; improvements in patient-clinician communication; and any key challenges or improvements that they can identify regarding the processes.

For peer review only - http://bmjopen.bmj.com/site/about/guidelines.xhtml

Clinic staff perceptions

Clinic staff including clinical nurse consultants and medical secretaries will also be asked to assess the feasibility and acceptability of incorporating QoL questionnaires into their clinic's routine practice. If they consider the additional workload or clinic disruption to be unacceptable or burdensome, then this will have a major impact on the feasibility assessment of continuing this practice once the pilot study has been completed.

Secondary outcomes

Research and clinical psychologists at MIA will use identified unmet needs from this study to inform future research into the development of support programs at MIA. The extent to which the routine use of PROMs in clinical practice can improve patients' emotional and physical problems will be assessed through changes in referral rates, referral uptake rates and patients' QoL scores over time. Patient and clinician interviews will allow for more extensive evaluation of this aspect of the study.

The utility of this framework for the data systems required to enable electronic data capture and feedback to clinicians will be assessed in patient, clinician and clinic staff surveys and interviews as well as assessment by the project team. End of study surveys will highlight the perceived ease of use, usefulness, relevance and importance of electronic PROMs to each group. Data will also be collected on time taken to complete questionnaires and on the number of times the questionnaires were completed (out of a maximum of five times). In addition, the number of questions answered in the second set of three questionnaires (all questions in the first set of two questionnaires are mandatory) will be recorded.

All participating clinicians are experienced in treating Stage III melanoma patients and will be asked to review resources prepared for this study. These include three short videos about PROMs, referral pathways and the project details, and support services referral information on the MIA intranet. The aim of these resources is to help clinicians familiarise themselves with the objectives of the study and the use of PROMs in an oncology setting. Table 1 provides a timeline of expected outcomes.

For peer review only

For peer review only - http://bmjopen.bmj.com/site/about/guidelines.xhtml

Table 1. Study Interventions and Assessments Timeline

	Baseline			End of study
**TIME POINTS**[1]	T0	T1	T2	T3
ENROLMENT				
Eligibility screen	X			
Informed consent	X			
Recruitment	X			
INTERVENTIONS				
Baseline patient questionnaires	X			
Baseline patient demographic info	X			
Follow-up patient questionnaires		X	X	X
Clinician completion surveys				X
Patient completion surveys				X
Clinician completion interviews				X
Patient completion interviews				X
Clinician referral data				X
Patient referral uptake data				X
Patient change over time (T0-T5)				X
ASSESSMENTS				
Clinician acceptance				X
Patient acceptance				X
Clinic staff acceptance				X
Referral rate				X
Referral uptake rate				X

Table 1 adapted from the template designed by the SPIRIT Group ©2013[27]
1. For each time point, a window of +/- 2 months is acceptable due to variation in routine clinical appointments.

The overall success of the project will be assessed using the Proctor et al. framework for implementation research[28] which considers not only the intervention outcomes (service and client) but also the implementation outcomes, as outlined in Fig. 3.

[FIGURE 3 HERE]

Data analysis / Statistical methods

Quantitative analysis

Participant demographics and characteristics will be summarised for all participants using means and standard deviations. In addition, the consent, participation, and completion rates will be analysed. Consent will be defined as agreeing to participate in the study and returning a signed consent form; participation, as completing some of

For peer review only - http://bmjopen.bmj.com/site/about/guidelines.xhtml

the components of the study; and completion, as completing all components of the study. Specifically, the proportion of eligible participants who completed the project tasks will be assessed to determine whether the inclusion of ePROMs are acceptable for patients undergoing follow-up treatment for stage III melanoma. Descriptive statistics will be reported for QoL and other questionnaire domain scores at baseline and follow-up time points. Referral and referral uptake rates will also be calculated. The small sample size of 50 for this pilot study precludes any sub-group analysis.

Qualitative analysis

Results from semi-structured patient interviews conducted at the conclusion of the project will be reported to assess patient views on the usability, benefits and limitations of the study questionnaires they completed, whether questionnaire results were discussed within the subsequent clinical consultation, and if so whether this led to any change in management. Similarly, results from end of study clinician interviews will report their views on the feasibility, acceptability, benefits, limitations and usefulness of including ePROMs into the routine care for patients within their practice. Electronic qualitative analysis software (such as NVivo) will be used to assist in the development of a framework of key themes identified from the interview data that will then form the basis of a thematic analysis.[26]

Ethics approval

Original ethics approval was obtained from St Vincent's Hospital Human Research Ethics Committee on 19/09/2019 (2019/ETH10558). This approval included site authorisation for the following two project sites:

- Sydney Melanoma & Surgical Oncology, MIA, Poche Centre
- Sydney Melanoma & Surgical Oncology, Royal Prince Alfred Hospital (RPAH) Camperdown

Amendments to the original protocol were accepted by St Vincent's HREC on 8 June 2022.

There are no foreseeable harms, risks or costs associated with taking part in this study. It is possible some patients may experience short-term distress while answering the questionnaires. If a patient does not wish to answer a question, they may skip it and go to the next question, or immediately stop completing the questionnaires. A melanoma clinical nurse consultant and/or treating clinician will be available to talk with a distressed patient if their distress continues once they have stopped completing the questionnaire. Patients are free to withdraw from the study at any time. Clinicians may experience frustration with having to review patient summary scores from the questionnaires if they are particularly busy.

Dissemination policy

For peer review only

For peer review only - http://bmjopen.bmj.com/site/about/guidelines.xhtml

Communication of trial results

Study results will be communicated to participants via a short, lay summary of overall study findings at the end of the study. Patients can also opt in to receive their personal questionnaire results at each scheduled visit over the study time period. Overall study results will be communicated to local healthcare professionals through multidisciplinary team meetings and to wider clinician audiences through presentations to the national surgical oncology, medical oncology and nursing representative societies. In addition to the standard fora of publication in peer-reviewed journals, seminar/webinar presentations and presentations at scientific conferences, presentations and summary materials targeting consumers and their families will be developed.

Public access to data

De-identified data only will be made available through a nominated data repository on request. Public access to participant-level data will not be available due to patient privacy concerns.

Publications policy

A publications policy has been created for this study. It outlines processes for authorship and will guide the appropriate recognition of all contributors to the intellectual property developed within the study. It applies to all publications that present research arising from and/or receiving funding from the pilot study and main study.

Authors' contributions to the protocol

The ePROMs-MEL Steering Committee (Rachael Morton (RLM), Robyn Saw (RPMS), Iris Bartula (IB), Serigne Lo (SL), Craig Lawn (CL) and Kathy Dempsey (KD)) has been responsible for the development of this protocol. A full list of Investigators, including Steering Committee members, is available in Appendix 1. RLM is a Chief Investigator on the Melanoma CRE and conceived of the study. RLM worked with RPMS and IB on the study design. SL is the project biostatistician and consumer representative Mr Craig Lawn was substantially involved in the development of this protocol. KD was responsible for protocol revisions.

Competing interest statements

RPMS has received honoraria for advisory board participation from Merck Sharp & Dohme (MSD), Novartis and Qbiotics and speaking honoraria from Bristol Myers Squibb (BMS).

Funding statement

KD's salary is supported through the Australian National Health and Medical Research Council (NHMRC) Centre for Research Excellence in Melanoma grant (Grant No.1135285). RPMS is supported by MIA. The project officer's salary is supported by a grant from The University of Sydney's DVCR Support Fund for COVID-19 impacted research. Support is also provided by Melanoma Institute Australia for IT programming and educational design,

For peer review only

For peer review only - http://bmjopen.bmj.com/site/about/guidelines.xhtml

as well as access to the Melanoma Research Database to aid in the selection of eligible patients. The study funder has no role in, or influence on, the study design; collection, management, analysis, and interpretation of data; writing of the report; and the decision to submit the report for publication.

Acknowledgements

We would like to acknowledge the significant contribution of other colleagues to the development of this protocol:

Information technology staff: Ivy Tan and Ernie Wise

Former project officers: Sam Robinson and Emma Zhang

For peer review only

References

1. Bantug ET, Coles T, Smith KC, Snyder CF, Rouette J, Brundage MD. Graphical displays of patient-reported outcomes (PRO) for use in clinical practice: What makes a pro picture worth a thousand words? Patient Educ Couns. 2016;99: 483-490.

2. Webster EM, Burke WM, Ware HM, Jones BA, Vattakalam R, Tergas AI, Wright JD, Hou JY. Patient reported outcomes in evaluation of chemotherapy toxicity in women with gynecologic malignancies: A pilot study. Gynecol Oncol 2018;150:487-493.

3. Bouazza YB, Chiairi I, El Kharbouchi O, De Backer L, Vanhoutte G, Janssens A et al. Patient-reported outcome measures (PROMs) in the management of lung cancer: A systematic review. Lung Cancer 2017;113:140-151.

4. Kargo AS, Coulter A, Jensen PT, Steffensen KD. Proactive use of PROMs in ovarian cancer survivors: a systematic review. J Ovarian Res 2019;12:63.

5. Basch E, Deal AM, Dueck AC, Scher HI, Kris MG, Hudis C, Schrag D. Overall survival results of a trial assessing patient-reported outcomes for symptom monitoring during routine Cancer treatment. JAMA. 2017;318(2):197–198.

6. Passik SD, Dugan W, McDonald MV, Rosenfeld B, Theobald DE, Edgerton S. Oncologists' recognition of depression in their patients with cancer. J Clin Oncol 1998; 16:1594-1600.

7. Fallowfield L, Ratcliffe D, Jenkins V, Saul J. Psychiatric morbidity and its recognition by doctors in patients with cancer. Br J Cancer 2001; 84(8): 1011–1015.

For peer review only - http://bmjopen.bmj.com/site/about/guidelines.xhtml

8. National Comprehensive Cancer Network (NCCN). NCCN Clinical Practice Guidelines in Oncology. Distress Management. V2.2020. March 11 2020. NCCN.org [restricted access]

9. Butow PN, Price MA, Shaw JM, Turner J, Clayton JM, Grimison P, Rankin N. [on behalf of the Psycho-Oncology Co-operative Research Group (PoCoG)]. Clinical pathway for the screening, assessment and management of anxiety and depression in adult cancer patients: Australian guidelines. Psycho-oncology 2015;24(9):987-1001.

10. Dieng M, Butow P, Costa D, Morton R, Menzies S, Mireskandari S et al. Psychoeducational intervention to reduce fear of cancer recurrence in people at high risk of developing another primary melanoma: Results of a randomized controlled trial. J Clin Oncol 2016;34(36), 4405-4414.

11. Stamataki Z, Brunton L, Lorigan P, Green AC, Newton-Bishop J, Molassiotis A. Assessing the impact of diagnosis and the related supportive care needs in patients with cutaneous melanoma. Support Care Cancer 2015;23:779–789.

12. Fu H, Teleni L, Crichton M, Chan RJ. Supportive care and unmet needs in patients with melanoma. A mixed-methods systematic review. Support Care Cancer 2020. https://doi.org/10.1007/s00520-020-05464-3

13. Kasparian NA. Psychological stress and melanoma: Are we meeting our patients' psychological needs? Clin Dermatol 2013;31:41-46

14. Cancer Institute NSW. Patient-reported measures (PRMs). https://www.cancer.nsw.gov.au/patient-reported-measures [accessed 12/5/2020]

15. Australian Commission on Safety and Quality in Health Care. Evidence on the use of PROMs in clinical practice, clinical research, quality improvement and policy development.

16. Fradgley EA, Byrnes E, McCarter K, Rankin N, Britton B, Clover K, Carter G, Bellamy D, Paul CL. A cross-sectional audit of current practices and areas for improvement of distress screening and management in Australian cancer services: is there a will and a way to improve? Support Care Cancer. 2020;28: 249-259.

17. Insight Economics, Melanoma Institute Australia and Melanoma Patients Australia. State of the Nation. A Report into Melanoma – A National Health Priority. Final Report; Sydney: February 2022.

18. Dunn J, Watson M, Aitken JF, Hyde MK. Systematic review of psychosocial outcomes for patients with advanced melanoma. Psychooncology. 2017;26: 1722-1731.

For peer review only

For peer review only - http://bmjopen.bmj.com/site/about/guidelines.xhtml

19. Winstanley J, Boyle F, Young T. Melanoma Concerns Questionnaire© [MCQ-28] Version 1. 31 Jan 2020.

20. The National Comprehensive Cancer Network (NCCN®) NCCN Distress Thermometer and Problem List for Patients. Version 2.2018, 02/23/18.

21. Herdman M, Gudex C, Lloyd A et al. Development and preliminary testing of the new five-level version of EQ-5D (EQ-5D-5L). Quality of Life Research 2011;20(10):1727-1736.

22. Lovibond SH and Lovibond PF. (1995). Manual for the Depression Anxiety Stress Scales. (2nd. Ed.) Sydney: Psychology Foundation.

23. European Organisation for Research and Treatment of Cancer (EORTC) Quality of Life Group. Quality of Life Questionnaire-Cancer 30 (QLQ-C30). Version 3.0

24. Cocks K, King MT, Velikova G, Fayers PM, Brown JM. Quality, interpretation and presentation of European Organisation for Research and Treatment of Cancer quality of life questionnaire core 30 data in randomised controlled trials. Eur J Cancer. 2008;44: 1793-1798.

25. Musoro JM, Bottomley A, Coens C, Eggermont AM, King MT, Cocks, K et al. EORTC Melanoma Group and EORTC Quality of Life Group. Interpreting European Organisation for Research and Treatment for Cancer Quality of life Questionnaire core 30 scores as minimally importantly different for patients with malignant melanoma. Eur J Cancer 2018;104:169-181. doi: 10.1016/j.ejca.2018.09.005

26. Gale NK, Heath G, Cameron E, Rashid S, Redwood S. Using the framework method for the analysis of qualitative data in multi-disciplinary health research. BMC Medical Research Methodology 2013;13:117. doi:10.1186/1471-2288-13-117.

27. Calvert M, Kyte D, Mercieca-Bebber R, Slade A, Chan A-W, King MT; and the SPIRIT-PRO Group. Guidelines for Inclusion of Patient-Reported Outcomes in Clinical Trial Protocols: The SPIRIT-PRO Extension. JAMA. 2018;319(5):483-494.

28. Proctor E, Silmere H, Raghavan R, et al. Outcomes for implementation research: conceptual distinctions, measurement challenges, and research agenda. Administration and Policy in Mental Health and Mental Health Services Research. 2011;38(2):65-76.

Figure Legend

Figure 1. Patient's perspective

Figure 2. Clinician's perspective

Figure 3: Implementation Research Outcomes [ref 28]

For peer review only - http://bmjopen.bmj.com/site/about/guidelines.xhtml

For peer review only

For peer review only - http://bmjopen.bmj.com/site/about/guidelines.xhtml

ePROMS-MEL FLOW CHART

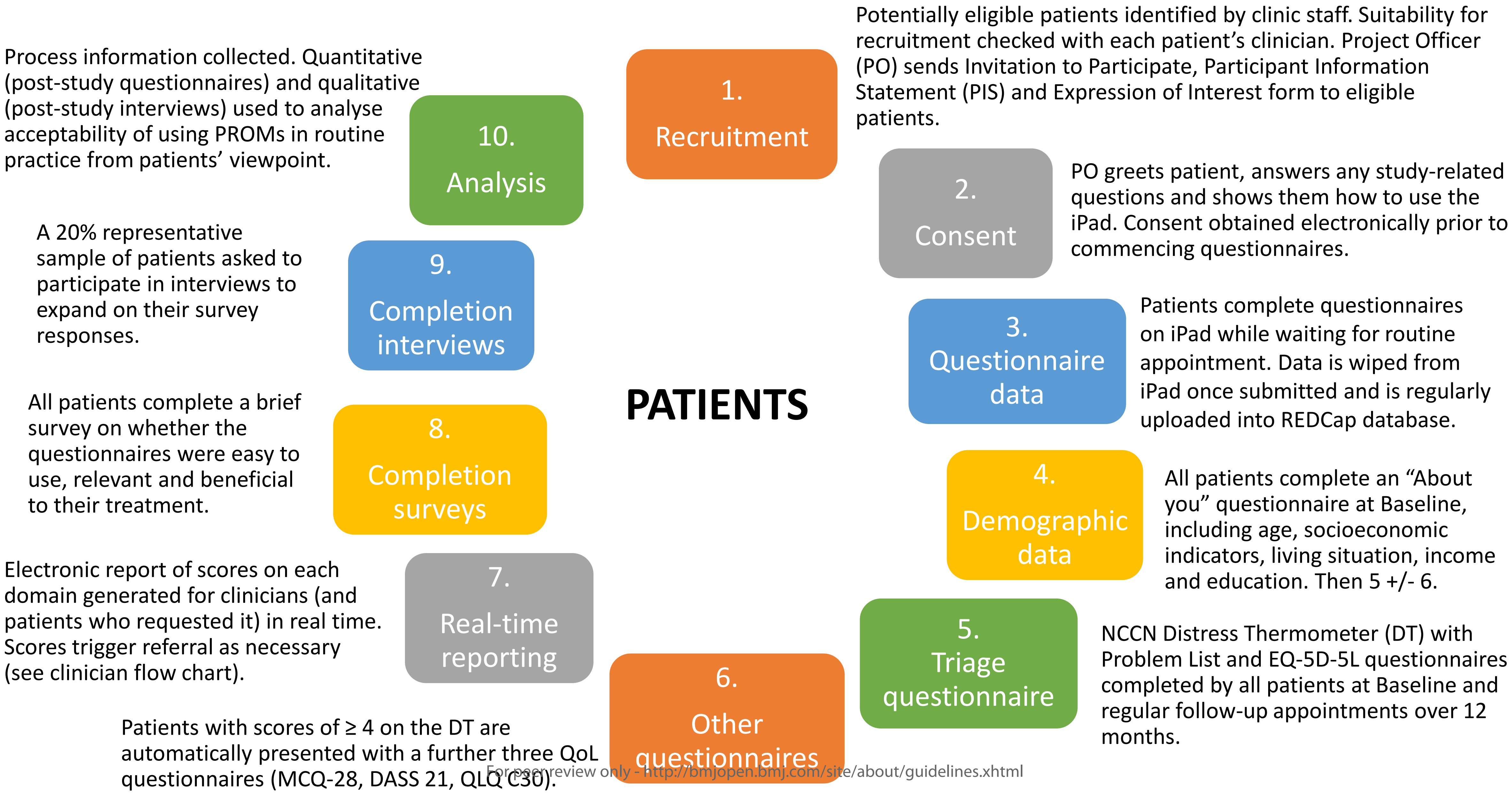

For peer review only - http://bmjopen.bmj.com/site/about/guidelines.xhtml

ePROMS-MEL FLOW CHART

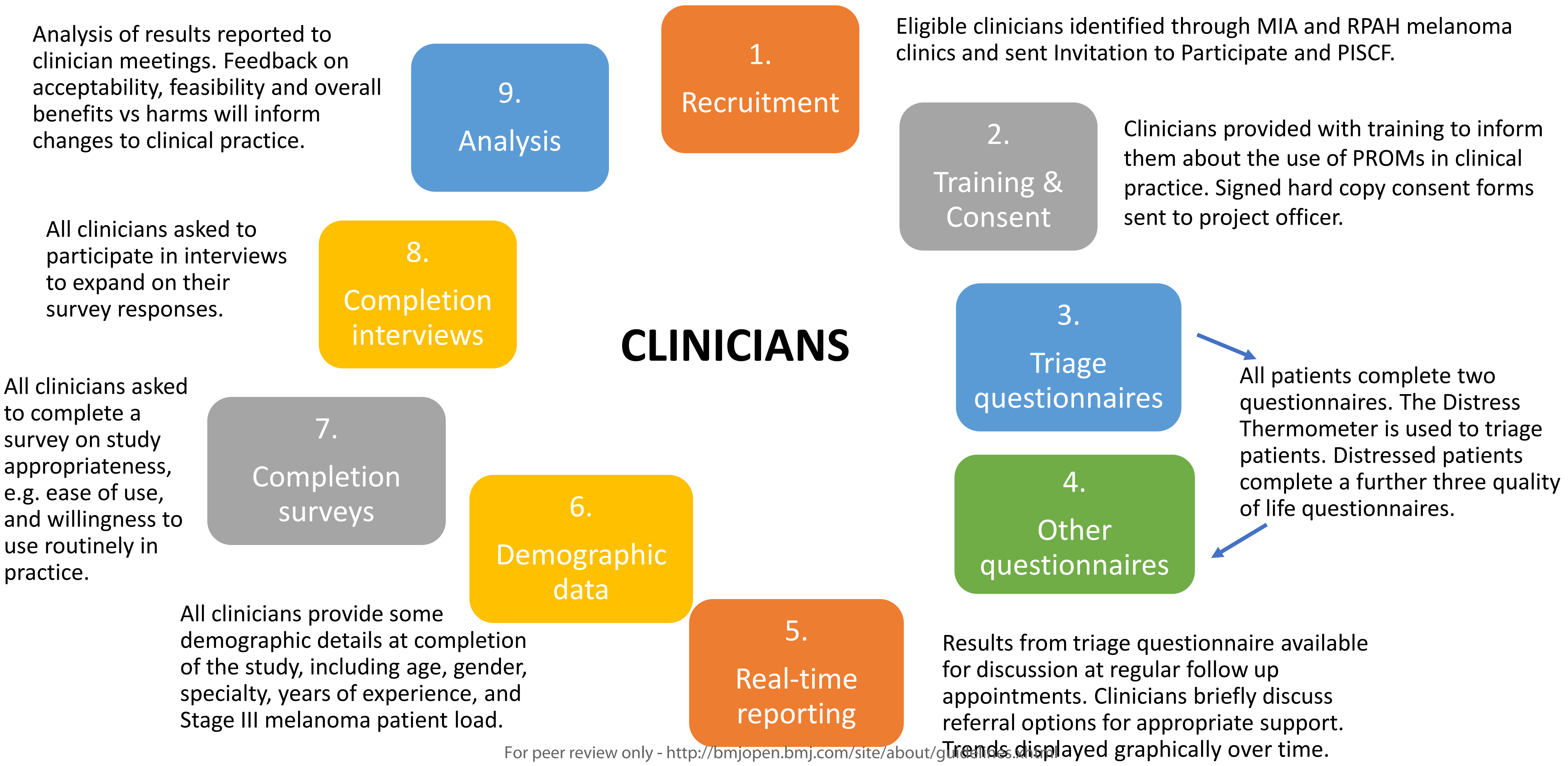

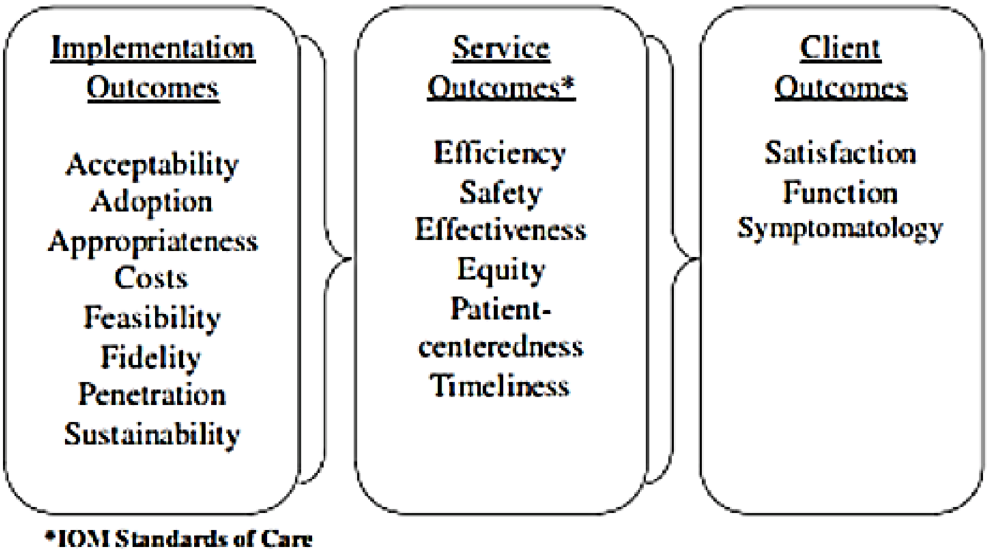
Implementation Outcomes
Acceptability
Adoption
Appropriateness
Costs
Feasibility
Fidelity
Penetration
Sustainability
Service Outcomes*
Efficiency
Safety
Effectiveness
Equity
Patient-centeredness
Timeliness
Client Outcomes
Satisfaction
Function
Symptomatology
*IOM Standards of Care

Appendix 1. ePROMs-MEL Investigators

Professor Rachael Morton* (Chief Investigator) Rachael.Morton@ctc.usyd.edu.au	Director of Health Economics, NHMRC Clinical Trials Centre, The University of Sydney NSW Australia & Melanoma Institute Australia (MIA) Faculty
Associate Professor Robyn Saw* Robyn.Saw@melanoma.org.au	Melanoma Surgeon, MIA Faculty, Royal Prince Alfred Hospital (RPAH), and Mater Hospitals & University of Sydney
Associate Professor Sergine Lo* Serigne.Lo@melanoma.org.au	Senior Statistician, MIA Faculty and Senior Research Fellow in Biostatistics, The University of Sydney
Dr Iris Bartula* Iris.Bartula@melanoma.org.au	Research and Clinical Psychologist, MIA
Dr Kathy Dempsey* Kathy.Dempsey@sydney.edu.au	Senior Research Fellow, Cancer Health Policy. NHMRC Clinical Trials Centre, University of Sydney
Mr Craig Lawn*	Consumer Representative
Dr Thomas Pennington Thomas.Pennington@melanoma.org.au	Melanoma Surgeon, MIA
Professor Andrew Spillane Andrew.Spillane@sydney.edu.au	Melanoma and Breast Cancer Surgeon, MIA; Professor of Surgical Oncology, Royal North Shore Hospital & University of Sydney.
Professor Georgina Long Georgina.Long@melanoma.org.au	Medical Oncologist, MIA; Co-Director, MIA
Assoc Professor Alex Menzies Alex.Menzies@melanoma.org.au	Medical Oncologist, MIA
Professor Frances Boyle Frances.Boyle@sydney.edu.au	Medical Oncologist, MIA; Director, Patricia Ritchie Centre for Cancer Care and Research, Mater Hospital, North Sydney; Professor of Medical Oncology, University of Sydney
Dr Mbathio Dieng Mbathio.Dieng@sydney.edu.au	Postdoctoral Fellow, NHMRC Clinical Trials Centre, University of Sydney
Dr Skye Dong Skye.Dong@melanoma.org.au	Clinical Psychologist, MIA
Mr Samuel Herzog Samuel.Herzog@melanoma.org.au	Project Officer, ePROMs-MEL, MIA
Ms Shahn Coburn Shahn.Coburn@melanoma.org.au	Clinical Nurse Consultant, MIA
Ms Kate Willis Kate.Willis@melanoma.org.au	Clinical Nurse Consultant, MIA
Ms Rebecca Biviano Rebecca.Biviano@melanoma.org.au	Clinical Nurse Consultant, MIA
Ms Natalie Osborne Natalie.Osborne@melanoma.org.au	Clinical Nurse Consultant, RPAH
Ms Emel Fikri Emel.Fikri@health.nsw.gov.au	Melanoma Nurse, RPAH
Dr Donna Milne Donna.Milne@petermac.org	Melanoma Nurse, Peter MacCallum Cancer Centre
Mr Jake Thompson Jake.Thompson@melanoma.org.au	Research Officer ePROMs-MEL, MIA

For peer review only

BMJ Open

Embedding electronic Patient Reported Outcome Measures into routine care for patients with Stage III MELanoma (ePROMs-MEL): protocol for a prospective, longitudinal, mixed-methods pilot study.

Journal:	*BMJ Open*
Manuscript ID	bmjopen-2022-066852.R1
Article Type:	Protocol
Date Submitted by the Author:	22-Sep-2022

Complete List of Authors:	Dempsey, Kathy; The University of Sydney; Melanoma Institute Australia,<br>Saw, Robyn; Melanoma Institute Australia; The University of Sydney<br>Bartula, Iris; Melanoma Institute Australia<br>Lo, Serigne; Melanoma Institute Australia<br>Lawn, Craig; Melanoma Institute Australia<br>Pennington, Thomas; Melanoma Institute Australia,<br>Spillane, Andrew; Melanoma Institute Australia; The University of Sydney<br>Boyle, Frances ; Melanoma Institute Australia; The University of Sydney<br>Dong, Skye; Melanoma Institute Australia,<br>Dieng, Mbathio; The University of Sydney<br>Milne, Donna; Peter MacCallum Cancer Centre<br>Seaman, Linda; Melanoma Institute Australia<br>Saks, Dina; Melanoma Institute Australia,<br>Lai-Kwon, Julia; Melanoma Institute Australia,<br>Thompson, Jake; Melanoma Institute Australia<br>Morton, Rachael; The University of Sydney; Melanoma Institute Australia

**Primary Subject Heading**:	Patient-centred medicine
Secondary Subject Heading:	Patient-centred medicine, Oncology, Health services research, Qualitative research
Keywords:	Quality in health care < HEALTH SERVICES ADMINISTRATION & MANAGEMENT, MENTAL HEALTH, Dermatological tumours < ONCOLOGY, QUALITATIVE RESEARCH, Organisation of health services < HEALTH SERVICES ADMINISTRATION & MANAGEMENT

SCHOLARONE™
Manuscripts

Title

Embedding electronic Patient Reported Outcome Measures into routine care for patients with Stage III MELanoma (ePROMs-MEL): protocol for a prospective, longitudinal, mixed-methods pilot study.

Authors:

Dr Kathy Dempsey, BBSc, MA (Govt), PhD (Corresponding author)
Senior Research Fellow
National Health & Medical Research Council (NHMRC) Clinical Trials Centre,
92-94 Parramatta Rd,
Camperdown NSW 2050.
The University of Sydney, NSW, Australia.
Phone: +61 2 9562 5014
Fax: +61 2 9565 1863
Email: kathy.dempsey@sydney.edu.au
ORCID ID: 0000-0003-4945-0857

Associate Professor Robyn PM Saw
Email: robyn.saw@melanoma.org.au
Melanoma Institute Australia
Wollstonecraft NSW 2065
Australia
ORCID ID: 0000-0002-3354-806X

Dr Iris Bartula
Email: iris.bartula@melanoma.org.au
Melanoma Institute Australia
Wollstonecraft NSW 2065
Australia
ORCID ID: 0000-0002-4084-4115

Associate Professor Serigne Lo
Email: serigne.lo@melanoma.org.au
Melanoma Institute Australia
Wollstonecraft NSW 2065
Australia
ORCID ID: 0000-0001-5092-5544

Mr Craig Lawn
Email: lawncorporate@bigpond.com
Melanoma Institute Australia
Wollstonecraft NSW 2065
Australia

Dr Thomas Pennington
Email: thomas.pennington@melanoma.org.au
Melanoma Institute Australia
Wollstonecraft NSW 2065
Australia

Professor Andrew Spillane

For peer review only

For peer review only - http://bmjopen.bmj.com/site/about/guidelines.xhtml

Email: andrew.spillane@sydney.edu.au
Melanoma Institute Australia
Wollstonecraft NSW 2065
Australia
ORCID ID: 0000-0002-8354-434X

Professor Frances Boyle
Email: frances.boyle@sydney.edu.au
Melanoma Institute Australia
Wollstonecraft NSW 2065
Australia
ORCID ID: 0000-0003-3798-1570

Dr Skye Dong
Email: skye.dong@melanoma.org.au
Melanoma Institute Australia
Wollstonecraft NSW 2065
Australia

Dr Mbathio Dieng
Email: Mbathio.dieng@sydney.edu.au
NHMRC Clinical Trials Centre,
The University of Sydney, NSW, Australia
ORCID ID: 0000-0001-6714-9633

Mrs Donna Milne
Email: donna.milne@petermac.org.au
Peter MacCallum Cancer Centre
305 Grattan Street
Melbourne VIC 3000
Australia
ORCID ID: 0000-0002-9874-2023

Mrs Linda Seaman
Email: seaman.linda@gmail.com
Melanoma Institute Australia
Wollstonecraft NSW 2065
Australia

Dr Dina Saks
Email: dinayaelsaks@gmail.com
Melanoma Institute Australia
Wollstonecraft NSW 2065
Australia

Dr Julia Lai-Kwon
Email: julia.lai-kwon@melanoma.org.au
Melanoma Institute Australia
Wollstonecraft NSW 2065

For peer review only

Australia
ORCID ID: 0000-0002-5388-1695

Mr Jake Thompson
Email: jake.thompson@melanoma.org.au
Melanoma Institute Australia
Wollstonecraft NSW 2065
Australia
ORCID ID: 0000-0002-0823-3218

Professor Rachael L. Morton
Email: Rachael.morton@sydney.edu.au
NHMRC Clinical Trials Centre,
The University of Sydney, NSW, Australia.
ORCID ID: 0000-0001-7834-0572

Key words

Quality in health care, mental health, dermatological tumours, qualitative health, organisation of health services

Word count

3,994 words

Abstract

Introduction. The benefits of patient-reported feedback, using questionnaires that allow patients to report how they feel and function without any interpretation from healthcare professionals, are well-established. However, Patient-Reported Outcomes Measures (PROMs) are not routinely collected in melanoma patients in Australia. The primary aim is to evaluate the feasibility and acceptability of implementing electronic PROMs (ePROMs) into routine care from the perspectives of Stage III melanoma patients and their treating clinical team.

Methods and analysis. A minimum of 50 patients and 5 clinicians will be recruited to this prospective, longitudinal pilot study using a mixed methods approach (quantitative PROMs questionnaires and end-of-study surveys with qualitative interviews), which commenced in May 2021 in surgical and medical melanoma clinics at two sites in metropolitan Sydney, Australia. Secondary aims include measurement of improvements in patients' emotional and physical health and well-being, and utility of real-time data capture and clinician feedback. All participants will

For peer review only - http://bmjopen.bmj.com/site/about/guidelines.xhtml

complete the Distress Thermometer and EQ-5D-5L questionnaires in the clinic using a tablet computer at baseline and two to three subsequent follow-up appointments. Participants who report a score of 4 or higher on the Distress Thermometer will be triaged to complete an additional three questionnaires: the QLQ-C30, DASS and MCQ-28.

Results will be generated in real-time; patients with psychosocial distress or poor quality of life will discuss possible referral to appropriate allied health services with their clinician. Data analysis includes descriptive demographic data, questionnaire domain scores at each time point, questionnaire completion, allied health referral and uptake rates. Thematic analysis of interviews will be conducted.

Ethics and dissemination. Ethics approval obtained from St Vincent's Hospital Human Research Ethics Committee on 19/09/2019 (2019/ETH10558) with amendments approved on 8/06/2022. Patient consent was obtained electronically prior to questionnaire commencement. Dissemination strategies will vary to target different audiences.

Registration. ANZ Clinical Trials Registry (anzctr.org.au/ACTRN12620001149954.aspx).

Strengths and limitations of this study

- This prospective, longitudinal cohort intervention pilot study, conducted at two sites, examines the perspectives of patients, clinicians and clinic staff.
- A stakeholder reference panel, with consumer representation, was formed in the planning stages of this study and was actively involved in study design.
- The study uses a triage system of distress screening to minimise survey burden for those who are not distressed and maximise information collection from those who are.
- Tablet computers (iPads) were chosen as the delivery platform because they are portable, have a user-friendly interface and provide a secure format for data management with their default encryption.
- Summary reports from the completed questionnaires, including the first use of the Melanoma Concerns Questionnaire-28 (MCQ-28), a bespoke melanoma PROMs questionnaire, are immediately forwarded to the treating clinician in real-time, so they can be discussed during the patient's consultation.

Introduction

For peer review only

For peer review only - http://bmjopen.bmj.com/site/about/guidelines.xhtml

Patient Reported Outcome Measures (PROMs) are questionnaires that allow patients to report how they feel and function without any interpretation from healthcare professionals.[1] PROMs can be used in the management of individual patients to screen for a particular condition (such as depression) and/or to monitor patient response to an appropriate intervention (such as psychological therapy).[1] Previous research has shown that the use of PROMs can improve clinician-patient communication. One study of women with gynaecological cancers found a PROMs questionnaire helped with discussion of symptoms not typically addressed for nearly all participants (95%, n = 40) and improved patient care for 97% (n = 41) of participants.[2] Other studies have found PROMs can facilitate clinician awareness and improve patient management,[3,4] contribute to prognostic information,[3] potentially prolong survival outcomes in patients with advanced cancer,[5] as well as allowing patients to play a greater role in the clinical decision making process.[1] Currently, PROMs are not routinely collected as part of melanoma treatment in Australia.

It has long been recognised that many cancer specialists are not good at identifying cues for depression and psychological distress.[6-7] Routine screening for distress is now recommended by the United States National Comprehensive Cancer Network[8] and the International Psycho-Oncology Society and 68 affiliated organisations have set a standard of care involving monitoring distress as the '6th vital sign'.[9] Australian guidelines support the use of electronic PROMs (ePROMs) for the timely detection of psychosocial distress, but the authors note that results of trials evaluating the efficacy of distress screening have been mixed.[9] Effective trials used strategies that minimised staff burden and ensured effective follow-up.[9]

Previous research has demonstrated increased anxiety, depression and lower quality of life in melanoma patients.[10-12] An Australian review of psychosocial support estimated 30% of all melanoma patients report levels of psychological distress indicative of the need for clinical intervention.[13] Stage III melanoma patients usually have more morbid surgery, including sentinel node biopsy or lymph node dissection, which impacts significantly on their quality of life. When patients with Stage III melanoma attend follow up appointments with their treating physician, the focus is primarily on detection and treatment of recurrence of disease. There is minimal focus on assessing the patients' quality of life or psychological health status, despite recommendations from The Cancer Institute NSW[14] and the Australian Commission on Safety and Quality in Health Care.[15] An audit of Australian cancer services reported that 'evidence from clinical settings suggests timely identification of distress is only effective in improving medical management and patient wellbeing when paired with structures supportive care referrals'.[16]

A 2022 report on melanoma in Australia[17] noted the strong evidence that:

- *Access to emotional support services improves the well-being of people with cancer*

For peer review only - http://bmjopen.bmj.com/site/about/guidelines.xhtml

- *The opportunity to discuss feelings with a member of the treatment team or counsellor decreases psychosocial distress*
- *Psycho-educational programs decrease anxiety and depression.*

This landmark report concluded that increased screening for supportive care services should be developed as a quality indicator of melanoma clinical care.[17]

While there has been extensive research on estimating the extent of psychosocial distress in a vast range of clinical settings, subsequent translation of that knowledge into programs and services currently lags behind gains in the medical treatment of advanced melanoma.[18] This gap between obtaining results from PROMs and using this information as the first step in a process to actually improve patient outcomes is an overdue and urgent area of research.

By including the collection and evaluation of ePROMs data into the routine care for patients with Stage III melanoma, the treating clinician will be able to identify patients who are experiencing deterioration in quality of life or psychological distress. Once identified, these patients may then be referred to the appropriate allied health professionals (e.g. psychologist, specialist nurse, physiotherapist, occupational therapist, social worker) for appropriate support.

The primary aim of this pilot study is to evaluate the feasibility and acceptability of implementing ePROMs into routine care from the perspectives of both Stage III melanoma patients and their treating clinical team (i.e. surgeons, dermatologists, medical oncologists, psychologists and melanoma nurses). If the pilot results demonstrate technical, administrative and logistical feasibility, as well as patient, clinician and financial acceptability, this pilot will be expanded to all melanoma clinics within Melanoma Institute of Australia (MIA). This expansion will provide an opportunity to collect and analyse a greater volume of data and to potentially serve as a model for wider roll-out of ePROMs across the public and private healthcare sectors in New South Wales (NSW) and other states and territories of Australia.

Methods and analysis

Patient and public involvement

A stakeholder reference panel was formed in the planning stages of this pilot study. Members of this panel included experts in the field of melanoma (various clinical and non-clinical specialties), experts in patient reported measures research, and a small group of representatives/advocates for patients

For peer review only - http://bmjopen.bmj.com/site/about/guidelines.xhtml

with current and previously treated melanoma. Consumer involvement was considered vital to ensure that the needs and preferences of patients were incorporated alongside the knowledge and background of treating clinicians and researchers.

This Stakeholder Representative Panel was responsible for drafting a detailed project proposal to be funded as part of the Australian National Health and Medical Research Council Centre for Research Excellence in Melanoma. Panel tasks included: selection of the most appropriate PROMs to be included in this study, with consideration of minimising patient burden while maximising data usefulness; the content of semi-structured qualitative interview schedules (for patients and clinicians); development of all patient information documents, including the Participant Information Statement and Consent Forms (PISCFs), an Expression of Interest (EoI) form and the “About you” demographic patient information collection form; and clinician education resources including the development of short videos about the project and advice on discussion structure, in both electronic and written formats.

In addition to the documents, the manner of recruitment was widely discussed at these meetings, as was the creation of a comprehensive REDCap database that would receive results directly from the i-Pad custom-designed software while ensuring patient confidentiality. Consumer views also strongly influenced the plan for dissemination of results to patients, clinicians and their general practitioners..

Study design and setting

Prospective, longitudinal intervention study incorporating mixed methods (quantitative surveys and qualitative interviews). Although there is no control group, the design incorporates before-and-after study design elements allowing for comparisons of psychosocial health and quality of life in individuals with Stage III melanoma before and after the introduction of ePROMs. This study will be conducted at surgical and medical melanoma clinics at MIA and at Royal Prince Alfred Hospital (RPAH), Sydney, NSW, Australia.

Eligibility criteria and sample size

Patients are eligible to participate if they are aged 18 or over, have received a diagnosis of Stage III melanoma at least one month earlier, are under the care of melanoma clinicians at MIA or RPAH, and have sufficient English and cognitive ability to comprehend study materials, provide informed consent and participate in the study. Clinicians, including surgical oncologists, medical oncologists,

For peer review only

dermatologists and melanoma general practitioners, are eligible to participate if they are currently treating and managing patients with stage III melanoma at MIA or RPAH.

This research project has experienced substantial delays to recruitment due to research staff being unable to enter clinic sites during COVID-19. In April 2022, the decision was made to reduce the initial recruitment target of 100 to 50, so that the trial may be completed before external funding expires. The target reduction is justified on the basis that this project is a non-randomised feasibility pilot study. Assuming 30% of Stage III melanoma patients have levels of distress that indicate the need for clinical intervention,[13] we would expect to identify approximately 17 patients within our sample who will be triaged for discussion of referrals to support services. This number is considered sufficient to inform a decision on whether or not to proceed to a larger-scale trial with additional sites.

Recruitment

Recruitment has been intermittent due to delays caused by COVID-19 (April-September 2020 and June 2021-March 2022). Recruitment commenced at MIA on 28 May 2021 and at RPAH on 5 July 2021. Recruitment was extended to medical oncology clinics at MIA in March 2022 to bolster recruitment numbers. Thirty-six patients and seven clinicians have been recruited since the study commenced in May 2021, with the study expected to be completed by June 2023.

Patient recruitment process

A study clinician will review a list of potentially eligible patients (with Stage III melanoma) obtained from the MIA Melanoma Research Database to check clinical eligibility (i.e. are still under the care of MIA clinicians at either site or have not progressed to Stage 4 melanoma). Updated lists will then be sent to each of the treating clinicians who will be able to remove patients they feel are not appropriate to invite to participate in the study for other reasons. The clinical nurse consultant or clinic secretary will then send the approved list of eligible patients to the project officer.

These patients will be initially contacted by the project officer prior to their scheduled clinical review appointments. The project officer will send them three documents along with a covering letter (either by email or post; posted documents will contain a stamped self-addressed envelope). The email/letter will briefly introduce the study, ask patients to read the invitation to participate and the Participant Information Statement (PIS), and to complete and return an EoI form, which provides patients with three options to select from: willing, potentially willing or not willing to participate. If

For peer review only - http://bmjopen.bmj.com/site/about/guidelines.xhtml

patients do not respond to the request to return the EoI, the project officer will telephone them once to check whether they have received it.

The project officer will follow up those patients who have indicated their willingness or potential willingness to participate, answer any questions and concerns they may have about the study and ask them to arrive 30 minutes earlier for their scheduled appointment to allow time to complete the questionnaires. The PIS contains contact numbers of the academic and clinical leads for this project so patients may contact them prior to making their decision to participate. When patients arrive at the clinic for a regular appointment, they will again be given the opportunity to discuss any questions they may have regarding their participation with the project officer who will greet them, obtain their electronic consent on the iPad and show them how to complete the questionnaires on the iPad.

Patients who were recruited with Stage III melanoma but progress to Stage IV disease during the study can choose whether to continue in the study or not. Any continuing Stage IV patients will be asked to complete an interview at the end of the study to provide an assessment of how helpful they found this project to be considering their changing treatment and needs.

Clinician recruitment process

Clinical staff will be informed about the project through education sessions at clinical and multi-disciplinary team (MDT) meetings and be given a written invitation to participate. Interested clinicians will be provided with an electronic PISCF to read. Either hard copies of signed consent or scanned electronic versions will be acceptable and collated by the project officer prior to the clinician seeing study patients. All clinicians who were approached to participate consented to the study and reviewed the study questionnaires with the patients.

Interventions

The study questionnaires will be repeated at subsequent regularly scheduled follow-up appointments with their clinicians, with a minimum of two months between patient completion of questionnaires. Questionnaires will be administered at baseline, with a minimum of two and a maximum of three, follow-up time points. iPads were chosen as the delivery platform because they are portable, have a user-friendly interface, are capable of hosting software that can automatically generate reports and associated documents and are a secure format for data management as they are encrypted by default. All questionnaires used in this study have been validated in previous

For peer review only - http://bmjopen.bmj.com/site/about/guidelines.xhtml
For peer review only

studies.

At each visit, all patients will be required to complete two short questionnaires that are used to assess their psychosocial distress and quality of life: 1. the National Comprehensive Cancer Network 's Distress Thermometer and Problem List for Patients;[19] and 2. EuroQoL's EQ-5D-5L and visual analogue scale.[20] Patients who score above the clinical cut off score on the Distress Thermometer (4 or above) will be triaged to complete an additional three questionnaires: the Melanoma Concerns Questionnaire (MCQ-28),[21] the Depression, Anxiety and Stress Scale (DASS-21)[22] and the European Organisation for Research and Treatment of Cancer (EORTC QLQ-C30).[23] These additional questionnaires will provide more detailed information about potential areas of support needs. Completion time for all five questionnaires is expected to be less than 30 minutes. All questionnaires are provided in Appendix 1.

Results from the completed questionnaires will be automatically generated, emailed to the clinic secretary and printed for the treating clinician in real-time, so the results can be discussed during their consultation, along with medical concerns. Patients who request a copy of their results (by selecting the appropriate box in the 'About You' survey) will receive a summary of their scores by email, prior to their imminent appointment. Participants who score 3 or above on any of the five domains covered in the EQ-5D-5L questionnaire[20] (mobility, self-care, usual activities, pain/discomfort and anxiety/depression) may require clinical intervention (3= moderate, 4= severe, 5 = extreme limitations). There are no melanoma-specific cut-off scores indicating the need for clinical intervention, for the QoL questionnaires MCQ-28[21] and EORTC QLQ-C30.[23] Changes in scores for these two questionnaires will be recorded at each time point, allowing for manual inspection of changes over time. A deterioration in EORTC QLQ-C30 scores of 10 points or more between visits is commonly regarded as clinically significant.[24,25] Recommendations and referrals for additional support services will be based on the questionnaire scores and clinical experience of the clinicians and be made in collaboration with the patient. Further information on reporting processes is available in Appendix 2.

After all rounds of patient questionnaires (baseline plus 2-3 follow-up rounds per patient) have been completed, all patients, clinicians and clinic staff involved in the project will be asked to complete an electronic survey providing quantitative and open-ended assessments of their experiences with the PROMs questionnaires. In addition, approximately 20% of patients (10 patients), all study clinicians and clinic staff with major involvement in the study will be invited by the research team to

For peer review only - http://bmjopen.bmj.com/site/about/guidelines.xhtml

participate in a short (10-minute) confidential interview to expand on their survey responses. Patients will be purposively sampled to maximise response variation, based on a range of individual views and experiences as identified from their survey results. Patients who complete an interview will be offered a $30 gift card to thank them for their time, while clinicians and clinic staff who participate in an interview will not be reimbursed. Figure 1 provides a study flow chart from the patient's perspective and Figure 2 provides the study overview from the clinician's perspective.

[FIGURE 1 HERE]

[FIGURE 2 HERE]

For peer review only

For peer review only - http://bmjopen.bmj.com/site/about/guidelines.xhtml

Outcomes

The primary intended outcome of this study is to determine whether completion of PROMs electronically are considered by both patients, clinicians and clinic staff as feasible and acceptable when incorporated into routine clinical care. Secondary outcomes include assessment of whether these measures help identify people who need and would benefit from further support/referral; the production of a framework for the data systems required to enable electronic data capture and feedback to clinicians, useful in many other applications and clinical specialties; and the utility of clinician training resources. The primary outcome has three components.

Patient perceptions

Patient ratings of feasibility and acceptability will be assessed in several ways. The first is by the proportion of eligible patients approached who completed baseline and at least one set of follow-up PROMs data. If 60% of patients meet this requirement, the study will be deemed feasible and acceptable overall.

Data on individual patient measures of feasibility and acceptability will also be collected. In the first set of two triage questionnaires, all questions are mandatory, but with the second set of three questionnaires, patients are not required to answer all questions. Metadata from the patients who are triaged to complete the second group of questionnaires will be collected to provide an estimate of the mean time taken to complete the PROMs questionnaires and the amount of missing data (providing a second measure of patient acceptability for those who completed all five questionnaires). If patients take more than 30 minutes to complete the five questionnaires, they will be considered as not having met this acceptability criteria. Additional measures will include changes in baseline and follow-up scores and the proportion of patients who: 'agree or strongly agree' that the PROMs were easy to complete and that the questions they completed measured things they considered important to their QoL. Semi-structured interviews with patients will also assess feasibility and acceptability through the following main question themes: enjoyment of completing the PROMs; changes in patient-clinician communication; changes in their management; and improvements or challenges they found in the processes.

Clinician perceptions

It is also important to assess the feasibility and acceptability of this project from the clinicians' perspective. Measures include whether they found the PROMs data provided to them to be useful, timely, easy to understand and appropriate for their patient population. Clinicians will be approached to expand on their survey responses in a semi-structured interview. The key themes to address are related specifically to the PROMs instruments and the information generated for the clinician; changes in patient management; improvements in patient-clinician communication; and any key challenges or improvements that they can identify regarding the processes.

For peer review only - http://bmjopen.bmj.com/site/about/guidelines.xhtml

Clinic staff perceptions

Clinic staff including clinical nurse consultants and medical secretaries will also be asked to assess the feasibility and acceptability of incorporating QoL questionnaires into their clinic's routine practice. If they consider the additional workload or clinic disruption to be unacceptable or burdensome, then this will have a major impact on the feasibility assessment of continuing this practice once the pilot study has been completed.

Secondary outcomes

Research and clinical psychologists at MIA will use identified unmet needs from this study to inform future research into the development of support programs at MIA. The extent to which the routine use of PROMs in clinical practice can improve patients' emotional and physical problems will be assessed through changes in referral rates, referral uptake rates and patients' QoL scores over time. Patient and clinician interviews will allow for more extensive evaluation of this aspect of the study.

The utility of this framework for the data systems required to enable electronic data capture and feedback to clinicians will be assessed in patient, clinician and clinic staff surveys and interviews as well as assessment by the project team. End of study surveys will highlight the perceived ease of use, usefulness, relevance and importance of electronic PROMs to each group (see Appendix 3). Data will also be collected on time taken to complete questionnaires and on the number of times the questionnaires were completed (out of a maximum of three to four time points). In addition, the number of questions answered in the second set of three questionnaires (all questions in the first set of two questionnaires are mandatory) will be recorded.

All participating clinicians are experienced in treating Stage III melanoma patients and will be asked to review resources prepared for this study. These include three short videos about PROMs, referral pathways and the project details, and support services referral information on the MIA intranet. The aim of these resources is to help clinicians familiarise themselves with the objectives of the study and the use of PROMs in an oncology setting. Table 1 provides a timeline of expected outcomes.

For peer review only

For peer review only - http://bmjopen.bmj.com/site/about/guidelines.xhtml

Table 1. Study Interventions and Assessments Timeline

	Baseline			End of study
**TIME POINTS**[1]	T0	T1	T2	T3
ENROLMENT				
Eligibility screen	X			
Informed consent	X			
Recruitment	X			
INTERVENTIONS				
Baseline patient questionnaires	X			
Baseline patient demographic info	X			
Follow-up patient questionnaires		X	X	X
Clinician completion surveys				X
Patient completion surveys				X
Clinician completion interviews				X
Patient completion interviews				X
Clinician referral data				X
Patient referral uptake data				X
Patient change over time (T0-T5)				X
ASSESSMENTS				
Clinician acceptance				X
Patient acceptance				X
Clinic staff acceptance				X
Referral rate				X
Referral uptake rate				X

Table 1 adapted from the template designed by the SPIRIT Group ©2013[26]
1. For each time point, a window of +/- 2 months is acceptable due to variation in routine clinical appointments.

The overall success of the project will be assessed using the Proctor et al. framework for implementation research[27] which considers not only the intervention outcomes (service and client) but also the implementation outcomes, as outlined in Fig. 3.

[FIGURE 3 HERE]

Data analysis / Statistical methods

Quantitative analysis

Participant demographics and characteristics will be summarised for all participants using means and standard deviations. In addition, the consent, participation, and completion rates will be analysed. Consent will be defined

For peer review only - http://bmjopen.bmj.com/site/about/guidelines.xhtml

as agreeing to participate in the study and returning a signed consent form; participation, as completing some of the components of the study; and completion, as completing all components of the study. Specifically, the proportion of eligible participants who completed the project tasks will be assessed to determine whether the inclusion of ePROMs are acceptable for patients undergoing follow-up treatment for stage III melanoma. Descriptive statistics will be reported for QoL and other questionnaire domain scores at baseline and follow-up time points. Referral and referral uptake rates will also be calculated. The small sample size of 50 for this pilot study precludes any sub-group analysis.

Qualitative analysis

Results from semi-structured patient interviews conducted at the conclusion of the project will be reported to assess patient views on the usability, benefits and limitations of the study questionnaires they completed, whether questionnaire results were discussed within the subsequent clinical consultation, and if so whether this led to any change in management. Similarly, results from end of study clinician interviews will report their views on the feasibility, acceptability, benefits, limitations and usefulness of including ePROMs into the routine care for patients within their practice. Electronic qualitative analysis software (NVivo) will be used to assist in the development of a framework of key themes identified from the interview data that will then form the basis of a thematic analysis.[28]

Ethics and dissemination

Original ethics approval was obtained from St Vincent's Hospital Human Research Ethics Committe(HREC) on 19/09/2019 (2019/ETH10558). This approval included site authorisation for the following two project sites:

- Sydney Melanoma & Surgical Oncology, MIA, Poche Centre
- Sydney Melanoma & Surgical Oncology, RPAH Camperdown

Amendments to the original protocol were accepted by St Vincent's HREC on 8 June 2022.

Consent was obtained electronically on the iPad from all patients prior to commencing the questionnaires, while clinical staff signed written consent forms. All participants who complete end-of-study surveys or interviews will sign additional written consent forms. There are no foreseeable harms, risks or costs associated with taking part in this study. It is possible some patients may experience short-term distress while answering the questionnaires. If a patient does not wish to answer a question, they may skip it and go to the next question, or immediately stop completing the questionnaires. A melanoma clinical nurse consultant and/or treating clinician will be available to talk with a distressed patient if their distress continues once they have stopped completing the questionnaire. Patients are free to withdraw from the study at any time. Clinicians may experience frustration with having to review patient summary scores from the questionnaires if they are particularly busy.

For peer review only

For peer review only - http://bmjopen.bmj.com/site/about/guidelines.xhtml

Communication of trial results

Study results will be disseminated to participants via a short, lay summary of overall study findings at the end of the study. Patients can also opt in to receive their personal questionnaire results at each scheduled visit over the study time period. Overall study results will be communicated to participating clinicians and local healthcare professionals through multidisciplinary team meetings and to wider clinician audiences through presentations to the national surgical oncology, medical oncology and nursing representative societies. In addition to the standard fora of publication in peer-reviewed journals, seminar/webinar presentations and presentations at scientific conferences, presentations and summary materials targeting consumers and their families will be developed. Such informal tailored presentations will involve our patients and their families in discussion of the study findings and implications of ongoing PROMs research for clinical practice.

Public access to data

De-identified data only will be made available through a nominated data repository on request. Public access to participant-level data will not be available due to patient privacy concerns.

Publications policy

A publications policy has been created for this study. It outlines processes for authorship and will guide the appropriate recognition of all contributors to the intellectual property developed within the study. It applies to all publications that present research arising from and/or receiving funding from the pilot study as well as any future expanded studies based on the pilot findings.

Authors' contributions to the protocol

All authors participated in the drafting of this manuscript, undertook critical review of the intellectual content and approved the final version to be published. In addition, each of the authors made specific contributions as outlined below.

Kathy Dempsey was responsible for updating the original version of the protocol and drafting the first version of the manuscript, ensuring changes to the protocol were approved by the University of Sydney Human Research Ethics Committee and for coordinating co-author responses. KD is also the corresponding author and guarantor of this manuscript and a member of the ePROMs-MEL Steering Committee.

Rachael L Morton, Robyn PM Saw, Iris Bartula, Serigne Lo and Craig Lawn are Steering Committee members who were responsible for the conception and design of this protocol and study. RLM is an experienced health services researcher and health economist, IB a clinical and research psychologist, SL an accomplished biostatistician and

For peer review only - http://bmjopen.bmj.com/site/about/guidelines.xhtml

CL has extensive consumer representative experience in melanoma. All made substantial contributions to the conception and design of this research. Frances Boyle contributed her expertise in the use of the psychometric measures, including the melanoma-specific Melanoma Concerns Questionnaire, and clinician-patient communication approaches for collected data. Skye Dong is an experienced clinical psychologist who advised on the use of PROMs in the clinical setting and played a major role in the selection of appropriate PROMs. She is also the study psychologist who clinicians refer distressed study participants to for counselling. Mbathio Dieng, an early career researcher and Donna Milne, a clinical nurse consultant, worked with the Steering Committee and second consumer representative Linda Seaman, to review the PROMs and contributed to discussions on their appropriateness, particularly in terms of optimising research yield while minimising patient and clinic burden. Jake Thompson contributed his project officer skills and experience in psychosocial research and provided assistance to patients for data collection in the clinics when required. Melanoma clinicians Thomas Pennington, Robyn Saw, Andrew Spillane, Dina Saks and Julia Lai-Kwon contributed to patient recruitment and, along with France Boyle, acted as clinical project champions for the delivery of psychological support services within these clinics. A full list of Investigators, including Steering Committee members, is available in Appendix 4.

Competing interest statements

RPMS has received honoraria for advisory board participation from Merck Sharp & Dohme (MSD), Novartis and Qbiotics and speaking honoraria from Bristol Myers Squibb (BMS).

Funding statement

KD’s salary is supported through the Australian National Health and Medical Research Council Centre for Research Excellence in Melanoma grant (Grant No.1135285). RPMS is supported by MIA. The project officer’s salary is supported by a grant from The University of Sydney’s DVCR Support Fund for COVID-19 impacted research. Support is also provided by Melanoma Institute Australia for IT programming and educational design, as well as access to the Melanoma Research Database to aid in the selection of eligible patients. The study funder has no role in, or influence on, the study design; collection, management, analysis, and interpretation of data; writing of the report; and the decision to submit the report for publication.

Acknowledgements

We would like to acknowledge the significant contribution of other colleagues to the development of this protocol:

Information technology staff: Ivy Tan and Ernie Wise

Former project officers: Sam Robinson and Emma Zhang

For peer review only - http://bmjopen.bmj.com/site/about/guidelines.xhtml

References

1. Bantug ET, Coles T, Smith KC, Snyder CF, Rouette J, Brundage MD. Graphical displays of patient-reported outcomes (PRO) for use in clinical practice: What makes a pro picture worth a thousand words? Patient Educ Couns. 2016;99: 483-490.

2. Webster EM, Burke WM, Ware HM, Jones BA, Vattakalam R, Tergas AI, Wright JD, Hou JY. Patient reported outcomes in evaluation of chemotherapy toxicity in women with gynecologic malignancies: A pilot study. Gynecol Oncol 2018;150:487-493.

3. Bouazza YB, Chiairi I, El Kharbouchi O, De Backer L, Vanhoutte G, Janssens A et al. Patient-reported outcome measures (PROMs) in the management of lung cancer: A systematic review. Lung Cancer 2017;113:140-151.

4. Kargo AS, Coulter A, Jensen PT, Steffensen KD. Proactive use of PROMs in ovarian cancer survivors: a systematic review. J Ovarian Res 2019;12:63.

5. Basch E, Deal AM, Dueck AC, Scher HI, Kris MG, Hudis C, Schrag D. Overall survival results of a trial assessing patient-reported outcomes for symptom monitoring during routine Cancer treatment. JAMA. 2017;318(2):197–198.

6. Passik SD, Dugan W, McDonald MV, Rosenfeld B, Theobald DE, Edgerton S. Oncologists' recognition of depression in their patients with cancer. J Clin Oncol 1998; 16:1594-1600.

7. Fallowfield L, Ratcliffe D, Jenkins V, Saul J. Psychiatric morbidity and its recognition by doctors in patients with cancer. Br J Cancer 2001; 84(8): 1011–1015.

8. National Comprehensive Cancer Network (NCCN). NCCN Clinical Practice Guidelines in Oncology. Distress Management. V2.2020. March 11 2020. NCCN.org [restricted access]

9. Butow PN, Price MA, Shaw JM, Turner J, Clayton JM, Grimison P, Rankin N. [on behalf of the Psycho-Oncology Co-operative Research Group (PoCoG)]. Clinical pathway for the screening, assessment and management of anxiety and depression in adult cancer patients: Australian guidelines. Psycho-oncology 2015;24(9):987-1001.

10. Dieng M, Butow P, Costa D, Morton R, Menzies S, Mireskandari S et al. Psychoeducational intervention to reduce fear of cancer recurrence in people at high risk of developing another primary melanoma: Results of a randomized controlled trial. J Clin Oncol 2016;34(36), 4405-4414.

For peer review only

For peer review only - http://bmjopen.bmj.com/site/about/guidelines.xhtml

11. Stamataki Z, Brunton L, Lorigan P, Green AC, Newton-Bishop J, Molassiotis A. Assessing the impact of diagnosis and the related supportive care needs in patients with cutaneous melanoma. Support Care Cancer 2015;23:779–789.

12. Fu H, Teleni L, Crichton M, Chan RJ. Supportive care and unmet needs in patients with melanoma. A mixed-methods systematic review. Support Care Cancer 2020. https://doi.org/10.1007/s00520-020-05464-3

13. Kasparian NA. Psychological stress and melanoma: Are we meeting our patients' psychological needs? Clin Dermatol 2013;31:41-46

14. Cancer Institute NSW. Patient-reported measures (PRMs). https://www.cancer.nsw.gov.au/patient-reported-measures [accessed 12/5/2020]

15. Australian Commission on Safety and Quality in Health Care. Evidence on the use of PROMs in clinical practice, clinical research, quality improvement and policy development.

16. Fradgley EA, Byrnes E, McCarter K, Rankin N, Britton B, Clover K, Carter G, Bellamy D, Paul CL. A cross-sectional audit of current practices and areas for improvement of distress screening and management in Australian cancer services: is there a will and a way to improve? Support Care Cancer. 2020;28: 249-259.

17. Insight Economics, Melanoma Institute Australia and Melanoma Patients Australia. State of the Nation. A Report into Melanoma – A National Health Priority. Final Report; Sydney: February 2022.

18. Dunn J, Watson M, Aitken JF, Hyde MK. Systematic review of psychosocial outcomes for patients with advanced melanoma. Psychooncology. 2017;26: 1722-1731.

19. The National Comprehensive Cancer Network (NCCN®) NCCN Distress Thermometer and Problem List for Patients. Version 2.2018, 02/23/18.

20. Herdman M, Gudex C, Lloyd A et al. Development and preliminary testing of the new five-level version of EQ-5D (EQ-5D-5L). Quality of Life Research 2011;20(10):1727-1736.

21. Winstanley J, Boyle F, Young T. Melanoma Concerns Questionnaire© [MCQ-28] Version 1. 31 Jan 2020.

22. Lovibond SH and Lovibond PF. (1995). Manual for the Depression Anxiety Stress Scales. (2nd. Ed.) Sydney: Psychology Foundation.

For peer review only

For peer review only - http://bmjopen.bmj.com/site/about/guidelines.xhtml

23. European Organisation for Research and Treatment of Cancer (EORTC) Quality of Life Group. Quality of Life Questionnaire-Cancer 30 (QLQ-C30). Version 3.0

24. Cocks K, King MT, Velikova G, Fayers PM, Brown JM. Quality, interpretation and presentation of European Organisation for Research and Treatment of Cancer quality of life questionnaire core 30 data in randomised controlled trials. Eur J Cancer. 2008;44: 1793-1798.

25. Musoro JM, Bottomley A, Coens C, Eggermont AM, King MT, Cocks, K et al. EORTC Melanoma Group and EORTC Quality of Life Group. Interpreting European Organisation for Research and Treatment for Cancer Quality of life Questionnaire core 30 scores as minimally importantly different for patients with malignant melanoma. Eur J Cancer 2018;104:169-181. doi: 10.1016/j.ejca.2018.09.005

26. Calvert M, Kyte D, Mercieca-Bebber R, Slade A, Chan A-W, King MT; and the SPIRIT-PRO Group. Guidelines for Inclusion of Patient-Reported Outcomes in Clinical Trial Protocols: The SPIRIT-PRO Extension. JAMA. 2018;319(5):483-494.

27. Proctor E, Silmere H, Raghavan R, et al. Outcomes for implementation research: conceptual distinctions, measurement challenges, and research agenda. Administration and Policy in Mental Health and Mental Health Services Research. 2011;38(2):65-76.

28. Gale NK, Heath G, Cameron E, Rashid S, Redwood S. Using the framework method for the analysis of qualitative data in multi-disciplinary health research. BMC Medical Research Methodology 2013;13:117. doi:10.1186/1471-2288-13-117.

Figure Legend

Figure 1. Patient's perspective

Figure 2. Clinician's perspective

Figure 3: Implementation Research Outcomes [ref 27]

For peer review only

For peer review only - http://bmjopen.bmj.com/site/about/guidelines.xhtml

ePROMS-MEL FLOW CHART

PATIENTS

1. Recruitment

Potentially eligible patients identified by clinic staff. Suitability for recruitment checked with each patient's clinician. Project Officer (PO) sends Invitation to Participate, Participant Information Statement (PIS) and Expression of Interest form to eligible patients.

2. Consent

PO greets patient, answers any study-related questions and shows them how to use the iPad. Consent obtained electronically prior to commencing questionnaires.

3. Questionnaire data

Patients complete questionnaires on iPad while waiting for routine appointment. Data is wiped from iPad once submitted and is regularly uploaded into REDCap database.

4. Demographic data

All patients complete an "About you" questionnaire at Baseline, including age, socioeconomic indicators, living situation, income and education.

5. Triage questionnaire

NCCN Distress Thermometer (DT) with Problem List and EQ-5D-5L questionnaires completed by all patients at Baseline and regular follow-up appointments over 12-18 months.

6. Other questionnaires

Patients with scores of ≥ 4 on the DT are automatically presented with a further three QoL questionnaires (MCQ-28, DASS 21, QLQ C30).

7. Real-time reporting

Electronic report of scores on each domain generated for clinicians (and patients who requested it) in real time. Scores trigger referral as necessary (see clinician flow chart).

8. Completion surveys

All patients complete a brief survey on whether the questionnaires were easy to use, relevant and beneficial to their treatment.

9. Completion interviews

A 20% sample of patients asked to participate in interviews to expand on their survey responses.

10. Analysis

Process information collected. Quantitative (post-study surveys) and qualitative (post-study interviews) used to analyse acceptability of using PROMs in routine practice from patients' viewpoint.

ePROMS-MEL FLOW CHART

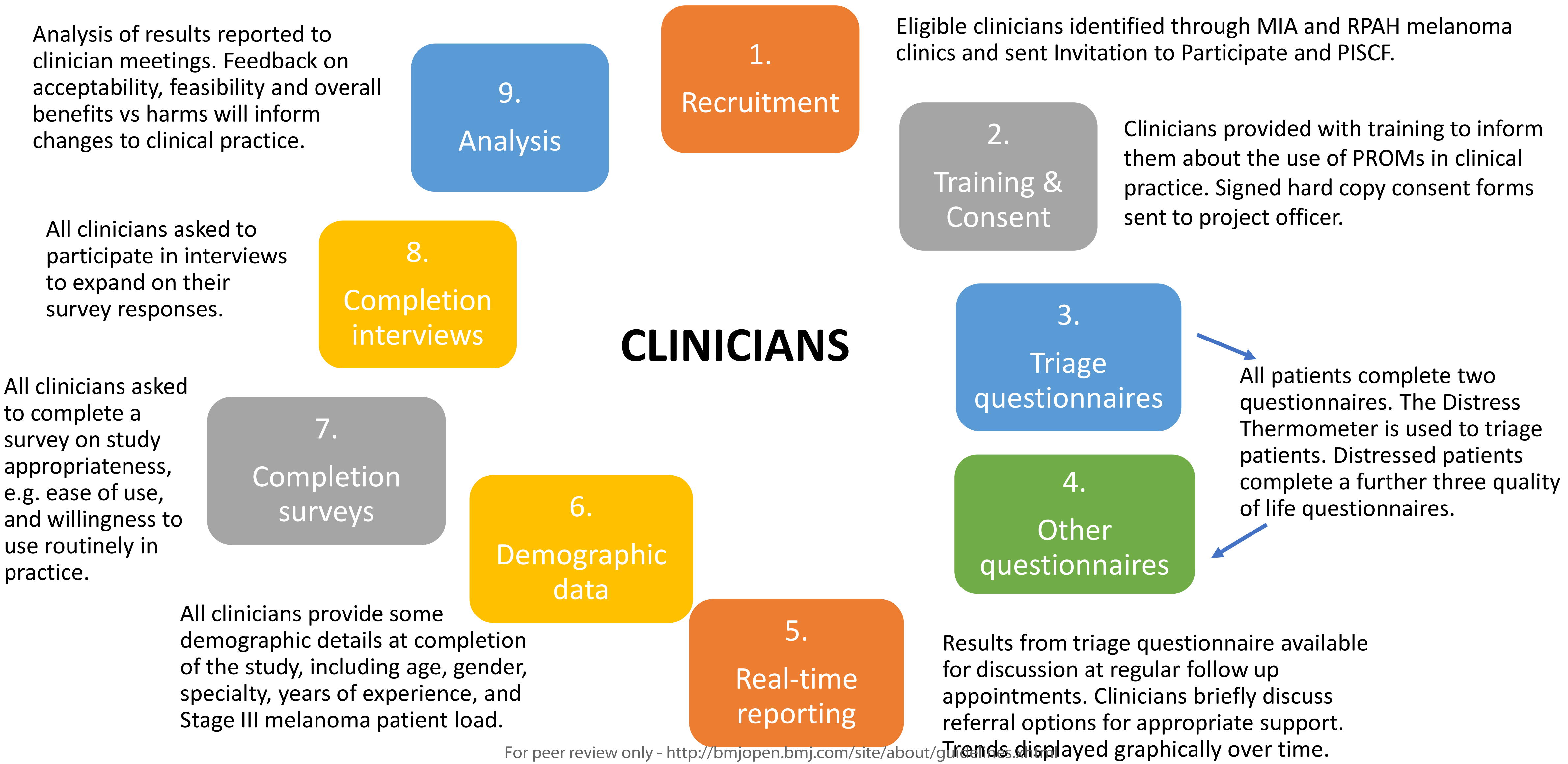

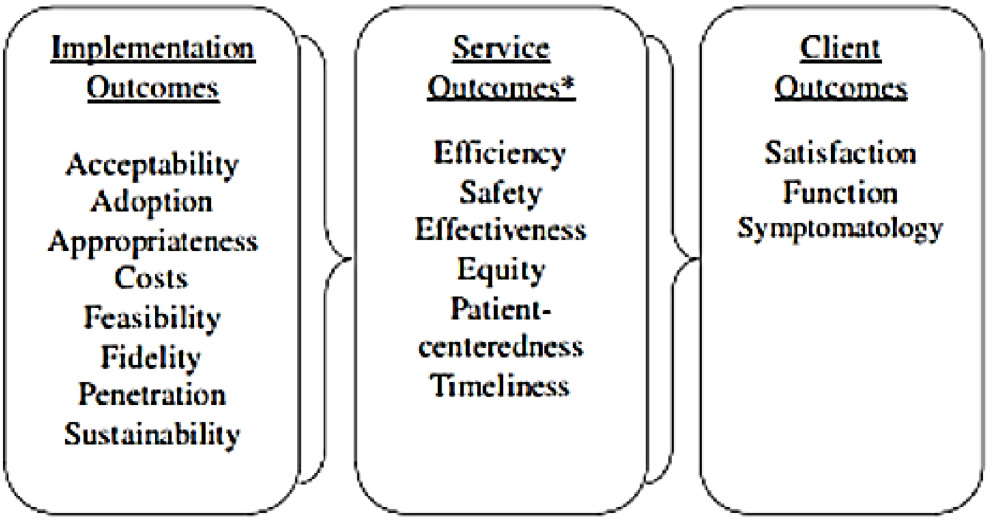

*IOM Standards of Care

Procter et al. See Reference 27

eer review only

NCCN National Comprehensive Cancer Network®

APPENDIX 1

NCCN Distress Thermometer and Problem List for Patients

NCCN DISTRESS THERMOMETER

Instructions: Please circle the number (0–10) that best describes how much distress you have been experiencing in the past week including today.

Extreme distress

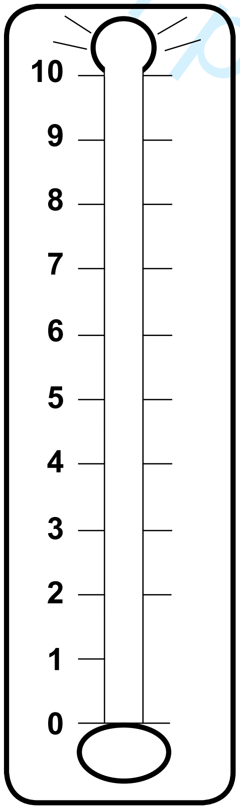

No distress

PROBLEM LIST

Please indicate if any of the following has been a problem for you in the past week including today.

Be sure to check YES or NO for each.

YES	NO	**Practical Problems**
❑	❑	Child care
❑	❑	Housing
❑	❑	Insurance/financial
❑	❑	Transportation
❑	❑	Work/school
❑	❑	Treatment decisions

YES	NO	**Family Problems**
❑	❑	Dealing with children
❑	❑	Dealing with partner
❑	❑	Ability to have children
❑	❑	Family health issues

YES	NO	**Emotional Problems**
❑	❑	Depression
❑	❑	Fears
❑	❑	Nervousness
❑	❑	Sadness
❑	❑	Worry
❑	❑	Loss of interest in usual activities

YES	NO	
❑	❑	**Spiritual/religious concerns**

YES	NO	**Physical Problems**
❑	❑	Appearance
❑	❑	Bathing/dressing
❑	❑	Breathing
❑	❑	Changes in urination
❑	❑	Constipation
❑	❑	Diarrhea
❑	❑	Eating
❑	❑	Fatigue
❑	❑	Feeling swollen
❑	❑	Fevers
❑	❑	Getting around
❑	❑	Indigestion
❑	❑	Memory/concentration
❑	❑	Mouth sores
❑	❑	Nausea
❑	❑	Nose dry/congested
❑	❑	Pain
❑	❑	Sexual
❑	❑	Skin dry/itchy
❑	❑	Sleep
❑	❑	Substance use
❑	❑	Tingling in hands/feet

Other Problems: ______________________________

____

Version 2.2018, 02/23/18. The NCCN Clinical Practice Guidelines (NCCN Guidelines®) are a statement of evidence and consensus of the authors regarding their views of currently accepted approaches to treatment. Any clinician seeking to apply or consult the NCCN Guidelines is expected to use independent medical judgment in the context of individual clinical circumstances to determine any patient's care or treatment. The National Comprehensive Cancer Network® (NCCN®) makes no representations or warranties of any kind regarding their content, use or application and disclaims any responsibility for their application or use in any way. The NCCN Guidelines are copyrighted by National Comprehensive Cancer Network®. All rights reserved. The NCCN Guidelines and the illustrations herein may not be reproduced in any form without the express written permission of NCCN. ©2018.

For peer review only
For peer review only - http://bmjopen.bmj.com/site/about/guidelines.xhtml

EQ-5D-5L (UK English sample version)

Under each heading, please tick the **ONE** box that best describes your health **TODAY**

MOBILITY

I have no problems in walking about

I have slight problems in walking about

I have moderate problems in walking about

I have severe problems in walking about

I am unable to walk about

SELF-CARE

I have no problems washing or dressing myself

I have slight problems washing or dressing myself

I have moderate problems washing or dressing myself

I have severe problems washing or dressing myself

I am unable to wash or dress myself

USUAL ACTIVITIES *(e.g. work, study, housework, family or leisure activities)*

I have no problems doing my usual activities

I have slight problems doing my usual activities

I have moderate problems doing my usual activities

I have severe problems doing my usual activities

I am unable to do my usual activities

PAIN / DISCOMFORT

I have no pain or discomfort

I have slight pain or discomfort

I have moderate pain or discomfort

I have severe pain or discomfort

I have extreme pain or discomfort

ANXIETY / DEPRESSION

I am not anxious or depressed

I am slightly anxious or depressed

I am moderately anxious or depressed

I am severely anxious or depressed

I am extremely anxious or depressed

SAMPLE

For peer review only

- We would like to know how good or bad your health is **TODAY**.
- This scale is numbered from **0** to **100**.
- **100** means the best health you can imagine.
  0 means the worst health you can imagine.
- Mark an **X** on the scale to indicate how your health is **TODAY**.
- Now, please write the number you marked on the scale in the box below.

YOUR HEALTH TODAY = [ ]

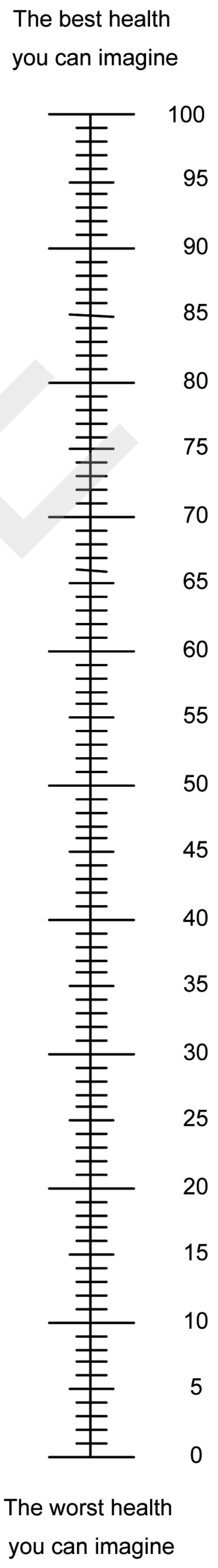

SAMPLE

For peer review only

MELANOMA CONCERNS QUESTIONNAIRE [MCQ-28] Patient Version

Patients sometimes report that they have the following concerns after their diagnosis and treatment for melanoma. Please indicate the extent to which you have experienced these symptoms or concerns.

Please answer by ticking the option that best applies to you.

	**Have you had surgery for your melanoma in the last 12 months?**<br>**Yes** ☐ **No** ☐<br>**If yes, during the past 4 weeks**	**Not at all**	**A little**	**Quite a bit**	**Very much**
**1**	Have you had swelling near your melanoma site?	○	○	○	○
**2**	Have you had numbness at the site of your melanoma?	○	○	○	○
**3**	Have you had problems with pain at or near your melanoma site?	○	○	○	○
**4**	How much have you worried about complications due to your melanoma surgery?	○	○	○	○
**5**	How much have you worried about the length of time needed for your melanoma surgery to heal?	○	○	○	○

	**Since the diagnosis and treatment of your melanoma**	**Not at all**	**A little**	**Quite a bit**	**Very much**
**6**	Have you worried about the increased risk of melanoma for other members of your family?	○	○	○	○
**7**	Have you had any regrets about your exposure to sunshine in the past?	○	○	○	○
**8**	Have you felt concerned about conducting outdoor activities (e.g. working, swimming, walking, sport) which may result in exposure to the sun?	○	○	○	○
**9**	Have you considered making any significant changes to how you will live your life in the future?	○	○	○	○
**10**	Have you worried whilst waiting for results of medical tests?	○	○	○	○
**11**	Have you felt confused by information about your diagnosis or treatment from different sources (e.g. internet)?	○	○	○	○

	Since the diagnosis and treatment of your melanoma	Not at all	A little	Quite a bit	Very much
12	Have you felt hopeful for the future?	○	○	○	○
13	Have you felt able to face the challenges ahead?	○	○	○	○
14	Have you felt able to cope with your diagnosis of melanoma?	○	○	○	○
15	Have you felt able to deal with the shock of being diagnosed with melanoma?	○	○	○	○
16	Have you felt able to carry on with things as normal?	○	○	○	○
17	Have you felt able to feel positive?	○	○	○	○

	During the past 4 weeks		Not at all	A little	Quite a bit	Very much
18	If you work, have you had support from your employer to continue working during your treatment (e.g. flexible/reduced hours)	N/A	○	○	○	○
19	How much has your melanoma doctor supported you?		○	○	○	○
20	How much have your other healthcare professionals (e.g. nurse) supported you?		○	○	○	○
21	How much have your family members supported you?		○	○	○	○
22	How much has your primary care doctor supported you?		○	○	○	○
23	Have you felt able to contact the melanoma clinical staff if you needed to?		○	○	○	○
24	Have you felt confident that a psychological support service would be available if you needed it?		○	○	○	○
25	Have you been given enough time to think about the treatment options available to you?		○	○	○	○
26	Have you felt comfortable being intimate with your partner?	N/A	○	○	○	○
27	How much have those important to you been included in discussions about your treatment options?		○	○	○	○
28	Have you felt confident that your healthcare team communicate with you in a professional manner?		○	○	○	○

Depression, Anxiety and Stress Scale (DASS21)

For each statement below, please circle the number in the column that best represents how you have been feeling in the last week.

Statement	Did not apply to me at all	Applied to me to some degree or some of the time	Applied to me a considerable degree or a good part of the time	Applied to me very much or most of the time
1. I found it hard to wind down	0	1	2	3
2. I was aware of dryness of my mouth	0	1	2	3
3. I couldn't seem to experience any positive feeling at all	0	1	2	3
4. I experienced breathing difficulty (eg, excessively rapid breathing, breathlessness in the absence of physical exertion)	0	1	2	3
5. I found it difficult to work up the initiative to do things	0	1	2	3
6. I tended to over-react to situations	0	1	2	3
7. I experienced trembling (eg, in the hands)	0	1	2	3
8. I felt that I was using a lot of nervous energy	0	1	2	3
9. I was worried about situations in which I might panic and make a fool of myself	0	1	2	3
10. I felt that I had nothing to look forward to	0	1	2	3
11. I found myself getting agitated	0	1	2	3
12. I found it difficult to relax	0	1	2	3
13. I felt down-hearted and blue	0	1	2	3
14. I was intolerant of anything that kept me from getting on with what I was doing	0	1	2	3
15. I felt I was close to panic	0	1	2	3
16. I was unable to become enthusiastic about anything.	0	1	2	3
17. I felt I wasn't worth much as a person	0	1	2	3
18. I felt that I was rather touchy	0	1	2	3
19. I was aware of the action of my heart in the absence of physical exertion (eg, sense of heart rate increase, heart missing a beat)	0	1	2	3
20. I felt scared without any good reason.	0	1	2	3
21. I felt that life was meaningless	0	1	2	3

Lovibond, S.H. & Lovibond, P.F. (1995). Manual for the Depression Anxiety Stress Scales. (2nd. Ed.) Sydney: Psychology Foundation

For peer review only

ENGLISH

EORTC QLQ-C30 (version 3)

We are interested in some things about you and your health. Please answer all of the questions yourself by circling the number that best applies to you. There are no "right" or "wrong" answers. The information that you provide will remain strictly confidential.

Please fill in your initials: |__|__|__|__|
Your birthdate (Day, Month, Year): |__|__|__|__|__|__|__|__|
Today's date (Day, Month, Year): 31 |__|__|__|__|__|__|__|__|

	Not at All	A Little	Quite a Bit	Very Much
1. Do you have any trouble doing strenuous activities, like carrying a heavy shopping bag or a suitcase?	1	2	3	4
2. Do you have any trouble taking a long walk?	1	2	3	4
3. Do you have any trouble taking a short walk outside of the house?	1	2	3	4
4. Do you need to stay in bed or a chair during the day?	1	2	3	4
5. Do you need help with eating, dressing, washing yourself or using the toilet?	1	2	3	4

During the past week:

	Not at All	A Little	Quite a Bit	Very Much
6. Were you limited in doing either your work or other daily activities?	1	2	3	4
7. Were you limited in pursuing your hobbies or other leisure time activities?	1	2	3	4
8. Were you short of breath?	1	2	3	4
9. Have you had pain?	1	2	3	4
10. Did you need to rest?	1	2	3	4
11. Have you had trouble sleeping?	1	2	3	4
12. Have you felt weak?	1	2	3	4
13. Have you lacked appetite?	1	2	3	4
14. Have you felt nauseated?	1	2	3	4
15. Have you vomited?	1	2	3	4
16. Have you been constipated?	1	2	3	4

Please go on to the next page

For peer review only

ENGLISH

During the past week:	Not at All	A Little	Quite a Bit	Very Much
17. Have you had diarrhea?	1	2	3	4
18. Were you tired?	1	2	3	4
19. Did pain interfere with your daily activities?	1	2	3	4
20. Have you had difficulty in concentrating on things, like reading a newspaper or watching television?	1	2	3	4
21. Did you feel tense?	1	2	3	4
22. Did you worry?	1	2	3	4
23. Did you feel irritable?	1	2	3	4
24. Did you feel depressed?	1	2	3	4
25. Have you had difficulty remembering things?	1	2	3	4
26. Has your physical condition or medical treatment interfered with your family life?	1	2	3	4
27. Has your physical condition or medical treatment interfered with your social activities?	1	2	3	4
28. Has your physical condition or medical treatment caused you financial difficulties?	1	2	3	4

For peer review only

For the following questions please circle the number between 1 and 7 that best applies to you

29. How would you rate your overall health during the past week?

1 2 3 4 5 6 7

Very poor Excellent

30. How would you rate your overall quality of life during the past week?

1 2 3 4 5 6 7

Very poor Excellent

© Copyright 1995 EORTC Quality of Life Group. All rights reserved. Version 3.0

APPENDIX 2.

Data from the questionnaires completed by the patients are uploaded into a custom-designed digital platform which allows instant access to the results presented in a user-friendly print-out.

This page shows how the results are displayed when a patient has identified anxiety:

Melanoma Institute Australia

Patient Reported Outcome Measures

Full Clinician Report- ePROMS-MEL Study

Patient Name: XXXXX
Date: 14-09-2022
Time Point: Baseline
Study ID: M130

Patient has reported moderate or high distress and/or poor quality of life.

Distress Thermometer score: (7/10)
(A value ≥ 4 is significant)

Patient has identified these problems from the Patient Problems List:
Work/school
Nervousness
Worry

EQ-5D-5L questionnaire results:

MOBILITY: 1 - I have no problems in walking about

SELF-CARE: 1 - I have no problems washing or dressing myself

USUAL ACTIVITIES: 1 - I have no problems doing my usual activities

PAIN / DISCOMFORT: 1 - I have no pain or discomfort

ANXIETY / DEPRESSION: 2 - I am slightly anxious or depressed

VISUAL ANALOGUE SCALE (VAS) score: 70/100
Global health rating Range = 0 - 100
0 = worst health you can imagine
100 = best health you can imagine

DASS21 questionnaire results:

DEPRESSSION: Normal

ANXIETY: Moderate

STRESS: Normal

This page shows the form for the clinician to complete.

Clinician Report – ePROMs-MEL study

Melanoma Institute Australia

Patient Name: Mrs Pilot Patient
Date: 23/02/2022
Time point: Follow-up 3
Study ID: ___________

Clinician name: ___________________________

Patient identified as distressed

Distress Thermometer Score: 7/10

(≥4/10 is clinically significant)

Patient identified these problems:

- Housing
- Treatment decisions
- Sadness

Clinician record of referrals (Y/N)

Services	Clinician wants to refer	Service is available	Patient would accept referral	Patient referred directly	Patient referred via GP
Psychology					
Social work					
Physio					
OT					
Other (please specify)					
1.					
2.					

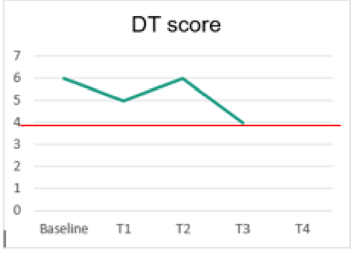

5

Full questionnaire data is uploaded from the iPad platform to a project REDCap database at the completion of recruitment and at the end of the study. This allows reports to be generated by the research team on aggregate data including trends over time, referrals and referral uptake. Reports on individual patients (e.g. to track changes in EORTC scores over time) can be generated at any time throughout the study.

view only

APPENDIX 3

Completion survey - patient

THE UNIVERSITY OF SYDNEY

NHMRC Clinical Trials Centre

Professor Rachael Morton
Director, Health Economics & Health Technology Assessment
Deputy Director, NHMRC Clinical Trials Centre

Camperdown, NSW 2050, Australia
Tel: 61-2-9562-5000
Fax: 61-2-9565-1863
Email: Rachael.morton@sydney.edu.au

Study ID number. ____________

Please circle/select the appropriate response.

1. Do you agree the questionnaires were easy to complete?

0	1	2	3	4
Strongly Disagree	Disagree	Neutral	Agree	Strongly Agree

Please provide reasons for your choice

____
____
____
____

2. Do you agree the questionnaires you completed measured the things that you consider important to your quality of life?

0	1	2	3	4
Strongly Disagree	Disagree	Neutral	Agree	Strongly Agree

Are there other questions you would have preferred to be asked in relation to your quality of life?

____
____
____
____

3. Do you agree your “whole of person” health and welfare has been improved because of the collection and consideration of the data on a real time basis with your clinician?

0	1	2	3	4
Strongly Disagree	Disagree	Neutral	Agree	Strongly Agree

For peer review only

Please provide reasons for your choice

____
____
____
____

4. Do you think your "whole of person" health and welfare has been improved because of the availability of support resources and groups? (0 = completely agree; 4 = completely disagree)

0	1	2	3	4
Strongly Disagree	Disagree	Neutral	Agree	Strongly Agree

Please provide reasons for your choice

____
____
____
____

5. Would you be willing to be contacted by the research team for a 10-20 minute confidential face to face or telephone interview to discuss your thoughts about the questions that you completed?

Yes No

If yes: Please provide your contact details below and a member of the research team will be in touch with you soon to discuss this further, and to provide you with a Participant Information Sheet and Consent Form for the interview.

Phone number: _________________________

Email address: ____

THANK YOU FOR COMPLETING THIS SURVEY.

Your time and cooperation is highly valued. We will keep you updated about the results.

The ePROMs-MEL team

.

For peer review only

Completion survey - **clinician**

THE UNIVERSITY OF SYDNEY

NHMRC Clinical Trials Centre

Professor Rachael Morton
Director, Health Economics & Health Technology Assessment
Deputy Director, NHMRC Clinical Trials Centre

Camperdown, NSW 2050, Australia
Tel: 61-2-9562-5000
Fax: 61-2-9565-1863
Email: Rachael.morton@sydney.edu.au

Name: ____

Please circle/select the appropriate response

1. Age group: <30 31-40 41-50 51-60 >60

2. Gender: Male Female Other

3. How many years' experience do you have managing patients with stage III melanoma?

   < 1 year 1-5 years 6-10 years 11-15 years 16-20 years >20 years

4. What is your medical specialty?
   Surgeon
   Oncologist
   Nurse
   Specialist GP
   Allied Health (please specify) ________________________________
   Other (please specify) ________________________________

5. Currently, how many patients with stage III melanoma do you manage per year?

   <20 21-50 51-100 >100

6. Do you agree the results of the PROMs were useful?

0	1	2	3	4
Strongly Disagree	Disagree	Neutral	Agree	Strongly Agree

Please provide reasons for your choice

____
____
____
____

For peer review only

7. Do you agree the format of information provided to you was easy to read and understand?

0	1	2	3	4
Strongly Disagree	Disagree	Neutral	Agree	Strongly Agree

If not – how could this be improved?

____
____
____

8. Do you agree that the questionnaires were appropriate for your patients' Stage of disease?

0	1	2	3	4
Strongly Disagree	Disagree	Neutral	Agree	Strongly Agree

If not appropriate – why not?

____
____
____

9. Do you agree the questionnaires were adequately detailed to capture your patients' concerns related to quality of life or current health status?

0	1	2	3	4
Strongly Disagree	Disagree	Neutral	Agree	Strongly Agree

If not – what additional questions or topics would you like to be included in future sets of ePROMs / ePREMs?

____
____
____
____

10. Would you consider implementing ePROMs / ePREMs for other patients that you manage?

Yes No

If yes – for what other patient populations would you consider implementing ePROMs/ ePREMs? *For example, stage II, stage IV etc.*

For peer review only

____
____
____

If no – why would you not implement ePROMs/ ePREMs?

____
____
____

11. Overall, do you agree that incorporating ePROMs / ePREMs in routine care for patients with stage III melanoma is feasible?

0	1	2	3	4
Strongly Disagree	Disagree	Neutral	Agree	Strongly Agree

If agree or strongly agree: What factors make incorporating ePROMs / ePREMs in routine care for patients with stage III melanoma feasible?

____
____
____

If disagree or strongly disagree: Why do you believe that incorporating ePROMs / ePREMs in routine care for patients with stage III melanoma is not feasible?

____
____
____

12. Would you be willing to be contacted by the research team for a 10-20 minute confidential interview to discuss the feasibility and practicality of incorporating ePROMs/ ePREMs into routine care for patients with stage III melanoma?

Yes No

If yes: Please provide your contact details below and a member of the research team will be in touch with you soon to discuss this further, and to provide you with a Participant Information Sheet and Consent Form for the interview.

Phone number:______________________

Email address:____

THANK YOU FOR PARTICIPATING IN THIS STUDY – we know how busy you are!
Your time and cooperation is highly valued. We will keep you updated about the results.

The ePROMs-MEL team.

For peer review only

Completion survey - clinic staff

THE UNIVERSITY OF SYDNEY

NHMRC Clinical Trials Centre

Professor Rachael Morton
Director, Health Economics & Health Technology Assessment
Deputy Director, NHMRC Clinical Trials Centre

Camperdown, NSW 2050, Australia
Tel: 61-2-9562-5000
Fax: 61-2-9565-1863
Email: Rachael.morton@sydney.edu.au

Please circle/select the appropriate response

1. Which clinic do you work in? (you may select more than one)

   SMSO Poche

   SMSO RPAH

2. Did you find the ePROMs project disruptive to clinic processes?

0	1	2	3	4
Strongly Disagree	Disagree	Neutral	Agree	Strongly Agree

If yes, in what ways?

____
____
____
____

3. Do you think the patients benefited from the project?

0	1	2	3	4
Strongly Disagree	Disagree	Neutral	Agree	Strongly Agree

If yes, in what ways?

____
____
____

For peer review only

4. Would you be pleased if ePROMs became part of routine care in your clinic?

0 Strongly Disagree	1 Disagree	2 Neutral	3 Agree	4 Strongly Agree

If no, why not?

____
____
____

5. Can you suggest changes that could be made to the project that would minimise clinic disruption?

Yes No

If yes, please share your suggestions below.

____
____
____
____

6. Would you consider implementing ePROMs for all patients in your clinic if additional resources were available?

Yes No

If yes, what additional resources would you want?

____
____
____

7. Would you be willing to be contacted by the research team for a 10-20 minute confidential interview to discuss the feasibility and practicality of incorporating ePROMs/ ePREMs into routine care for patients with stage III melanoma?

Yes No

If yes: Please provide your contact details below and a member of the research team will be in touch with you soon to discuss this further, and to provide you with a Participant Information Sheet and Consent Form for the interview.

Phone number:______________________

Email address:____

THANK YOU FOR PARTICIPATING IN THIS STUDY – we know how busy you are!
Your time and cooperation is highly valued. We will keep you updated about the results.
The ePROMs-MEL team.

For peer review only

APPENDIX 4. ePROMs-MEL Investigators

Professor Rachael Morton* (Chief Investigator) Rachael.Morton@ctc.usyd.edu.au	Director of Health Economics, NHMRC Clinical Trials Centre, The University of Sydney NSW Australia & Melanoma Institute Australia (MIA) Faculty
Associate Professor Robyn Saw* Robyn.Saw@melanoma.org.au	Melanoma Surgeon, MIA Faculty, Royal Prince Alfred Hospital (RPAH), and Mater Hospitals & University of Sydney
Associate Professor Sergine Lo* Serigne.Lo@melanoma.org.au	Senior Statistician, MIA Faculty and Senior Research Fellow in Biostatistics, The University of Sydney
Dr Iris Bartula* Iris.Bartula@melanoma.org.au	Research and Clinical Psychologist, MIA
Dr Kathy Dempsey* Kathy.Dempsey@sydney.edu.au	Senior Research Fellow, Cancer Health Policy. NHMRC Clinical Trials Centre, University of Sydney
Mr Craig Lawn*	Consumer Representative
Dr Thomas Pennington Thomas.Pennington@melanoma.org.au	Melanoma Surgeon, MIA
Professor Andrew Spillane Andrew.Spillane@sydney.edu.au	Melanoma and Breast Cancer Surgeon, MIA; Professor of Surgical Oncology, Royal North Shore Hospital & University of Sydney.
Professor Georgina Long Georgina.Long@melanoma.org.au	Medical Oncologist, MIA; Co-Director, MIA
Assoc Professor Alex Menzies Alex.Menzies@melanoma.org.au	Medical Oncologist, MIA
Professor Frances Boyle Frances.Boyle@sydney.edu.au	Medical Oncologist, MIA; Director, Patricia Ritchie Centre for Cancer Care and Research, Mater Hospital, North Sydney; Professor of Medical Oncology, University of Sydney
Dr Mbathio Dieng Mbathio.Dieng@sydney.edu.au	Postdoctoral Fellow, NHMRC Clinical Trials Centre, University of Sydney
Dr Skye Dong Skye.Dong@melanoma.org.au	Clinical Psychologist, MIA
Mr Samuel Herzog Samuel.Herzog@melanoma.org.au	Project Officer, ePROMs-MEL, MIA
Ms Shahn Coburn Shahn.Coburn@melanoma.org.au	Clinical Nurse Consultant, MIA
Ms Kate Willis Kate.Willis@melanoma.org.au	Clinical Nurse Consultant, MIA
Ms Rebecca Biviano Rebecca.Biviano@melanoma.org.au	Clinical Nurse Consultant, MIA
Ms Natalie Osborne Natalie.Osborne@melanoma.org.au	Clinical Nurse Consultant, RPAH
Ms Emel Fikri Emel.Fikri@health.nsw.gov.au	Melanoma Nurse, RPAH
Dr Donna Milne Donna.Milne@petermac.org	Melanoma Nurse, Peter MacCallum Cancer Centre
Mr Jake Thompson Jake.Thompson@melanoma.org.au	Research Officer ePROMs-MEL, MIA

For peer review only

BMJ Open

Embedding electronic Patient Reported Outcome Measures into routine care for patients with Stage III MELanoma (ePROMs-MEL): protocol for a prospective, longitudinal, mixed-methods pilot study

Journal:	*BMJ Open*
Manuscript ID	bmjopen-2022-066852.R2
Article Type:	Protocol
Date Submitted by the Author:	24-Nov-2022
Complete List of Authors:	Dempsey, Kathy; The University of Sydney; Melanoma Institute Australia,<br>Saw, Robyn; Melanoma Institute Australia; The University of Sydney<br>Bartula, Iris; Melanoma Institute Australia<br>Lo, Serigne; Melanoma Institute Australia<br>Lawn, Craig; Melanoma Institute Australia<br>Pennington, Thomas; Melanoma Institute Australia,<br>Spillane, Andrew; Melanoma Institute Australia; The University of Sydney<br>Boyle, Frances ; Melanoma Institute Australia; The University of Sydney<br>Dong, Skye; Melanoma Institute Australia,<br>Dieng, Mbathio; The University of Sydney<br>Milne, Donna; Peter MacCallum Cancer Centre<br>Seaman, Linda; Melanoma Institute Australia<br>Saks, Dina; Melanoma Institute Australia,<br>Lai-Kwon, Julia; Melanoma Institute Australia,<br>Thompson, Jake; Melanoma Institute Australia<br>Morton, Rachael; The University of Sydney,
**Primary Subject Heading**:	Patient-centred medicine
Secondary Subject Heading:	Patient-centred medicine, Oncology, Health services research, Qualitative research
Keywords:	Quality in health care < HEALTH SERVICES ADMINISTRATION & MANAGEMENT, MENTAL HEALTH, Dermatological tumours < ONCOLOGY, QUALITATIVE RESEARCH, Organisation of health services < HEALTH SERVICES ADMINISTRATION & MANAGEMENT

SCHOLARONE™
Manuscripts

Embedding electronic Patient Reported Outcome Measures into routine care for patients with Stage III MELanoma (ePROMs-MEL): protocol for a prospective, longitudinal, mixed-methods pilot study

Authors and affiliations:

Dr Kathy Dempsey, BBSc, MA (Govt), PhD
Email: kathy.dempsey@sydney.edu.au
Senior Research Fellow
National Health & Medical Research Council (NHMRC) Clinical Trials Centre,
92-94 Parramatta Rd,
Camperdown NSW 2050.
The University of Sydney, NSW, Australia.
ORCID ID: 0000-0003-4945-0857

Associate Professor Robyn PM Saw
Email: robyn.saw@melanoma.org.au
Melanoma Institute Australia
Wollstonecraft NSW 2065
Australia
ORCID ID: 0000-0002-3354-806X

Dr Iris Bartula
Email: iris.bartula@melanoma.org.au
Melanoma Institute Australia
Wollstonecraft NSW 2065
Australia
ORCID ID: 0000-0002-4084-4115

Associate Professor Serigne Lo
Email: serigne.lo@melanoma.org.au
Melanoma Institute Australia
Wollstonecraft NSW 2065
Australia
ORCID ID: 0000-0001-5092-5544

Mr Craig Lawn
Email: lawncorporate@bigpond.com
Melanoma Institute Australia
Wollstonecraft NSW 2065
Australia

Dr Thomas Pennington
Email: thomas.pennington@melanoma.org.au
Melanoma Institute Australia
Wollstonecraft NSW 2065
Australia

Professor Andrew Spillane
Email: andrew.spillane@sydney.edu.au
Melanoma Institute Australia
Wollstonecraft NSW 2065

For peer review only

For peer review only - http://bmjopen.bmj.com/site/about/guidelines.xhtml

Australia
ORCID ID: 0000-0002-8354-434X

Professor Frances Boyle
Email: frances.boyle@sydney.edu.au
Melanoma Institute Australia
Wollstonecraft NSW 2065
Australia
ORCID ID: 0000-0003-3798-1570

Dr Skye Dong
Email: skye.dong@melanoma.org.au
Melanoma Institute Australia
Wollstonecraft NSW 2065
Australia

Dr Mbathio Dieng
Email: Mbathio.dieng@sydney.edu.au
NHMRC Clinical Trials Centre,
The University of Sydney, NSW, Australia
ORCID ID: 0000-0001-6714-9633

Mrs Donna Milne
Email: donna.milne@petermac.org.au
Peter MacCallum Cancer Centre
305 Grattan Street
Melbourne VIC 3000
Australia
ORCID ID: 0000-0002-9874-2023

Mrs Linda Seaman
Email: seaman.linda@gmail.com
Melanoma Institute Australia
Wollstonecraft NSW 2065
Australia

Dr Dina Saks
Email: dinayaelsaks@gmail.com
Melanoma Institute Australia
Wollstonecraft NSW 2065
Australia

Dr Julia Lai-Kwon
Email: julia.lai-kwon@melanoma.org.au
Melanoma Institute Australia
Wollstonecraft NSW 2065
Australia
ORCID ID: 0000-0002-5388-1695

Mr Jake Thompson
Email: jake.thompson@melanoma.org.au
Melanoma Institute Australia

For peer review only

Wollstonecraft NSW 2065
Australia
ORCID ID: 0000-0002-0823-3218

Professor Rachael L Morton
Email: Rachael.morton@sydney.edu.au
NHMRC Clinical Trials Centre,
The University of Sydney, NSW, Australia.
ORCID ID: 0000-0001-7834-0572

Correspondence to:
Dr Kathy Dempsey, BBSc, MA (Govt), PhD
National Health & Medical Research Council (NHMRC) Clinical Trials Centre,
92-94 Parramatta Rd,
Camperdown NSW 2050,
The University of Sydney, NSW, Australia
Email: kathy.dempsey@sydney.edu.au

Keywords

Quality in health care, mental health, dermatological tumours, qualitative health, organisation of health services

Abstract

Introduction: The benefits of patient-reported feedback, using questionnaires that allow patients to report how they feel and function without any interpretation from healthcare professionals, are well established. However, Patient-Reported Outcomes Measures (PROMs) are not routinely collected in patients with melanoma in Australia. The aim of this study is to evaluate the feasibility and acceptability of implementing electronic PROMs (ePROMs) into routine care from the perspectives of patients with Stage III melanoma and their treating clinical team.

Methods and analysis: A minimum of 50 patients and five clinicians will be recruited to this prospective, longitudinal pilot study (ePROMs-MEL). The study uses a mixed methods approach (quantitative PROMs questionnaires and end-of-study surveys with qualitative interviews) and commenced in May 2021 in surgical and medical melanoma clinics at two sites in metropolitan Sydney, Australia. The primary outcomes are measures of feasibility and acceptability, comprising descriptive questionnaire completion statistics, and proportion of patients who reported that these PROMs were easy to complete and measured items they considered important. Clinician and clinic staff views will be canvassed on the appropriateness of these PROMs for their patients, change in referral practice and uptake and incorporation into routine practice. Secondary aims include measurement of improvements in patients' emotional and physical health and well-being, and utility of real-time data capture and clinician feedback. All participants will complete the Distress Thermometer and EQ-5D-5L questionnaires in the clinic using a tablet computer at baseline and two to three subsequent

For peer review only - http://bmjopen.bmj.com/site/about/guidelines.xhtml

follow-up appointments. Participants who report a score of 4 or higher on the Distress Thermometer will be triaged to complete an additional three questionnaires: the QLQ-C30, DASS and MCQ-28. Results will be generated in real-time; patients with psychosocial distress or poor quality of life will discuss possible referral to appropriate allied health services with their clinician. Thematic analysis of interviews will be conducted.

Ethics and dissemination: Ethics approval obtained from St Vincent's Hospital Human Research Ethics Committee on 19/09/2019 (2019/ETH10558), with amendments approved on 8/06/2022. Patient consent is obtained electronically prior to questionnaire commencement. Dissemination strategies will include publication in peer-reviewed journals and presentation at international conferences, tailored presentations for clinical societies and government bodies, organisational reporting through multi-disciplinary meetings and research symposia for local clinicians and clinic staff, and more informal, lay reports and presentations for consumer melanoma representative bodies and patient participants and their families.

Study registration number: Australian New Zealand Clinical Trials Registry, ACTRN12620001149954.

Strengths and limitations of this study

- This prospective, longitudinal cohort intervention pilot study, conducted at two sites, examines the perspectives of patients, clinicians and clinic staff.
- A stakeholder reference panel, with consumer representation, was formed in the planning stages of this study and is actively involved in oversight of the study.
- The study uses a triage system of distress screening to minimise survey burden for those who are not distressed and maximise information collection from those who are.
- Summary reports from the completed questionnaires, including the first use of the Melanoma Concerns Questionnaire-28 (MCQ-28), a bespoke melanoma PROMs questionnaire, are immediately forwarded to the treating clinician in real-time, so they can be discussed during the patient's consultation.
- Limitations include the absence of a control group and an unavoidable reduction in the participant enrolment target due to the impact of COVID-19 restrictions on recruitment.

Introduction

Patient Reported Outcome Measures (PROMs) are questionnaires that allow patients to report how they feel and function without any interpretation from healthcare professionals.[1] PROMs can be used in the management of individual patients to screen for a particular condition (such as depression) and/or to monitor patient response to an appropriate intervention (such as psychological therapy).[1] Previous research has shown that the

For peer review only

For peer review only - http://bmjopen.bmj.com/site/about/guidelines.xhtml

use of PROMs can improve clinician-patient communication. One study of women with gynaecological cancers found a PROMs questionnaire helped with discussion of symptoms not typically addressed for nearly all participants (95%, n = 40) and improved patient care for 97% (n = 41) of participants.[2] Other studies have found PROMs can facilitate clinician awareness and improve patient management,[3,4] contribute to prognostic information,[3] potentially prolong survival outcomes in patients with advanced cancer,[5] as well as allowing patients to play a greater role in the clinical decision making process.[1] Currently, PROMs are not routinely collected as part of melanoma treatment in Australia.

It has long been recognised that many cancer specialists are not good at identifying cues for depression and psychological distress.[6-7] Routine screening for distress is now recommended by the United States National Comprehensive Cancer Network[8] and the International Psycho-Oncology Society and 68 affiliated organisations have set a standard of care involving monitoring distress as the '6th vital sign'.[9] Australian guidelines support the use of electronic PROMs (ePROMs) for the timely detection of psychosocial distress, but the authors note that results of trials evaluating the efficacy of distress screening have been mixed.[9] Effective trials used strategies that minimised staff burden and ensured effective follow-up.[9]

Previous research has demonstrated increased anxiety, depression and lower quality of life in melanoma patients.[10-12] An Australian review of psychosocial support estimated 30% of all melanoma patients report levels of psychological distress indicative of the need for clinical intervention.[13] Stage III melanoma patients usually have more morbid surgery, including sentinel node biopsy or lymph node dissection, which impacts significantly on their quality of life. When patients with Stage III melanoma attend follow up appointments with their treating physician, the focus is primarily on detection and treatment of recurrence of disease. There is minimal focus on assessing the patients' quality of life or psychological health status, despite recommendations from The Cancer Institute NSW[14] and the Australian Commission on Safety and Quality in Health Care.[15] An audit of Australian cancer services reported that 'evidence from clinical settings suggests timely identification of distress is only effective in improving medical management and patient wellbeing when paired with structures supportive care referrals'.[16]

A 2022 report on melanoma in Australia[17] noted the strong evidence that:

- *Access to emotional support services improves the well-being of people with cancer*
- *The opportunity to discuss feelings with a member of the treatment team or counsellor decreases psychosocial distress*
- *Psycho-educational programs decrease anxiety and depression.*

This landmark report concluded that increased screening for supportive care services should be developed as a quality indicator of melanoma clinical care.[17]

For peer review only - http://bmjopen.bmj.com/site/about/guidelines.xhtml

While there has been extensive research on estimating the extent of psychosocial distress in a vast range of clinical settings, subsequent translation of that knowledge into programs and services currently lags behind gains in the medical treatment of advanced melanoma.[18] This gap between obtaining results from PROMs and using this information as the first step in a process to actually improve patient outcomes is an overdue and urgent area of research.

By including the collection and evaluation of ePROMs data into the routine care for patients with Stage III melanoma, the treating clinician will be able to identify patients who are experiencing deterioration in quality of life or psychological distress. Once identified, these patients may then be referred to the appropriate allied health professionals (e.g. psychologist, specialist nurse, physiotherapist, occupational therapist, social worker) for appropriate support.

The primary aim of this pilot study is to evaluate the feasibility and acceptability of implementing ePROMs into routine care from the perspectives of both Stage III melanoma patients and their treating clinical team (i.e. surgeons, dermatologists, medical oncologists, psychologists and melanoma nurses). If the pilot results demonstrate technical, administrative and logistical feasibility, as well as patient, clinician and financial acceptability, this pilot will be expanded to all melanoma clinics within Melanoma Institute Australia (MIA). This expansion will provide an opportunity to collect and analyse a greater volume of data and to potentially serve as a model for wider roll-out of ePROMs across the public and private healthcare sectors in New South Wales (NSW) and other states and territories of Australia.

Methods and analysis

Patient and public involvement

A stakeholder reference panel was formed in the planning stages of this pilot study. Members of this panel included experts in the field of melanoma (various clinical and non-clinical specialties), experts in patient reported measures research, and a small group of representatives/advocates for patients with current and previously treated melanoma. Consumer involvement is considered vital to ensure that the needs and preferences of patients were incorporated alongside the knowledge and background of treating clinicians and researchers.

This Stakeholder Representative Panel was responsible for drafting a detailed project proposal to be funded as part of the Australian National Health and Medical Research Council Centre for Research Excellence in Melanoma. Panel tasks included: selection of the most appropriate PROMs to be included in this study, with consideration of minimising patient burden while maximising data usefulness; the content of semi-structured qualitative interview schedules (for patients and clinicians); development of all patient information documents,

For peer review only - http://bmjopen.bmj.com/site/about/guidelines.xhtml
For peer review only

including the Participant Information Statement and Consent Forms (PISCFs), an Expression of Interest (EoI) form and the "About you" demographic patient information collection form; and clinician education resources including the development of short videos about the project and advice on discussion structure, in both electronic and written formats.

In addition to the documents, the manner of recruitment was widely discussed at these meetings, as was the creation of a comprehensive REDCap database that would receive results directly from the i-Pad custom-designed software while ensuring patient confidentiality. Consumer views also strongly influenced the plan for dissemination of results to patients, clinicians and their general practitioners.

Study design and setting

ePROMs-MEL is a prospective, longitudinal intervention study incorporating mixed methods (quantitative surveys and qualitative interviews). Although there is no control group, the design incorporates before-and-after study design elements allowing for comparisons of psychosocial health and quality of life in individuals with Stage III melanoma before and after the introduction of ePROMs. This study will be conducted at surgical and medical melanoma clinics at MIA and at Royal Prince Alfred Hospital (RPAH), Sydney, NSW, Australia.

Eligibility criteria and sample size

Patients are eligible to participate if they are aged 18 or over, have received a diagnosis of Stage III melanoma at least one month earlier, are under the care of melanoma clinicians at MIA or RPAH, and have sufficient English and cognitive ability to comprehend study materials, provide informed consent and participate in the study. Clinicians, including surgical oncologists, medical oncologists, dermatologists and melanoma general practitioners, are eligible to participate if they are currently treating and managing patients with stage III melanoma at MIA or RPAH.

This research project has experienced substantial delays to recruitment due to research staff being unable to enter clinic sites during COVID-19. In April 2022, the decision was made to reduce the initial recruitment target of 100 to 50 patients, so that the trial may be completed before external funding expires (the minimum number of clinician participants is five). The target reduction is justified on the basis that this project is a non-randomised feasibility pilot study. Assuming 30% of Stage III melanoma patients have levels of distress that indicate the need for clinical intervention,[13] we would expect to identify approximately 17 patients within our sample who will be triaged for discussion of referrals to support services. This number is considered sufficient to inform a decision on whether or not to proceed to a larger-scale trial with additional sites.

For peer review only

For peer review only - http://bmjopen.bmj.com/site/about/guidelines.xhtml

Recruitment

Recruitment has been intermittent due to delays caused by COVID-19 (April-September 2020 and June 2021-March 2022). Recruitment commenced at MIA on 28 May 2021 and at RPAH on 5 July 2021. Recruitment was extended to medical oncology clinics at MIA in March 2022 to bolster recruitment numbers. Thirty-six patients and seven clinicians have been recruited since the study commenced in May 2021, with the study expected to be completed by June 2023.

Patient recruitment process

A study clinician will review a list of potentially eligible patients (with Stage III melanoma) obtained from the MIA Melanoma Research Database to check clinical eligibility (i.e. are still under the care of MIA clinicians at either site or have not progressed to Stage 4 melanoma). Updated lists will then be sent to each of the treating clinicians who will be able to remove patients they feel are not appropriate to invite to participate in the study for other reasons. The clinical nurse consultant or clinic secretary will then send the approved list of eligible patients to the project officer.

These patients will be initially contacted by the project officer prior to their scheduled clinical review appointments. The project officer will send them three documents along with a covering letter (either by email or post; posted documents will contain a stamped self-addressed envelope). The email/letter will briefly introduce the study, ask patients to read the invitation to participate and the Participant Information Statement (PIS), and to complete and return an EoI form, which provides patients with three options to select from: willing, potentially willing or not willing to participate. If patients do not respond to the request to return the EoI, the project officer will telephone them once to check whether they have received it.

The project officer will follow up those patients who have indicated their willingness or potential willingness to participate, answer any questions and concerns they may have about the study and ask them to arrive 30 minutes earlier for their scheduled appointment to allow time to complete the questionnaires. The PIS contains contact numbers of the academic and clinical leads for this project so patients may contact them prior to making their decision to participate. When patients arrive at the clinic for a regular appointment, they will again be given the opportunity to discuss any questions they may have regarding their participation with the project officer who will greet them, obtain their electronic consent on the iPad and show them how to complete the questionnaires on the iPad.

Patients who were recruited with Stage III melanoma but progress to Stage IV disease during the study can choose whether to continue in the study or not. Any continuing Stage IV patients will be asked to complete an

For peer review only - http://bmjopen.bmj.com/site/about/guidelines.xhtml

interview at the end of the study to provide an assessment of how helpful they found this project to be considering their changing treatment and needs.

Clinician recruitment process

Clinical staff will be informed about the project through education sessions at clinical and multi-disciplinary team (MDT) meetings and be given a written invitation to participate. Interested clinicians will be provided with an electronic PISCF to read. Either hard copies of signed consent or scanned electronic versions will be acceptable and collated by the project officer prior to the clinician seeing study patients. All clinicians who were approached to participate consented to the study and reviewed the study questionnaires with the patients.

Interventions

The study questionnaires will be repeated at subsequent regularly scheduled follow-up appointments with their clinicians, with a minimum of two months between patient completion of questionnaires. Questionnaires will be administered at baseline, with a minimum of two and a maximum of three, follow-up time points. iPads were chosen as the delivery platform because they are portable, have a user-friendly interface, are capable of hosting software that can automatically generate reports and associated documents and are a secure format for data management as they are encrypted by default. All questionnaires used in this study have been validated in previous studies.

At each visit, all patients will be required to complete two short questionnaires that are used to assess their psychosocial distress and quality of life: 1. the National Comprehensive Cancer Network's Distress Thermometer and Problem List for Patients;[19] and 2. EuroQoL's EQ-5D-5L and visual analogue scale.[20] Patients who score above the clinical cut off score on the Distress Thermometer (4 or above) will be triaged to complete an additional three questionnaires: the Melanoma Concerns Questionnaire (MCQ-28),[21] the Depression, Anxiety and Stress Scale (DASS-21)[22] and the European Organisation for Research and Treatment of Cancer (EORTC QLQ-C30).[23] These additional questionnaires will provide more detailed information about potential areas of support needs. Completion time for all five questionnaires is expected to be less than 30 minutes. All questionnaires are provided in Appendix 1.

Results from the completed questionnaires will be automatically generated, emailed to the clinic secretary and printed for the treating clinician in real-time, so the results can be discussed during their consultation, along with medical concerns. Patients who request a copy of their results (by selecting the appropriate box in the 'About You' survey) will receive a summary of their scores by email, prior to their imminent appointment. Participants who score 3 or above on any of the five domains covered in the EQ-5D-5L questionnaire[20] (mobility, self-care, usual activities, pain/discomfort and anxiety/depression) may require clinical intervention (3= moderate, 4=

For peer review only - http://bmjopen.bmj.com/site/about/guidelines.xhtml

severe, 5 = extreme limitations). There are no melanoma-specific cut-off scores indicating the need for clinical intervention, for the QoL questionnaires MCQ-28[21] and EORTC QLQ-C30.[23] Changes in scores for these two questionnaires will be recorded at each time point, allowing for manual inspection of changes over time. A deterioration in EORTC QLQ-C30 scores of 10 points or more between visits is commonly regarded as clinically significant.[24,25] Recommendations and referrals for additional support services will be based on the questionnaire scores and clinical experience of the clinicians and be made in collaboration with the patient. Further information on reporting processes is available in Appendix 2.

After all rounds of patient questionnaires (baseline plus 2-3 follow-up rounds per patient) have been completed, all patients, clinicians and clinic staff involved in the project will be asked to complete an electronic survey providing quantitative and open-ended assessments of their experiences with the PROMs questionnaires. In addition, approximately 20% of patients (10 patients), all study clinicians and clinic staff with major involvement in the study will be invited by the research team to participate in a short (10-minute) confidential interview to expand on their survey responses. Patients will be purposively sampled to maximise response variation, based on a range of individual views and experiences as identified from their survey results. Patients who complete an interview will be offered a $30 gift card to thank them for their time, while clinicians and clinic staff who participate in an interview will not be reimbursed. Figure 1 provides a study flow chart from the patient's perspective and Figure 2 provides the study overview from the clinician's perspective.

Outcomes

The primary intended outcome of this study is to determine whether completion of PROMs electronically are considered by both patients, clinicians and clinic staff as feasible and acceptable when incorporated into routine clinical care. Secondary outcomes include assessment of whether these measures help identify people who need and would benefit from further support/referral; the production of a framework for the data systems required to enable electronic data capture and feedback to clinicians, useful in many other applications and clinical specialties; and the utility of clinician training resources. The primary outcome has three components.

Patient perceptions

Patient ratings of feasibility and acceptability will be assessed in several ways. The first is by the proportion of eligible patients approached who completed baseline and at least one set of follow-up PROMs data. If 60% of patients meet this requirement, the study will be deemed feasible and acceptable overall.

Data on individual patient measures of feasibility and acceptability will also be collected. In the first set of two triage questionnaires, all questions are mandatory, but with the second set of three questionnaires, patients are not required to answer all questions. Metadata from the patients who are triaged to complete the second group

For peer review only

For peer review only - http://bmjopen.bmj.com/site/about/guidelines.xhtml

of questionnaires will be collected to provide an estimate of the mean time taken to complete the PROMs questionnaires and the amount of missing data (providing a second measure of patient acceptability for those who completed all five questionnaires). If patients take more than 30 minutes to complete the five questionnaires, they will be considered as not having met this acceptability criteria. Additional measures will include changes in baseline and follow-up scores and the proportion of patients who: 'agree or strongly agree' that the PROMs were easy to complete and that the questions they completed measured issues they considered important to their QoL (Appendix 3a). Semi-structured interviews with patients will also assess feasibility and acceptability through the following main question themes: enjoyment of completing the PROMs; changes in patient-clinician communication; changes in their management; and improvements or challenges they found in the processes.

Clinician perceptions

It is also important to assess the feasibility and acceptability of this project from the clinicians' perspective. Measures include whether they found the PROMs data provided to them to be useful, timely, easy to understand and appropriate for their patient population. Clinicians will be approached to expand on their survey responses (Appendix 3b) in a semi-structured interview. The key themes to address are related specifically to the PROMs instruments and the information generated for the clinician; changes in patient management; improvements in patient-clinician communication; and any key challenges or improvements that they can identify regarding the processes.

Clinic staff perceptions

Clinic staff including clinical nurse consultants and medical secretaries will also be asked to assess the feasibility and acceptability of incorporating QoL questionnaires into their clinic's routine practice (Appendix 3c). If they consider the additional workload or clinic disruption to be unacceptable or burdensome, then this will have a major impact on the feasibility assessment of continuing this practice once the pilot study has been completed.

Secondary outcomes

Research and clinical psychologists at MIA will use identified unmet needs from this study to inform future research into the development of support programs at MIA. The extent to which the routine use of PROMs in clinical practice can improve patients' emotional and physical problems will be assessed through changes in referral rates, referral uptake rates and patients' QoL scores over time. Patient and clinician interviews will allow for more extensive evaluation of this aspect of the study.

For peer review only - http://bmjopen.bmj.com/site/about/guidelines.xhtml

The utility of this framework for the data systems required to enable electronic data capture and feedback to clinicians will be assessed in patient, clinician and clinic staff surveys and interviews as well as assessment by the project team. End of study surveys will highlight the perceived ease of use, usefulness, relevance and importance of electronic PROMs to each group (Appendix 3). Data will also be collected on time taken to complete questionnaires and on the number of times the questionnaires were completed (out of a maximum of three to four time points). In addition, the number of questions answered in the second set of three questionnaires (all questions in the first set of two questionnaires are mandatory) will be recorded.

All participating clinicians are experienced in treating Stage III melanoma patients and will be asked to review resources prepared for this study. These include three short videos about PROMs, referral pathways and the project details, and support services referral information on the MIA intranet. The aim of these resources is to help clinicians familiarise themselves with the objectives of the study and the use of PROMs in an oncology setting. Appendix 4 contains a list of study investigators. Table 1 provides a timeline of expected outcomes.

For peer review only

Table 1. Study interventions and assessments timeline

	Baseline			End of study
**TIME POINTS[1]**	T0	T1	T2	T3
ENROLMENT				
Eligibility screen	X			
Informed consent	X			
Recruitment	X			
INTERVENTIONS				
Baseline patient questionnaires	X			
Baseline patient demographic info	X			
Follow-up patient questionnaires		X	X	X
Clinician completion surveys				X
Patient completion surveys				X
Clinician completion interviews				X
Patient completion interviews				X
Clinician referral data				X
Patient referral uptake data				X
Patient change over time (T0-T5)				X
ASSESSMENTS				
Clinician acceptance				X
Patient acceptance				X
Clinic staff acceptance				X
Referral rate				X
Referral uptake rate				X

Table 1 adapted from the template designed by the SPIRIT Group ©2013[26]
1. For each time point, a window of +/- 2 months is acceptable due to variation in routine clinical appointments.

The overall success of the project will be assessed using the Proctor et al. framework for implementation research[27] which considers not only the intervention outcomes (service and client) but also the implementation outcomes. Table 2 defines the implementation outcomes (excluding acceptability and feasibility which are described throughout the protocol) and provides examples of how they will be measured in the context of this study.

For peer review only

For peer review only - http://bmjopen.bmj.com/site/about/guidelines.xhtml

Table 2. Assessment of implementation outcomes[27]

Implementation Outcome	Definitions	Examples of Assessment Measures
Appropriateness	"the perceived fit, relevance, or compatibility of the innovation or evidence based practice for a given practice setting, provider, or consumer; and/or perceived fit of the innovation to address a particular issue or problem."	Surveys and interview questions with participating clinicians and clinic staff.
Costs	"the cost impact of an implementation effort." [not restricted to financial costs].	Costs to patients: estimates of time (need to arrive earlier for appointments) and inconvenience (e.g. heavier traffic; questionnaire fatigue).<br>Costs to clinicians: estimates of time to incorporate patient responses into discussion; opportunity costs.<br>Costs to clinic staff: estimates of time required to provide recruitment information to researchers, to print out questionnaire responses, to scan these for the patients' records.<br>Calculation of financial costs of research staff required to manage the administration and analysis of PROMs and the reporting of study outcomes.
Fidelity	"the degree to which an intervention was implemented as it was prescribed in the original protocol or as it was intended by the program developers."	Documentation of variations from original protocol requiring revisions and further ethics approval.
Adoption	"the intention, initial decision, or action to try or employ an innovation or evidence-based practice. Adoption also may be referred to as 'uptake'."	Assessment of the willingness of all participants to engage with project. Barriers and enablers of uptake will be explored in surveys and interviews.
Penetration	"the integration of a practice within a service setting and its subsystems."	Documentation of additional resources required to manage the project in the clinical setting as well as assessment of the impact of barriers to integration e.g. lack of clinical champions.
Sustainability	"the extent to which a newly implemented treatment is maintained or institutionalized within a service setting's ongoing, stable operations."	Long-term assessment of the continued use of these PROMs in study clinics and any extension to other clinics.

For peer review only - http://bmjopen.bmj.com/site/about/guidelines.xhtml

Data analysis and statistical methods

Quantitative analysis

Participant demographics and characteristics will be summarised for all participants using means and standard deviations. In addition, the consent, participation, and completion rates will be analysed. Consent will be defined as agreeing to participate in the study and returning a signed consent form; participation, as completing some of the components of the study; and completion, as completing all components of the study. Specifically, the proportion of eligible participants who completed the project tasks will be assessed to determine whether the inclusion of ePROMs are acceptable for patients undergoing follow-up treatment for stage III melanoma. Descriptive statistics will be reported for QoL and other questionnaire domain scores at baseline and follow-up time points. Referral and referral uptake rates will also be calculated. The small sample size of 50 for this pilot study precludes any sub-group analysis. Linear mixed effects modelling will be used to account for repeated measures and differential time points among participants. This is similar to analysis of longitudinal data from a clinical trial where measurements at follow up time points are missing.[28]

Qualitative analysis

This study takes an inductive approach to data analysis, with no pre-determined hypotheses about what we expect to find. Results from semi-structured patient interviews conducted at the conclusion of the project will be reported to assess patient views on the usability, benefits and limitations of the study questionnaires they completed, whether questionnaire results were discussed within the subsequent clinical consultation, and if so whether this led to any change in management. Similarly, results from end of study clinician interviews will report their views on the feasibility, acceptability, benefits, limitations and usefulness of including ePROMs into the routine care for patients within their practice. Electronic qualitative analysis software (NVivo) will be used to assist in the creation of a framework of key themes identified from the interview data that will then inform the development of an iterative thematic analysis process.[29]

Ethics and dissemination

Ethics and consent

Original ethics approval was obtained from St Vincent's Hospital Human Research Ethics Committee (HREC) on 19/09/2019 (2019/ETH10558). This approval included site authorisation for the following two project sites:

- Sydney Melanoma & Surgical Oncology, MIA, Poche Centre
- Sydney Melanoma & Surgical Oncology, RPAH, Camperdown

For peer review only

For peer review only - http://bmjopen.bmj.com/site/about/guidelines.xhtml

Amendments to the original protocol were accepted by St Vincent's HREC on 8 June 2022.

Consent is obtained electronically on the iPad from all patients prior to commencing the questionnaires, while clinical staff signed written consent forms. All participants who complete end-of-study surveys or interviews will sign additional written consent forms. There are no foreseeable additional harms, risks or costs associated with taking part in this study that are not outlined in the PIS. It is possible some patients may experience short-term distress while answering the questionnaires. If a patient does not wish to answer a question, they may skip it and go to the next question (if they are completing the second set of questionnaires), or immediately stop completing the questionnaires (if they are completing the first set of questionnaires, where all questions are mandatory). A melanoma clinical nurse consultant and/or treating clinician will be available to talk with a distressed patient if their distress continues once they have stopped completing the questionnaire. Patients are free to withdraw from the study at any time. Clinicians may experience frustration with having to review patient summary scores from the questionnaires if they are particularly busy.

Dissemination of trial results

Study results will be disseminated to participants via a short, lay summary of overall study findings at the end of the study. Patients can also opt in to receive their personal questionnaire results at each scheduled visit over the study time period. Overall study results will be communicated to participating clinicians and local healthcare professionals through multidisciplinary team meetings and to wider clinician and policy audiences through presentations to the national surgical oncology, medical oncology and nursing representative societies, as well as government bodies. In addition to the standard fora of publication in peer-reviewed journals, seminar/webinar presentations and presentations at scientific conferences, presentations and summary materials targeting consumers and their families will be developed. Such informal tailored presentations will involve our patients and their families in discussion of the study findings and implications of ongoing PROMs research for clinical practice.

Access to study data

De-identified data only will be made available through a nominated data repository on request. Public access to participant-level data will not be available due to patient privacy concerns.

Publications policy

A publications policy has been created for this study. It outlines processes for authorship and will guide the appropriate recognition of all contributors to the intellectual property developed within the study. It applies to all publications that present research arising from and/or receiving funding from the pilot study as well as any future expanded studies based on the pilot findings.

For peer review only - http://bmjopen.bmj.com/site/about/guidelines.xhtml

** ** **

Contributors

All authors participated in the drafting of this manuscript, undertook critical review of the intellectual content and approved the final version to be published. In addition, each of the authors made specific contributions as outlined below.

Kathy Dempsey was responsible for updating the original version of the protocol and drafting the first version of the manuscript, ensuring changes to the protocol were approved by the University of Sydney Human Research Ethics Committee and for coordinating co-author responses. KD is also the corresponding author and guarantor of this manuscript and a member of the ePROMs-MEL Steering Committee.

Rachael L Morton, Robyn PM Saw, Iris Bartula, Serigne Lo and Craig Lawn are Steering Committee members who were responsible for the conception and design of this protocol and study. RLM is an experienced health services researcher and health economist, IB a clinical and research psychologist, SL an accomplished biostatistician and CL has extensive consumer representative experience in melanoma. All made substantial contributions to the conception and design of this research. Frances Boyle contributed her expertise in the use of the psychometric measures, including the melanoma-specific Melanoma Concerns Questionnaire, and clinician-patient communication approaches for collected data. Skye Dong is an experienced clinical psychologist who advised on the use of PROMs in the clinical setting and played a major role in the selection of appropriate PROMs. She is also the study psychologist who clinicians refer distressed study participants to for counselling. Mbathio Dieng, an early career researcher and Donna Milne, a clinical nurse consultant, worked with the Steering Committee and second consumer representative Linda Seaman, to review the PROMs and contributed to discussions on their appropriateness, particularly in terms of optimising research yield while minimising patient and clinic burden. Jake Thompson contributed his project officer skills and experience in psychosocial research and provided assistance to patients for data collection in the clinics when required. Melanoma clinicians Thomas Pennington, Robyn Saw, Andrew Spillane, Dina Saks and Julia Lai-Kwon contributed to patient recruitment and, along with France Boyle, acted as clinical project champions for the delivery of psychological support services within these clinics.

Competing interests

RPMS has received honoraria for advisory board participation from Merck Sharp & Dohme (MSD), Novartis and Qbiotics and speaking honoraria from Bristol Myers Squibb (BMS). All other authors declare no competing

For peer review only - http://bmjopen.bmj.com/site/about/guidelines.xhtml

interests.

Funding

KD's salary is supported through the Australian National Health and Medical Research Council Centre for Research Excellence in Melanoma grant (Grant No.1135285). RPMS is supported by Melanoma Institute Australia. The project officer's salary is supported by a grant from The University of Sydney's DVCR Support Fund for COVID-19 impacted research. Support is also provided by Melanoma Institute Australia for IT programming and educational design, as well as access to the Melanoma Research Database to aid in the selection of eligible patients. The study funders had no role in, or influence on, the study design; collection, management, analysis, and interpretation of data; writing of the report; or the decision to submit the report for publication.

Acknowledgements

We would like to acknowledge the significant contribution of other colleagues to the development of this protocol: Information technology staff (Ivy Tan and Ernie Wise); former project officers (Sam Robinson and Emma Zhang).

References

1. Bantug ET, Coles T, Smith KC, Snyder CF, Rouette J, Brundage MD. Graphical displays of patient-reported outcomes (PRO) for use in clinical practice: What makes a pro picture worth a thousand words? Patient Educ Couns. 2016;99: 483-490.

2. Webster EM, Burke WM, Ware HM, Jones BA, Vattakalam R, Tergas AI, Wright JD, Hou JY. Patient reported outcomes in evaluation of chemotherapy toxicity in women with gynecologic malignancies: A pilot study. Gynecol Oncol 2018;150:487-493.

3. Bouazza YB, Chiairi I, El Kharbouchi O, De Backer L, Vanhoutte G, Janssens A et al. Patient-reported outcome measures (PROMs) in the management of lung cancer: A systematic review. Lung Cancer 2017;113:140-151.

4. Kargo AS, Coulter A, Jensen PT, Steffensen KD. Proactive use of PROMs in ovarian cancer survivors: a systematic review. J Ovarian Res 2019;12:63.

For peer review only

For peer review only - http://bmjopen.bmj.com/site/about/guidelines.xhtml

5. Basch E, Deal AM, Dueck AC, Scher HI, Kris MG, Hudis C, Schrag D. Overall survival results of a trial assessing patient-reported outcomes for symptom monitoring during routine Cancer treatment. JAMA. 2017;318(2):197–198.

6. Passik SD, Dugan W, McDonald MV, Rosenfeld B, Theobald DE, Edgerton S. Oncologists' recognition of depression in their patients with cancer. J Clin Oncol 1998; 16:1594-1600.

7. Fallowfield L, Ratcliffe D, Jenkins V, Saul J. Psychiatric morbidity and its recognition by doctors in patients with cancer. Br J Cancer 2001; 84(8): 1011–1015.

8. National Comprehensive Cancer Network (NCCN). NCCN Clinical Practice Guidelines in Oncology. Distress Management. V2.2020. March 11 2020. NCCN.org [restricted access]

9. Butow PN, Price MA, Shaw JM, Turner J, Clayton JM, Grimison P, Rankin N. [on behalf of the Psycho-Oncology Co-operative Research Group (PoCoG)]. Clinical pathway for the screening, assessment and management of anxiety and depression in adult cancer patients: Australian guidelines. Psycho-oncology 2015;24(9):987-1001.

10. Dieng M, Butow P, Costa D, Morton R, Menzies S, Mireskandari S et al. Psychoeducational intervention to reduce fear of cancer recurrence in people at high risk of developing another primary melanoma: Results of a randomized controlled trial. J Clin Oncol 2016;34(36), 4405-4414.

11. Stamataki Z, Brunton L, Lorigan P, Green AC, Newton-Bishop J, Molassiotis A. Assessing the impact of diagnosis and the related supportive care needs in patients with cutaneous melanoma. Support Care Cancer 2015;23:779–789.

12. Fu H, Teleni L, Crichton M, Chan RJ. Supportive care and unmet needs in patients with melanoma. A mixed-methods systematic review. Support Care Cancer 2020. https://doi.org/10.1007/s00520-020-05464-3

13. Kasparian NA. Psychological stress and melanoma: Are we meeting our patients' psychological needs? Clin Dermatol 2013;31:41-46

14. Cancer Institute NSW. Patient-reported measures (PRMs). https://www.cancer.nsw.gov.au/patient-reported-measures [accessed 12/5/2020]

15. Australian Commission on Safety and Quality in Health Care. Evidence on the use of PROMs in clinical practice, clinical research, quality improvement and policy development.

For peer review only - http://bmjopen.bmj.com/site/about/guidelines.xhtml

16. Fradgley EA, Byrnes E, McCarter K, Rankin N, Britton B, Clover K, Carter G, Bellamy D, Paul CL. A cross-sectional audit of current practices and areas for improvement of distress screening and management in Australian cancer services: is there a will and a way to improve? Support Care Cancer. 2020;28: 249-259.

17. Insight Economics, Melanoma Institute Australia and Melanoma Patients Australia. State of the Nation. A Report into Melanoma – A National Health Priority. Final Report; Sydney: February 2022.

18. Dunn J, Watson M, Aitken JF, Hyde MK. Systematic review of psychosocial outcomes for patients with advanced melanoma. Psychooncology. 2017;26: 1722-1731.

19. The National Comprehensive Cancer Network (NCCN®) NCCN Distress Thermometer and Problem List for Patients. Version 2.2018, 02/23/18.

20. Herdman M, Gudex C, Lloyd A et al. Development and preliminary testing of the new five-level version of EQ-5D (EQ-5D-5L). Quality of Life Research 2011;20(10):1727-1736.

21. Winstanley J, Boyle F, Young T. Melanoma Concerns Questionnaire© [MCQ-28] Version 1. 31 Jan 2020.

22. Lovibond SH and Lovibond PF. (1995). Manual for the Depression Anxiety Stress Scales. (2nd. Ed.) Sydney: Psychology Foundation.

23. European Organisation for Research and Treatment of Cancer (EORTC) Quality of Life Group. Quality of Life Questionnaire-Cancer 30 (QLQ-C30). Version 3.0

24. Cocks K, King MT, Velikova G, Fayers PM, Brown JM. Quality, interpretation and presentation of European Organisation for Research and Treatment of Cancer quality of life questionnaire core 30 data in randomised controlled trials. Eur J Cancer. 2008;44: 1793-1798.

25. Musoro JM, Bottomley A, Coens C, Eggermont AM, King MT, Cocks, K et al. EORTC Melanoma Group and EORTC Quality of Life Group. Interpreting European Organisation for Research and Treatment for Cancer Quality of life Questionnaire core 30 scores as minimally importantly different for patients with malignant melanoma. Eur J Cancer 2018;104:169-181. doi: 10.1016/j.ejca.2018.09.005

26. Calvert M, Kyte D, Mercieca-Bebber R, Slade A, Chan A-W, King MT; and the SPIRIT-PRO Group. Guidelines for Inclusion of Patient-Reported Outcomes in Clinical Trial Protocols: The SPIRIT-PRO Extension. JAMA. 2018;319(5):483-494.

For peer review only - http://bmjopen.bmj.com/site/about/guidelines.xhtml

27. Proctor E, Silmere H, Raghavan R, et al. Outcomes for implementation research: conceptual distinctions, measurement challenges, and research agenda. Administration and Policy in Mental Health and Mental Health Services Research. 2011;38(2):65-76.

28. De Livera AM, Zaloumis S, Simpson JA. Models for the analysis of repeated continuous outcome measures in clinical trials. Respirology,2014;19:155–161 doi: 10.1111/resp.12217

29. Gale NK, Heath G, Cameron E, Rashid S, Redwood S. Using the framework method for the analysis of qualitative data in multi-disciplinary health research. BMC Medical Research Methodology 2013;13:117. doi:10.1186/1471-2288-13-117.

Figure titles

Figure 1. Patient's perspective

Figure 2. Clinician's perspective

Supplementary appendices

Appendix 1. Validated questionnaires

Appendix 2. Data reporting processes

Appendix 3. End of study surveys

Appendix 4. Study investigators

For peer review only

For peer review only - http://bmjopen.bmj.com/site/about/guidelines.xhtml

ePROMS-MEL FLOW CHART

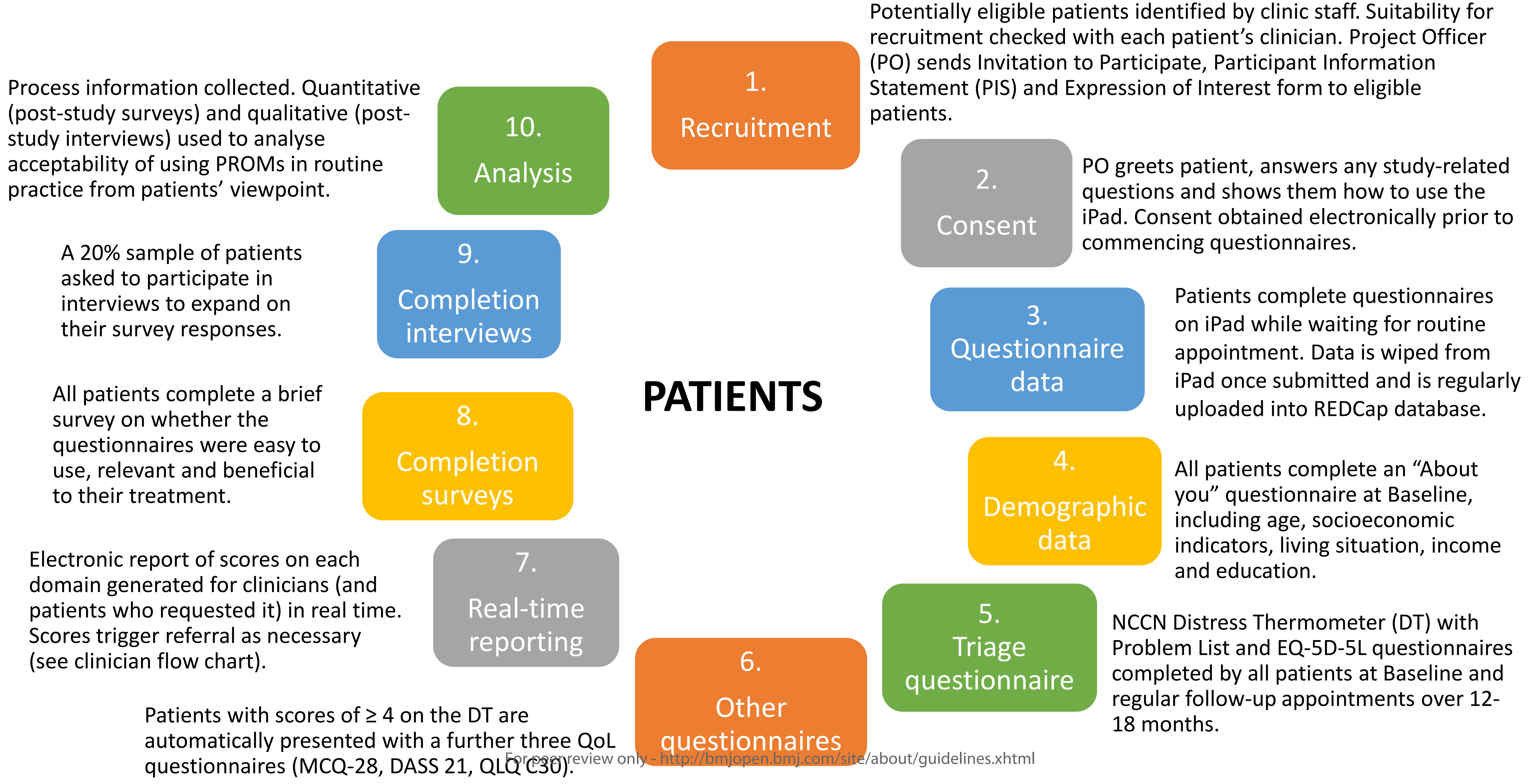

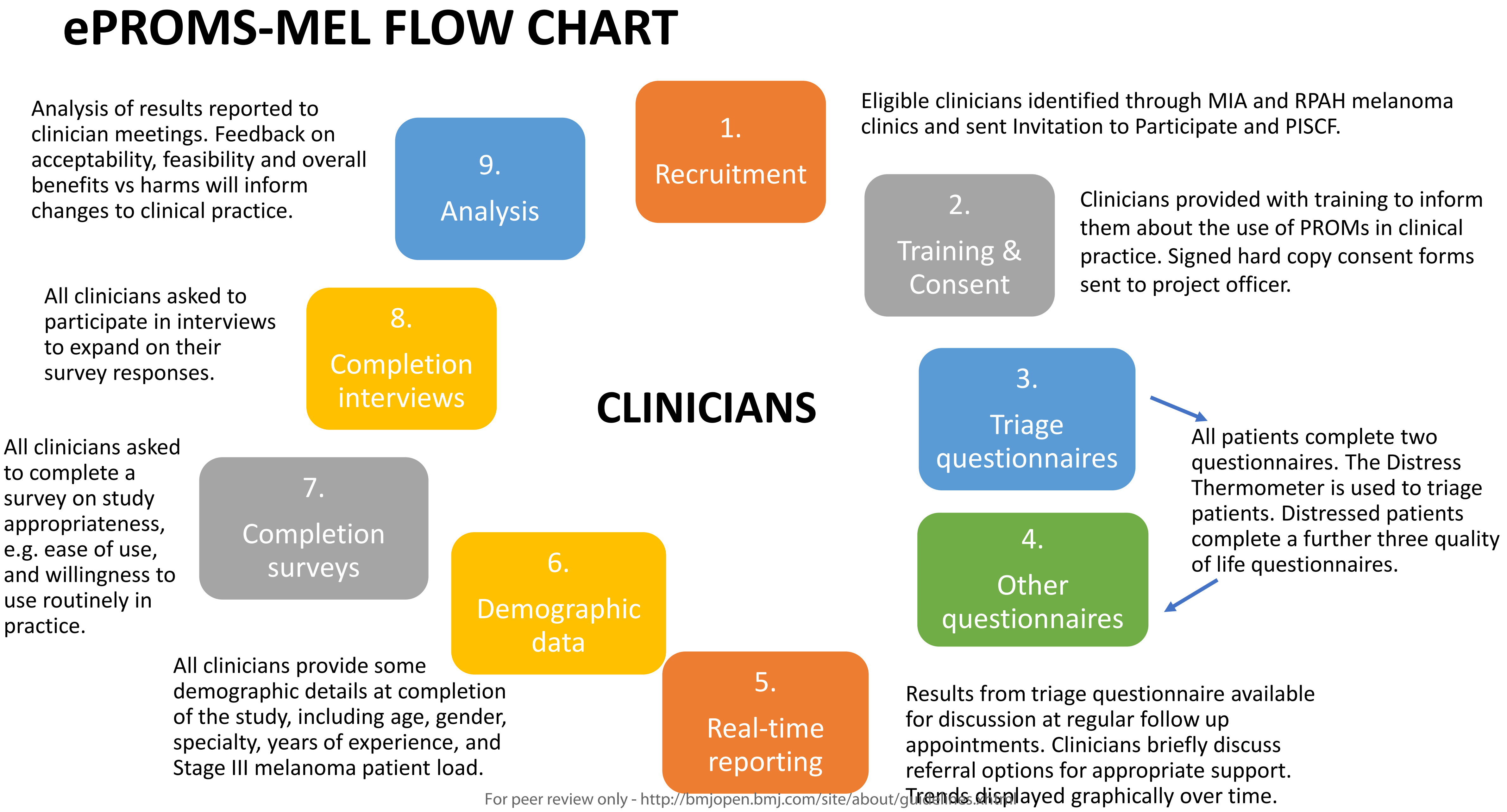
ePROMS-MEL FLOW CHART
CLINICIANS
1. Recruitment
Eligible clinicians identified through MIA and RPAH melanoma clinics and sent Invitation to Participate and PISCF.
2. Training & Consent
Clinicians provided with training to inform them about the use of PROMs in clinical practice. Signed hard copy consent forms sent to project officer.
3. Triage questionnaires
4. Other questionnaires
All patients complete two questionnaires. The Distress Thermometer is used to triage patients. Distressed patients complete a further three quality of life questionnaires.
5. Real-time reporting
Results from triage questionnaire available for discussion at regular follow up appointments. Clinicians briefly discuss referral options for appropriate support. Trends displayed graphically over time.
6. Demographic data
All clinicians provide some demographic details at completion of the study, including age, gender, specialty, years of experience, and Stage III melanoma patient load.
7. Completion surveys
All clinicians asked to complete a survey on study appropriateness, e.g. ease of use, and willingness to use routinely in practice.
8. Completion interviews
All clinicians asked to participate in interviews to expand on their survey responses.
9. Analysis
Analysis of results reported to clinician meetings. Feedback on acceptability, feasibility and overall benefits vs harms will inform changes to clinical practice.

National Comprehensive Cancer Network®

APPENDIX 1

NCCN Distress Thermometer and Problem List for Patients

NCCN DISTRESS THERMOMETER

Instructions: Please circle the number (0–10) that best describes how much distress you have been experiencing in the past week including today.

Extreme distress

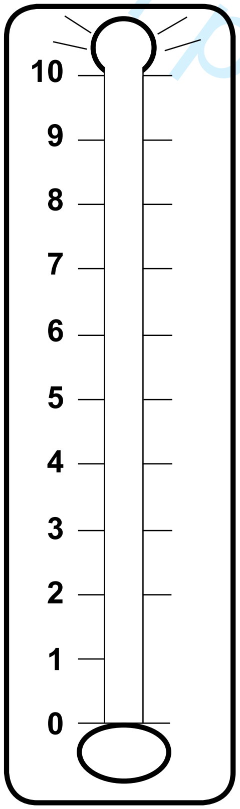

No distress

PROBLEM LIST

Please indicate if any of the following has been a problem for you in the past week including today.

Be sure to check YES or NO for each.

YES	NO	Practical Problems
❑	❑	Child care
❑	❑	Housing
❑	❑	Insurance/financial
❑	❑	Transportation
❑	❑	Work/school
❑	❑	Treatment decisions

YES	NO	Family Problems
❑	❑	Dealing with children
❑	❑	Dealing with partner
❑	❑	Ability to have children
❑	❑	Family health issues

YES	NO	Emotional Problems
❑	❑	Depression
❑	❑	Fears
❑	❑	Nervousness
❑	❑	Sadness
❑	❑	Worry
❑	❑	Loss of interest in usual activities

YES	NO	
❑	❑	**Spiritual/religious concerns**

YES	NO	Physical Problems
❑	❑	Appearance
❑	❑	Bathing/dressing
❑	❑	Breathing
❑	❑	Changes in urination
❑	❑	Constipation
❑	❑	Diarrhea
❑	❑	Eating
❑	❑	Fatigue
❑	❑	Feeling swollen
❑	❑	Fevers
❑	❑	Getting around
❑	❑	Indigestion
❑	❑	Memory/concentration
❑	❑	Mouth sores
❑	❑	Nausea
❑	❑	Nose dry/congested
❑	❑	Pain
❑	❑	Sexual
❑	❑	Skin dry/itchy
❑	❑	Sleep
❑	❑	Substance use
❑	❑	Tingling in hands/feet

Other Problems: ______________________________

____

Version 2.2018, 02/23/18. The NCCN Clinical Practice Guidelines (NCCN Guidelines®) are a statement of evidence and consensus of the authors regarding their views of currently accepted approaches to treatment. Any clinician seeking to apply or consult the NCCN Guidelines is expected to use independent medical judgment in the context of individual clinical circumstances to determine any patient's care or treatment. The National Comprehensive Cancer Network® (NCCN®) makes no representations or warranties of any kind regarding their content, use or application and disclaims any responsibility for their application or use in any way. The NCCN Guidelines are copyrighted by National Comprehensive Cancer Network®. All rights reserved. The NCCN Guidelines and the illustrations herein may not be reproduced in any form without the express written permission of NCCN. ©2018.

For peer review only

For peer review only - http://bmjopen.bmj.com/site/about/guidelines.xhtml

EQ-5D-5L (UK English sample version)

Under each heading, please tick the **ONE** box that best describes your health **TODAY**

MOBILITY

I have no problems in walking about

I have slight problems in walking about

I have moderate problems in walking about

I have severe problems in walking about

I am unable to walk about

SELF-CARE

I have no problems washing or dressing myself

I have slight problems washing or dressing myself

I have moderate problems washing or dressing myself

I have severe problems washing or dressing myself

I am unable to wash or dress myself

USUAL ACTIVITIES *(e.g. work, study, housework, family or leisure activities)*

I have no problems doing my usual activities

I have slight problems doing my usual activities

I have moderate problems doing my usual activities

I have severe problems doing my usual activities

I am unable to do my usual activities

PAIN / DISCOMFORT

I have no pain or discomfort

I have slight pain or discomfort

I have moderate pain or discomfort

I have severe pain or discomfort

I have extreme pain or discomfort

ANXIETY / DEPRESSION

I am not anxious or depressed

I am slightly anxious or depressed

I am moderately anxious or depressed

I am severely anxious or depressed

I am extremely anxious or depressed

SAMPLE

For peer review only

- We would like to know how good or bad your health is **TODAY**.
- This scale is numbered from **0** to **100**.
- **100** means the best health you can imagine.
  0 means the worst health you can imagine.
- Mark an **X** on the scale to indicate how your health is **TODAY**.
- Now, please write the number you marked on the scale in the box below.

YOUR HEALTH TODAY =

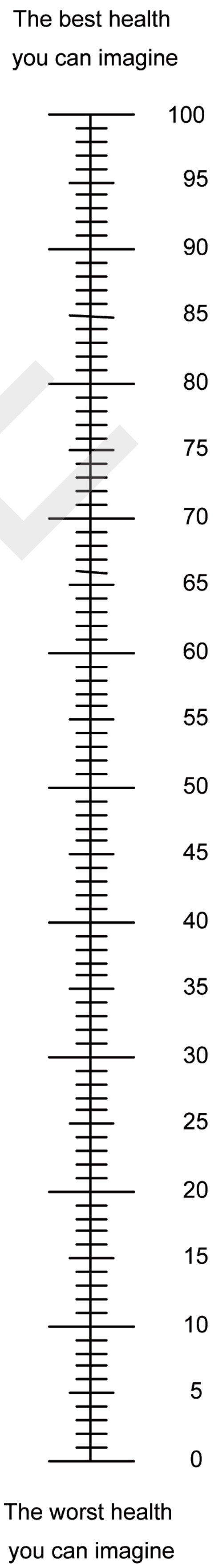

SAMPLE

For peer review only

MELANOMA CONCERNS QUESTIONNAIRE [MCQ-28] Patient Version

Patients sometimes report that they have the following concerns after their diagnosis and treatment for melanoma. Please indicate the extent to which you have experienced these symptoms or concerns.

Please answer by ticking the option that best applies to you.

	Have you had surgery for your melanoma in the last 12 months? Yes ☐ No ☐ If yes, during the past 4 weeks	Not at all	A little	Quite a bit	Very much
1	Have you had swelling near your melanoma site?	○	○	○	○
2	Have you had numbness at the site of your melanoma?	○	○	○	○
3	Have you had problems with pain at or near your melanoma site?	○	○	○	○
4	How much have you worried about complications due to your melanoma surgery?	○	○	○	○
5	How much have you worried about the length of time needed for your melanoma surgery to heal?	○	○	○	○

	Since the diagnosis and treatment of your melanoma	Not at all	A little	Quite a bit	Very much
6	Have you worried about the increased risk of melanoma for other members of your family?	○	○	○	○
7	Have you had any regrets about your exposure to sunshine in the past?	○	○	○	○
8	Have you felt concerned about conducting outdoor activities (e.g. working, swimming, walking, sport) which may result in exposure to the sun?	○	○	○	○
9	Have you considered making any significant changes to how you will live your life in the future?	○	○	○	○
10	Have you worried whilst waiting for results of medical tests?	○	○	○	○
11	Have you felt confused by information about your diagnosis or treatment from different sources (e.g. internet)?	○	○	○	○

	Since the diagnosis and treatment of your melanoma	Not at all	A little	Quite a bit	Very much
12	Have you felt hopeful for the future?	○	○	○	○
13	Have you felt able to face the challenges ahead?	○	○	○	○
14	Have you felt able to cope with your diagnosis of melanoma?	○	○	○	○
15	Have you felt able to deal with the shock of being diagnosed with melanoma?	○	○	○	○
16	Have you felt able to carry on with things as normal?	○	○	○	○
17	Have you felt able to feel positive?	○	○	○	○

	During the past 4 weeks		Not at all	A little	Quite a bit	Very much
18	If you work, have you had support from your employer to continue working during your treatment (e.g. flexible/reduced hours)	N/A	○	○	○	○
19	How much has your melanoma doctor supported you?		○	○	○	○
20	How much have your other healthcare professionals (e.g. nurse) supported you?		○	○	○	○
21	How much have your family members supported you?		○	○	○	○
22	How much has your primary care doctor supported you?		○	○	○	○
23	Have you felt able to contact the melanoma clinical staff if you needed to?		○	○	○	○
24	Have you felt confident that a psychological support service would be available if you needed it?		○	○	○	○
25	Have you been given enough time to think about the treatment options available to you?		○	○	○	○
26	Have you felt comfortable being intimate with your partner?	N/A	○	○	○	○
27	How much have those important to you been included in discussions about your treatment options?		○	○	○	○
28	Have you felt confident that your healthcare team communicate with you in a professional manner?		○	○	○	○

Depression, Anxiety and Stress Scale (DASS21)

For each statement below, please circle the number in the column that best represents how you have been feeling in the last week.

Statement	Did not apply to me at all	Applied to me to some degree or some of the time	Applied to me a considerable degree or a good part of the time	Applied to me very much or most of the time
1. I found it hard to wind down	0	1	2	3
2. I was aware of dryness of my mouth	0	1	2	3
3. I couldn't seem to experience any positive feeling at all	0	1	2	3
4. I experienced breathing difficulty (eg, excessively rapid breathing, breathlessness in the absence of physical exertion)	0	1	2	3
5. I found it difficult to work up the initiative to do things	0	1	2	3
6. I tended to over-react to situations	0	1	2	3
7. I experienced trembling (eg, in the hands)	0	1	2	3
8. I felt that I was using a lot of nervous energy	0	1	2	3
9. I was worried about situations in which I might panic and make a fool of myself	0	1	2	3
10. I felt that I had nothing to look forward to	0	1	2	3
11. I found myself getting agitated	0	1	2	3
12. I found it difficult to relax	0	1	2	3
13. I felt down-hearted and blue	0	1	2	3
14. I was intolerant of anything that kept me from getting on with what I was doing	0	1	2	3
15. I felt I was close to panic	0	1	2	3
16. I was unable to become enthusiastic about anything.	0	1	2	3
17. I felt I wasn't worth much as a person	0	1	2	3
18. I felt that I was rather touchy	0	1	2	3
19. I was aware of the action of my heart in the absence of physical exertion (eg, sense of heart rate increase, heart missing a beat)	0	1	2	3
20. I felt scared without any good reason.	0	1	2	3
21. I felt that life was meaningless	0	1	2	3

Lovibond, S.H. & Lovibond, P.F. (1995). Manual for the Depression Anxiety Stress Scales. (2nd. Ed.) Sydney: Psychology Foundation

For peer review only

ENGLISH

EORTC QLQ-C30 (version 3)

We are interested in some things about you and your health. Please answer all of the questions yourself by circling the number that best applies to you. There are no "right" or "wrong" answers. The information that you provide will remain strictly confidential.

Please fill in your initials:

Your birthdate (Day, Month, Year):

Today's date (Day, Month, Year): 31

		Not at All	A Little	Quite a Bit	Very Much
1.	Do you have any trouble doing strenuous activities, like carrying a heavy shopping bag or a suitcase?	1	2	3	4
2.	Do you have any trouble taking a long walk?	1	2	3	4
3.	Do you have any trouble taking a short walk outside of the house?	1	2	3	4
4.	Do you need to stay in bed or a chair during the day?	1	2	3	4
5.	Do you need help with eating, dressing, washing yourself or using the toilet?	1	2	3	4

For peer review only

During the past week:

		Not at All	A Little	Quite a Bit	Very Much
6.	Were you limited in doing either your work or other daily activities?	1	2	3	4
7.	Were you limited in pursuing your hobbies or other leisure time activities?	1	2	3	4
8.	Were you short of breath?	1	2	3	4
9.	Have you had pain?	1	2	3	4
10.	Did you need to rest?	1	2	3	4
11.	Have you had trouble sleeping?	1	2	3	4
12.	Have you felt weak?	1	2	3	4
13.	Have you lacked appetite?	1	2	3	4
14.	Have you felt nauseated?	1	2	3	4
15.	Have you vomited?	1	2	3	4
16.	Have you been constipated?	1	2	3	4

Please go on to the next page

ENGLISH

During the past week:

	Not at All	A Little	Quite a Bit	Very Much
17. Have you had diarrhea?	1	2	3	4
18. Were you tired?	1	2	3	4
19. Did pain interfere with your daily activities?	1	2	3	4
20. Have you had difficulty in concentrating on things, like reading a newspaper or watching television?	1	2	3	4
21. Did you feel tense?	1	2	3	4
22. Did you worry?	1	2	3	4
23. Did you feel irritable?	1	2	3	4
24. Did you feel depressed?	1	2	3	4
25. Have you had difficulty remembering things?	1	2	3	4
26. Has your physical condition or medical treatment interfered with your family life?	1	2	3	4
27. Has your physical condition or medical treatment interfered with your social activities?	1	2	3	4
28. Has your physical condition or medical treatment caused you financial difficulties?	1	2	3	4

For the following questions please circle the number between 1 and 7 that best applies to you

29. How would you rate your overall health during the past week?

1 2 3 4 5 6 7

Very poor Excellent

30. How would you rate your overall quality of life during the past week?

1 2 3 4 5 6 7

Very poor Excellent

For peer review only

© Copyright 1995 EORTC Quality of Life Group. All rights reserved. Version 3.0

APPENDIX 2.

Data from the questionnaires completed by the patients are uploaded into a custom-designed digital platform which allows instant access to the results presented in a user-friendly print-out.

This page shows how the results are displayed when a patient has identified anxiety:

Melanoma Institute Australia

Patient Reported Outcome Measures

Full Clinician Report- ePROMS-MEL Study

Patient Name: XXXXX
Date: 14-09-2022
Time Point: Baseline
Study ID: M130

Patient has reported moderate or high distress and/or poor quality of life.

Distress Thermometer score: (7/10)
(A value ≥ 4 is significant)

Patient has identified these problems from the Patient Problems List:
Work/school
Nervousness
Worry

EQ-5D-5L questionnaire results:

MOBILITY: 1 - I have no problems in walking about

SELF-CARE: 1 - I have no problems washing or dressing myself

USUAL ACTIVITIES: 1 - I have no problems doing my usual activities

PAIN / DISCOMFORT: 1 - I have no pain or discomfort

ANXIETY / DEPRESSION: 2 - I am slightly anxious or depressed

VISUAL ANALOGUE SCALE (VAS) score: 70/100
Global health rating Range = 0 - 100
0 = worst health you can imagine
100 = best health you can imagine

DASS21 questionnaire results:

DEPRESSSION: Normal

ANXIETY: Moderate

STRESS: Normal

This page shows the form for the clinician to complete.

Clinician Report – ePROMs-MEL study

Melanoma Institute Australia

Patient Name: Mrs Pilot Patient
Date: 23/02/2022
Time point: Follow-up 3
Study ID: ___________

Clinician name: __________________________

Patient identified as distressed

Distress Thermometer Score: 7/10

(≥4/10 is clinically significant)

Patient identified these problems:

- Housing
- Treatment decisions
- Sadness

Clinician record of referrals (Y/N)

Services	Clinician wants to refer	Service is available	Patient would accept referral	Patient referred directly	Patient referred via GP
Psychology					
Social work					
Physio					
OT					
Other (please specify)					
1.					
2.					

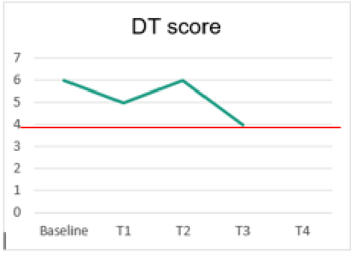

5

Full questionnaire data is uploaded from the iPad platform to a project REDCap database at the completion of recruitment and at the end of the study. This allows reports to be generated by the research team on aggregate data including trends over time, referrals and referral uptake. Reports on individual patients (e.g. to track changes in EORTC scores over time) can be generated at any time throughout the study.

APPENDIX 3a **Completionsurvey-patient**

THE UNIVERSITY OF SYDNEY

NHMRC Clinical Trials Centre

Professor Rachael Morton
Director, Health Economics & Health Technology Assessment
Deputy Director, NHMRC Clinical Trials Centre

Camperdown, NSW 2050, Australia
Tel: 61-2-9562-5000
Fax: 61-2-9565-1863
Email: Rachael.morton@sydney.edu.au

Study ID number. ____________

Please circle/select the appropriate response.

1. Do you agree the questionnaires were easy to complete?

0	1	2	3	4
Strongly Disagree	Disagree	Neutral	Agree	Strongly Agree

Please provide reasons for your choice

____

____

____

____

2. Do you agree the questionnaires you completed measured the things that you consider important to your quality of life?

0	1	2	3	4
Strongly Disagree	Disagree	Neutral	Agree	Strongly Agree

Are there other questions you would have preferred to be asked in relation to your quality of life?

____

____

____

____

3. Do you agree your “whole of person” health and welfare has been improved because of the collection and consideration of the data on a real time basis with your clinician?

0	1	2	3	4
Strongly Disagree	Disagree	Neutral	Agree	Strongly Agree

For peer review only

Please provide reasons for your choice

____
____
____
____

4. Do you think your “whole of person” health and welfare has been improved because of the availability of support resources and groups? (0 = completely agree; 4 = completely disagree)

0	1	2	3	4
Strongly Disagree	Disagree	Neutral	Agree	Strongly Agree

Please provide reasons for your choice

____
____
____
____

5. Would you be willing to be contacted by the research team for a 10-20 minute confidential face to face or telephone interview to discuss your thoughts about the questions that you completed?

Yes No

If yes: Please provide your contact details below and a member of the research team will be in touch with you soon to discuss this further, and to provide you with a Participant Information Sheet and Consent Form for the interview.

Phone number: __________________________

Email address: ____

THANK YOU FOR COMPLETING THIS SURVEY.

Your time and cooperation is highly valued. We will keep you updated about the results.

The ePROMs-MEL team

.

For peer review only

APPENDIX 3b **Completion survey - clinician**

Professor Rachael Morton
Director, Health Economics & Health Technology Assessment
Deputy Director, NHMRC Clinical Trials Centre

Camperdown, NSW 2050, Australia
Tel: 61-2-9562-5000
Fax: 61-2-9565-1863
Email: Rachael.morton@sydney.edu.au

Name: ____

Please circle/select the appropriate response

1. Age group: <30 31-40 41-50 51-60 >60

2. Gender: Male Female Other

3. How many years' experience do you have managing patients with stage III melanoma?

   < 1 year 1-5 years 6-10 years 11-15 years 16-20 years >20 years

4. What is your medical specialty?
   Surgeon
   Oncologist
   Nurse
   Specialist GP
   Allied Health (please specify) ______________________
   Other (please specify) ______________________

5. Currently, how many patients with stage III melanoma do you manage per year?

   <20 21-50 51-100 >100

6. Do you agree the results of the PROMs were useful?

0	1	2	3	4
Strongly Disagree	Disagree	Neutral	Agree	Strongly Agree

Please provide reasons for your choice

____
____
____
____

7. Do you agree the format of information provided to you was easy to read and understand?

0	1	2	3	4
Strongly Disagree	Disagree	Neutral	Agree	Strongly Agree

If not – how could this be improved?

8. Do you agree that the questionnaires were appropriate for your patients' Stage of disease?

0	1	2	3	4
Strongly Disagree	Disagree	Neutral	Agree	Strongly Agree

If not appropriate – why not?

9. Do you agree the questionnaires were adequately detailed to capture your patients' concerns related to quality of life or current health status?

0	1	2	3	4
Strongly Disagree	Disagree	Neutral	Agree	Strongly Agree

If not – what additional questions or topics would you like to be included in future sets of ePROMs / ePREMs?

10. Would you consider implementing ePROMs / ePREMs for other patients that you manage?

Yes No

If yes – for what other patient populations would you consider implementing ePROMs/ ePREMs? *For example, stage II, stage IV etc.*

For peer review only

____

If no – why would you not implement ePROMs/ ePREMs?

11. Overall, do you agree that incorporating ePROMs / ePREMs in routine care for patients with stage III melanoma is feasible?

0	1	2	3	4
Strongly Disagree	Disagree	Neutral	Agree	Strongly Agree

If agree or strongly agree: What factors make incorporating ePROMs / ePREMs in routine care for patients with stage III melanoma feasible?

If disagree or strongly disagree: Why do you believe that incorporating ePROMs / ePREMs in routine care for patients with stage III melanoma is not feasible?

12. Would you be willing to be contacted by the research team for a 10-20 minute confidential interview to discuss the feasibility and practicality of incorporating ePROMs/ ePREMs into routine care for patients with stage III melanoma?

Yes No

If yes: Please provide your contact details below and a member of the research team will be in touch with you soon to discuss this further, and to provide you with a Participant Information Sheet and Consent Form for the interview.

Phone number:______________________

Email address:____

THANK YOU FOR PARTICIPATING IN THIS STUDY – we know how busy you are!
Your time and cooperation is highly valued. We will keep you updated about the results.

The ePROMs-MEL team.

For peer review only

APPENDIX 3c **Completion survey - clinic staff**

THE UNIVERSITY OF SYDNEY

NHMRC Clinical Trials Centre

Professor Rachael Morton
Director, Health Economics & Health Technology Assessment
Deputy Director, NHMRC Clinical Trials Centre

Camperdown, NSW 2050, Australia
Tel: 61-2-9562-5000
Fax: 61-2-9565-1863
Email: Rachael.morton@sydney.edu.au

Please circle/select the appropriate response

1. Which clinic do you work in? (you may select more than one)

   SMSO Poche

   SMSO RPAH

2. Did you find the ePROMs project disruptive to clinic processes?

0	1	2	3	4
Strongly Disagree	Disagree	Neutral	Agree	Strongly Agree

   If yes, in what ways?

   ____
   ____
   ____
   ____

3. Do you think the patients benefited from the project?

0	1	2	3	4
Strongly Disagree	Disagree	Neutral	Agree	Strongly Agree

   If yes, in what ways?

   ____
   ____
   ____

For peer review only

4. Would you be pleased if ePROMs became part of routine care in your clinic?

0	1	2	3	4
Strongly Disagree	Disagree	Neutral	Agree	Strongly Agree

If no, why not?

____
____
____

5. Can you suggest changes that could be made to the project that would minimise clinic disruption?

Yes No

If yes, please share your suggestions below.

____
____
____
____

6. Would you consider implementing ePROMs for all patients in your clinic if additional resources were available?

Yes No

If yes, what additional resources would you want?

____
____
____

7. Would you be willing to be contacted by the research team for a 10-20 minute confidential interview to discuss the feasibility and practicality of incorporating ePROMs/ ePREMs into routine care for patients with stage III melanoma?

Yes No

If yes: Please provide your contact details below and a member of the research team will be in touch with you soon to discuss this further, and to provide you with a Participant Information Sheet and Consent Form for the interview.

Phone number:______________________

Email address:____

THANK YOU FOR PARTICIPATING IN THIS STUDY – we know how busy you are!
Your time and cooperation is highly valued. We will keep you updated about the results.
The ePROMs-MEL team.

For peer review only

APPENDIX 4. ePROMs-MEL Investigators

Professor Rachael Morton* (Chief Investigator) Rachael.Morton@ctc.usyd.edu.au	Director of Health Economics, NHMRC Clinical Trials Centre, The University of Sydney NSW Australia & Melanoma Institute Australia (MIA) Faculty
Associate Professor Robyn Saw* Robyn.Saw@melanoma.org.au	Melanoma Surgeon, MIA Faculty, Royal Prince Alfred Hospital (RPAH), and Mater Hospitals & University of Sydney
Associate Professor Sergine Lo* Serigne.Lo@melanoma.org.au	Senior Statistician, MIA Faculty and Senior Research Fellow in Biostatistics, The University of Sydney
Dr Iris Bartula* Iris.Bartula@melanoma.org.au	Research and Clinical Psychologist, MIA
Dr Kathy Dempsey* Kathy.Dempsey@sydney.edu.au	Senior Research Fellow, Cancer Health Policy. NHMRC Clinical Trials Centre, University of Sydney
Mr Craig Lawn*	Consumer Representative
Dr Thomas Pennington Thomas.Pennington@melanoma.org.au	Melanoma Surgeon, MIA
Professor Andrew Spillane Andrew.Spillane@sydney.edu.au	Melanoma and Breast Cancer Surgeon, MIA; Professor of Surgical Oncology, Royal North Shore Hospital & University of Sydney.
Professor Georgina Long Georgina.Long@melanoma.org.au	Medical Oncologist, MIA; Co-Director, MIA
Assoc Professor Alex Menzies Alex.Menzies@melanoma.org.au	Medical Oncologist, MIA
Professor Frances Boyle Frances.Boyle@sydney.edu.au	Medical Oncologist, MIA; Director, Patricia Ritchie Centre for Cancer Care and Research, Mater Hospital, North Sydney; Professor of Medical Oncology, University of Sydney
Dr Mbathio Dieng Mbathio.Dieng@sydney.edu.au	Postdoctoral Fellow, NHMRC Clinical Trials Centre, University of Sydney
Dr Skye Dong Skye.Dong@melanoma.org.au	Clinical Psychologist, MIA
Mr Samuel Herzog Samuel.Herzog@melanoma.org.au	Project Officer, ePROMs-MEL, MIA
Ms Shahn Coburn Shahn.Coburn@melanoma.org.au	Clinical Nurse Consultant, MIA
Ms Kate Willis Kate.Willis@melanoma.org.au	Clinical Nurse Consultant, MIA
Ms Rebecca Biviano Rebecca.Biviano@melanoma.org.au	Clinical Nurse Consultant, MIA
Ms Natalie Osborne Natalie.Osborne@melanoma.org.au	Clinical Nurse Consultant, RPAH
Ms Emel Fikri Emel.Fikri@health.nsw.gov.au	Melanoma Nurse, RPAH
Dr Donna Milne Donna.Milne@petermac.org	Melanoma Nurse, Peter MacCallum Cancer Centre
Mr Jake Thompson Jake.Thompson@melanoma.org.au	Research Officer ePROMs-MEL, MIA

For peer review only
